# The effect of a programme to improve men’s sedentary time and physical activity: The European Fans in Training (EuroFIT) randomised controlled trial

**DOI:** 10.1371/journal.pmed.1002736

**Published:** 2019-02-05

**Authors:** Sally Wyke, Christopher Bunn, Eivind Andersen, Marlene N. Silva, Femke van Nassau, Paula McSkimming, Spyros Kolovos, Jason M. R. Gill, Cindy M. Gray, Kate Hunt, Annie S. Anderson, Judith Bosmans, Judith G. M. Jelsma, Sharon Kean, Nicolas Lemyre, David W. Loudon, Lisa Macaulay, Douglas J. Maxwell, Alex McConnachie, Nanette Mutrie, Maria Nijhuis-van der Sanden, Hugo V. Pereira, Matthew Philpott, Glyn C. Roberts, John Rooksby, Øystein B. Røynesdal, Naveed Sattar, Marit Sørensen, Pedro J. Teixeira, Shaun Treweek, Theo van Achterberg, Irene van de Glind, Willem van Mechelen, Hidde P. van der Ploeg

**Affiliations:** 1 Institute of Health and Wellbeing, College of Social Sciences, University of Glasgow, Glasgow, United Kingdom; 2 Department of Coaching and Psychology, Norwegian School of Sport Science, Oslo, Norway; 3 Interdisciplinary Center for the Study of Human Performance (CIPER), Faculty of Human Kinetics, University of Lisbon, Lisbon, Portugal; 4 Department of Public and Occupational Health, Amsterdam Public Health Research Institute, Amsterdam UMC, Vrije Universiteit Amsterdam, Amsterdam, the Netherlands; 5 Robertson Centre for Biostatistics, Institute of Health and Wellbeing, University of Glasgow, Glasgow, United Kingdom; 6 Department of Health Sciences, Faculty of Science, Vrije Universiteit Amsterdam, Amsterdam, the Netherlands; 7 Institute of Cardiovascular and Medical Sciences, University of Glasgow, Glasgow, United Kingdom; 8 Institute for Social Marketing, University of Stirling, Stirling, United Kingdom; 9 Centre for Public Health Nutrition Research, University of Dundee, Dundee, United Kingdom; 10 PAL Technologies Ltd., Glasgow, United Kingdom; 11 Physical Activity for Health Research Centre, the University of Edinburgh, Edinburgh, United Kingdom; 12 Radboud University Medical Center, Radboud Institute for Health Sciences, Scientific Center for Quality of Healthcare, Nijmegen, the Netherlands; 13 European Healthy Stadia Network CIC Ltd., Liverpool, United Kingdom; 14 Computer and Information Sciences, Northumbria University, Newcastle upon Tyne, United Kingdom; 15 Health Services Research Unit, University of Aberdeen, Aberdeen, United Kingdom; 16 KU Leuven, Department of Public Health and Primary Care, Leuven, Belgium; Stanford University, UNITED STATES

## Abstract

**Background:**

Reducing sitting time as well as increasing physical activity in inactive people is beneficial for their health. This paper investigates the effectiveness of the European Fans in Training (EuroFIT) programme to improve physical activity and sedentary time in male football fans, delivered through the professional football setting.

**Methods and findings:**

A total of 1,113 men aged 30–65 with self-reported body mass index (BMI) ≥27 kg/m^2^ took part in a randomised controlled trial in 15 professional football clubs in England, the Netherlands, Norway, and Portugal. Recruitment was between September 19, 2015, and February 2, 2016. Participants consented to study procedures and provided usable activity monitor baseline data. They were randomised, stratified by club, to either the EuroFIT intervention or a 12-month waiting list comparison group. Follow-up measurement was post-programme and 12 months after baseline. EuroFIT is a 12-week, group-based programme delivered by coaches in football club stadia in 12 weekly 90-minute sessions. Weekly sessions aimed to improve physical activity, sedentary time, and diet and maintain changes long term. A pocket-worn device (SitFIT) allowed self-monitoring of sedentary time and daily steps, and a game-based app (MatchFIT) encouraged between-session social support. Primary outcome (objectively measured sedentary time and physical activity) measurements were obtained for 83% and 85% of intervention and comparison participants. Intention-to-treat analyses showed a baseline-adjusted mean difference in sedentary time at 12 months of −1.6 minutes/day (97.5% confidence interval [CI], −14.3–11.0; *p* = 0.77) and in step counts of 678 steps/day (97.5% CI, 309–1.048; *p* < 0.001) in favor of the intervention. There were significant improvements in diet, weight, well-being, self-esteem, vitality, and biomarkers of cardiometabolic health in favor of the intervention group, but not in quality of life. There was a 0.95 probability of EuroFIT being cost-effective compared with the comparison group if society is willing to pay £1.50 per extra step/day, a maximum probability of 0.61 if society is willing to pay £1,800 per minute less sedentary time/day, and 0.13 probability if society is willing to pay £30,000 per quality-adjusted life-year (QALY). It was not possible to blind participants to group allocation. Men attracted to the programme already had quite high levels of physical activity at baseline (8,372 steps/day), which may have limited room for improvement. Although participants came from across the socioeconomic spectrum, a majority were well educated and in paid work. There was an increase in recent injuries and in upper and lower joint pain scores post-programme. In addition, although the five-level EuroQoL questionnaire (EQ-5D-5L) is now the preferred measure for cost-effectiveness analyses across Europe, baseline scores were high (0.93), suggesting a ceiling effect for QALYs.

**Conclusion:**

Participation in EuroFIT led to improvements in physical activity, diet, body weight, and biomarkers of cardiometabolic health, but not in sedentary time at 12 months. Within-trial analysis suggests it is not cost-effective in the short term for QALYs due to a ceiling effect in quality of life. Nevertheless, decision-makers may consider the incremental cost for increase in steps worth the investment.

**Trial registration:**

International Standard Randomised Controlled Trials, ISRCTN-81935608.

## Abstract 2

Sally Wyke and colleagues report that the EuroFIT trial, designed to improve fitness in men, improves fitness, weight, and cardiometabolic biomarkers, but not sedenetary time at 12 months.

## Author summary

**Why was this study done?:**

- Gender-sensitised lifestyle change programmes in a professional sport setting are an exciting development in men’s health promotion, with the potential to engage men who are underserved by most programmes.
- A healthy lifestyle and weight management programme delivered in professional sporting settings (Football Fans in Training [FFIT]) has been shown to be effective and cost-effective in delivering long-term weight loss in overweight and obese Scottish football fans.
- We drew on the success of FFIT to develop and evaluate the EuroFIT programme in four European countries. Whereas FFIT introduced physical activity and dietary change for weight loss, EuroFIT focused on increasing physical activity and reducing sedentary time as desirable outcomes in their own right.

**What did the researchers do and find?:**

- We conducted a randomised controlled trial (*n* = 1,113) in 15 football clubs in four countries and showed that the EuroFIT programme was effective in increasing objectively measured physical activity but not sedentary time 12 months after baseline.
- EuroFIT participants also showed improvements in diet, body weight, indicators of cardiometabolic health, well-being, and other secondary outcomes.
- EuroFIT was not cost-effective in the short term because there were no differences in quality of life because, on the measure we used, participants already had high levels of quality of life at baseline.

**What do these findings mean?:**

- Gender-sensitised lifestyle programmes delivered in professional football clubs have shown great promise in Europe and could play an important public health role in engaging underserved men.
- Changing time spent sedentary proved difficult. Future lifestyle intervention studies should attempt to ensure that participants understand the distinction between being more physically active and spending more time upright.

## Introduction

Physical activity is important in preventing chronic diseases, including cardiovascular disease, type 2 diabetes, and several cancers [1,2]. Global recommendations from the World Health Organisation (WHO) advise at least 150 minutes per week in moderate-to-vigorous physical activity. Recent estimates show that nearly one third of adults worldwide do not meet these recommendations and around 9% of premature deaths worldwide in 2008 can be attributed to lack of physical activity [2]. Not meeting the WHO physical activity recommendations costs healthcare systems globally 53.8 billion international dollars (INT$), with an additional indirect cost of INT$13.7 billion [3].

Sedentary behaviour has recently been shown to be associated with all-cause and cardiovascular mortality, independently of physical activity [4]. Sedentary behaviour is defined as any waking behaviour in a sitting, reclining, or lying posture with energy expenditure ≤1.5 metabolic equivalent tasks (METs) [5]. A meta-analysis has shown that interventions focusing primarily on physical activity have little effect on sedentary behaviour [6], and a specific focus on sedentary behaviour is needed to achieve substantial improvements in sedentary behaviour. Combining such a specific focus on sedentary behaviour in a lifestyle intervention programme with a focus on both physical activity and diet is novel, and given the contribution of all three behaviours to the burden of the world’s leading noncommunicable diseases, such a programme could have a substantial public health impact.

Men are often underrepresented in behavioural lifestyle interventions and are considered a hard-to-reach and underserved group [7]. However, many men lead an unhealthy lifestyle and are at high risk for developing noncommunicable diseases. It has been suggested that of all facets of health promotion, physical activity might be the most likely behaviour to engage men with their health. A systematic review has identified gender-sensitised physical activity programmes as a key development in men’s health promotion, with the potential to engage hard-to-reach men. The review also reported that all four identified studies that involved men engaging in physical activity with other men through professional sports resulted in increased physical activity [8]. Gender-sensitised physical activity programmes for men may also provide useful strategies in promoting other areas of men’s health. Another systematic review concluded that weight loss and maintenance for men is best achieved with interventions increasing physical activity and improving diet while using behaviour change techniques [7].

Achieving sustainable health behaviour change is challenging, and at-risk population groups, including overweight and/or inactive men, are difficult to engage and underserved. The Scottish Football Fans in Training (FFIT) lifestyle programme was designed to attract overweight men and enable them to lose weight through improvements in physical activity and diet. FFIT was shown to be cost-effective in supporting clinically significant weight loss. It also significantly improved self-reported physical activity and diet at 12 months [9], and improvements were partially maintained 42 months after baseline [10]. The multi-country European Fans in Training (EuroFIT) programme shifted the focus from weight loss to improving physical activity and sedentary time [11]. Like FFIT, EuroFIT uses the allegiance many fans have to their football club to attract at-risk men to a group-based lifestyle change programme delivered in their clubs.

This paper describes the results from the randomised controlled trial that aimed to evaluate the effectiveness of the EuroFIT lifestyle programme. The primary aim of the trial is to determine whether EuroFIT can help men aged 30–65 years with a self-reported body mass index (BMI) ≥27 kg/m^2^ to increase objectively assessed physical activity and decrease objectively assessed sedentary time over a 12-month period. Secondary outcomes of the trial include cost-effectiveness, food intake, body weight, BMI, waist circumference, resting systolic and diastolic blood pressure, cardiometabolic blood biomarkers, well-being, self-esteem, vitality, and quality of life.

## Materials and methods

### Study design

We undertook a pragmatic two-arm randomised controlled trial in 15 professional football clubs from leagues in England (five clubs), the Netherlands (four clubs), Norway (three clubs), and Portugal (three clubs). Study participants were randomised to receive the intervention or a waiting list comparator (1:1), stratified by club. The study protocol is published [11].

### Recruitment and participants

Football clubs were selected by contacting clubs known by the study team to be likely to be interested in taking part. We sought a minimum of three and a maximum of five in each country, and the first 15 clubs that signed up were included. Clubs were Arsenal, Everton, Newcastle, Manchester City, and Stoke (England); Ado den Haag, Groningen, Philips Sport Vereniging (PSV), and Vitesse (the Netherlands); Rosenborg, Strømsgodset, and Vålerenga (Norway); and Benfica, Porto, and Sporting (Portugal).

Football clubs led recruitment of participants using emailed invitations to fans, the club website, social media posts, features in local press, and match-day recruitment.

Participants registered interest online, providing contact details, age, self-reported height and weight, and preferred football club. A follow-up telephone call included the adapted Physical Activity Readiness Questionnaire-Plus questionnaire (PAR-Q+) [12], previous participation in health promotion programmes at the club, and asking if men were willing to consent to randomisation and to wearing an activity monitor for 1 week at baseline and again at both follow-up assessments. On the consent form, men had the opportunity to opt into providing blood samples at the baseline and the 12-month follow-up measurements.

Men were eligible if they were aged 30–65, had a self-reported BMI of ≥27 kg/m^2^, and consented to study procedures. Men were excluded if they reported a contraindication to moderate intensity physical activity in the PARQ+ or participation in an existing health promotion programme at the club, or did not provide at least 4 days of usable activity monitor data at baseline.

### Randomisation and masking

Participants were randomly allocated to intervention or comparison groups following baseline measurement. The allocation sequence for each football club was generated by a computer programme written by a statistician not involved in the final analysis. The sequence was generated using randomised permuted blocks, stratified by club, with block lengths of 4 and 6, at random. The sequence was securely stored, with access restricted to those responsible for maintaining the randomisation system.

Trial coordinators accessed randomisation allocation via a secure online portal. They informed participants by telephone and email whether they had been allocated to start the EuroFIT programme immediately (the intervention group) or to undertake the programme 12 months later (the waiting list comparison group). It was not possible to mask participants or the fieldwork team to allocation, but the primary outcome measurements could not be accessed by either, and allocation was not known by study statisticians until after database lock.

### Interventions

EuroFIT was primarily designed to support men to become more physically active, reduce their sedentary time, and maintain these changes to at least 12 months after baseline. Dietary change was also introduced for those who wanted to lose weight. The programme was delivered at club stadia to groups of 15–20 men over 12 weekly, 90-minute sessions that combined interactive learning of behaviour change techniques with graded group-based physical activity. A reunion meeting was scheduled 6–9 months after the start of the programme. To facilitate group bonding and team spirit, the same group of 15–20 men were expected to attend at the same time each week.

Details of the EuroFIT programme are published, including a description of the programme in the template for intervention description and replication (TIDieR) [13]. In brief, we developed detailed manuals for coaches and participants, and trained club coaches over 2 days to deliver programme content in an appropriate and accessible style. This included encouraging positive banter, making sessions enjoyable, promoting a ‘team’ environment, and using interactional styles congruent with other (predominantly) male contexts [14]. The programme aimed to work with rather than against predominant constructions of masculinity [9,14] whilst supporting lifestyle change. Some elements (e.g., tips to change diet or increase physical activity) were adapted to country-specific norms. Coaches were taught about the importance of warm-up activities to prevent injuries, and the programme included the Fédération Internationale de Football Association (FIFA) 11+ programme [15]. Coaches taught participants to choose from a ‘toolbox’ of behaviour change techniques (including setting and reviewing goals for behaviours and outcomes, action planning, self-monitoring, and information about health and emotional consequences of change) and to emphasise personally relevant benefits of behaviour change (e.g., being better able to fulfil valued activities and roles). These behaviour change techniques were offered as tools for men to use for however long they found them useful and to encourage men to develop internalised and self-relevant motivation for becoming more active, sitting less, and eating a healthier diet [16].

We developed a novel pocket-worn, validated device (SitFIT) [17] to allow self-monitoring of sedentary and nonsedentary time (time spent upright [18]), in addition to daily steps (S1 Appendix). In the first week of the programme, men were taught how to measure the time they spent upright and the number of steps they take each week as a baseline. In the second and subsequent weeks, they were encouraged to follow an incremental programme to set weekly goals to slowly increase the number of steps and time spent upright each week, and to use the SitFIT to monitor their progress to these goals. Evidence on the use of self-monitoring devices for physical activity after participation in the FFIT programme suggests that although some continued to find them useful in the long term, others do not, as walking and other physical activity was embedded in everyday life without self-monitoring being necessary [19].

EuroFIT also explicitly encouraged between-session and post-programme peer support for changing behaviour through interacting with each other using a social media platform most of them were familiar with (e.g., WhatsApp, Facebook Groups). They were not given specific instructions on the content of interaction; coaches could decide whether or not they participated in the interactions. Between-session group social support was also encouraged using game-based social interaction with the MatchFIT app (S1 Appendix). MatchFIT allowed participants to contribute their weekly steps to their group’s collective average step count and compare it with that of a virtual competitor team. Coaches encouraged the use of MatchFIT as a means for participants to support one another as they pursued increases in their step counts, but did not themselves participate. Programme materials are available through request at http://eurofitfp7.eu/impact/eurofit-programme/.

### Procedures

A fieldwork team collected outcome data at baseline, post-programme, and after 12 months in club stadia. They scheduled separate measurement sessions for intervention and comparison groups post-programme to minimise contamination. For participants who consented to biomarker assessment, we took a venous blood sample at baseline and 12 months, after 6 hours fasting.

To maximise attendance and retention, we made appointments by telephone, confirmed by email or letter, and sent short message service (SMS) reminders. We scheduled additional measurements either in stadia or at home as needed, but almost all men attended the regular measurement sessions. We recorded sociodemographic characteristics (age, ethnicity, education, marital status, current employment status, and income) at baseline.

In thanks for their participation in the research, we offered a club store voucher for the equivalent of €25 at post-programme and €75 at the 12-month measurements.

#### Primary outcomes

The two primary outcomes were total physical activity (steps per day) and total sedentary time (minutes per day), objectively measured 12 months after randomisation using the activPAL monitor (model activPAL micro; PAL Technologies, Glasgow, United Kingdom). The activPAL has been found to have good measurement properties to assess sitting, standing, stepping, and postural transitions in adults [20–22].

The activPAL is a small activity monitor attached to the thigh and worn for 7 consecutive full days. At the first of two on-site baseline visits, participants were shown how to fit the activPAL and how to refit it after removal. They were asked to wear the device continuously, except during water-immersing activities (e.g., swimming, bathing). Participants returned approximately 9 days later for the second on-site baseline visit, during which the activPAL was removed and the data were downloaded on a computer. Post-programme and at 12 months, researchers posted preprogrammed activPAL devices to participants 10–12 days before stadia measurement sessions, along with reminders. The standard operating procedure for preparing activPAL data for analysis is available in S2 Appendix.

#### Intervention fidelity, attendance, and experience

To assess fidelity across all clubs, researchers observed delivery of the fourth EuroFIT session and rated delivery of six key activities on a 3-point scale (1 = activity not delivered, 2 = activity adapted, and 3 = activity delivered) and the proportion of activities that scored 3 was calculated. Coaches reported weekly attendance onto a secure online portal. A post-programme questionnaire asked intervention participants to rate their overall experience of the EuroFIT programme on a 10-point scale and to rate how much they used SitFIT and MatchFIT on a scale of 0–4, where 0 was ‘not at all’, and 4 was ‘a great deal’.

#### Self-reported behavioural outcomes

Self-reported physical activity was assessed using the International Physical Activity Questionnaire (Short Form) (IPAQ) [23], self-reported sedentary time using the Marshall questionnaire [24], frequency of physically active choices using the Activity Choice Index [25], self-reported diet using an adapted Dietary Instrument for Nutrition Education (DINE) [26], and alcohol intake using a 7-day recall questionnaire.

#### Objectively measured secondary outcomes

Body weight was measured using an electronic flat scale (Tanita HD366) with light clothing. Body height was measured at baseline only without shoes, using a stadiometer (Leicester Height Measure). BMI was calculated as body weight (kilograms) divided by the square of body height (meters) (kg/m^2^). Waist circumference was measured twice (three times, if the first two measurements differed by ≥0.5 cm) using a tape measure (Seca 201), and the mean was calculated over the nearest two measurements. Blood pressure was measured with a blood pressure monitor (Omron 705-CPII) after 5 minutes sitting still.

Blood samples were stored at 4 °C and processed within 24 hours, and then frozen at −80 °C. Biochemistry tests for fasting glucose, total cholesterol, high-density lipoprotein cholesterol, triglycerides, gamma-glutamyl transferase (GGT), aspartate aminotransferase, alanine aminotransferase (ALT), hemoglobin A1c (HbA1c) (c311, Roche Diagnostics, Burgess Hill, UK), and insulin immunoassays (e411, Roche Diagnostics, Burgess Hill, UK) were run on clinically validated automated platforms. All tests used manufacturers’ reagents, calibrators, and quality-control materials. All coefficients of variation for quality controls were <5%. Homeostasis model-estimated insulin resistance (HOMA_IR_) was calculated as fasting plasma glucose (mmol.l^−1^) × fasting plasma insulin (mU.l^−1^)/22.5 [27].

#### Self-reported health and psychosocial outcomes

Participants rated their well-being using the Cantril ladder, self-esteem using the 10-item Rosenberg self-esteem questionnaire, vitality using the subjective vitality scale, and health-related quality of life using the five-level EuroQoL questionnaire (EQ-5D-5L). EQ-5D-5L utility weights were estimated using the English value set [28]. Quality-adjusted life-years (QALYs) were calculated by multiplying the utility weights with the amount of time a participant spent in a particular health state. Transitions between health states were linearly interpolated. Participants also reported joint pain and any long-standing illnesses, disabilities, or infirmities. Questionnaires are available in S3–S5 Appendices.

#### Adverse events

Serious adverse events (SAEs) were defined as any injury or newly diagnosed health condition arising during the trial study period that led to hospitalisation or prolonged medical attention, was immediately life threatening, or fatal. Events were reported by coaches or participants by email or telephone and during follow-up measurement, then followed up by telephone to gather further details. The likelihood of an event being related to EuroFIT was assessed by participants and research staff and arbitrated by the Data Monitoring and Ethics Committee.

#### Costs

Costs were measured from the societal perspective and included programme delivery, healthcare utilisation, medication use, and absenteeism from work. Unit costs (£, 2016) from the UK were used to value healthcare utilisation and absenteeism [29,30]. Programme delivery costs were calculated using costs reported by participating football clubs (i.e., preparation, coordination and administration, recruitment, programme delivery and staffing, and materials). Costs for non-UK football clubs and universities were converted to British pounds using purchasing power parities [31].

### Sample size calculation

With two primary outcomes, sample size calculations were based on achieving 90% power at a 2.5% significance level. In order to detect an effect size of 0.25 standard deviation (SD) units, a sample size of 399 per group was required. For physical activity (SD approximately 4,000 steps per day), this equates to an average increase of 1,000 steps/day. For sedentary time (SD almost 100 minutes/day [32]), this equates to an average decrease in sitting time of 25 minutes/day. To achieve almost 800 men with outcome data at 12 months, we estimated we would need to randomise 1,000 participants.

### Statistical analysis

Continuous data are summarised as mean and SD, median and interquartile range (IQR), or mean and standard error (SE) for multiply-imputed data in the cost-effectiveness analyses. Categorical data are summarised as frequencies and percentages. Outcomes post-programme and at 12 months were analyzed using linear mixed-effects regression models, adjusted for randomised group and baseline value of the outcome measure as fixed effects, and football club and country as random effects. Model residual distributions were examined graphically, and data were transformed as necessary. All analyses were intention-to-treat.

Baseline data were summarised by randomised group and for those who did or did not provide outcome activPAL data at the post-programme and 12-month assessment points, to assess the representativeness of those who provided outcome data for analysis.

Sensitivity analyses were carried out for analyses of the two primary outcomes and for body weight: (a) multiple imputation of missing baseline data, (b) repeated measures analysis, using data from all three time points in the same model, and (c) analyses to account for waking wear time (the duration for which the activPAL device was worn whilst the participant was awake).

For repeated measures analyses, data from all three time points (baseline, post-programme, and 12 months) were included as outcomes; fixed effects were included for randomised group, time point, football club, and a randomised group-by-time interaction. A random participant effect was included, and a general (unstructured) covariance structure was allowed for model residuals across the three time points. Intervention effects at post-programme and 12 months were estimated using the interaction terms from these models.

Two methods were used to account for waking wear time. First, the primary analysis models were repeated using the mean number of steps per hour and the percentage of waking time spent sedentary as outcome variables. Second, the repeated measures analyses described above were repeated with waking wear time included as a fixed effect.

For the primary outcomes and weight at 12 months, intervention effect heterogeneity was assessed by extending the regression models to include group-by-moderator interaction terms. Moderating factors considered were age, marital status, years of education, employment status, income, club, country, baseline BMI, long-standing illness, and pain in upper and lower joints.

All *p*-values are two-sided. For the primary outcomes, *p*-values <0.025 are considered statistically significant. For all other analyses, no adjustment has been made for multiple comparisons, and *p*-values <0.05 are considered suggestive of true associations. The statistical analysis plan is provided in S1 Analysis Plan.

### Cost-effectiveness analysis

We used multiple imputation, using predictive mean matching to account for the skewed distribution of costs to impute missing costs and effects. We constructed 20 imputed data sets (loss of efficiency, <5%). Mixed-effects regression models estimated effect differences, and linear regression models estimated cost differences. We calculated incremental cost-effectiveness ratios (ICERs) by dividing the cost difference between the intervention and comparison groups by the effect difference. Statistical uncertainty was estimated using bias-corrected and accelerated bootstrapping with 5,000 replications and plotted on cost-effectiveness planes. Cost-effectiveness analysis (CEA) curves show the probability that the EuroFIT programme was cost-effective compared with the comparison group for a range of different ceiling ratios. The ceiling ratio is the amount of money society is willing to pay for one unit of effect extra. This ceiling ratio is set at a maximum of £30,000 per QALY by the National Institute for Health and Care Excellence (NICE). However, for other effect measures (such as steps per day), such predefined ceiling ratios are not available.

A sensitivity analysis considered cost-effectiveness from the healthcare provider’s perspective (i.e., excluding absenteeism costs). We also performed a complete case analysis to examine if imputation influenced our results.

### Public involvement

Members of the public who had experience of similar programmes were members of our Strategic Partners Advisory Board and Trial Steering Committee and shaped the development of the protocol. Others, who had no previous involvement in similar programmes and were recruited through participating football clubs, advised on the development of the EuroFIT programme, specifically in commenting on prototypes of the SitFIT device and MatchFIT app. They also commented on trial procedures in a test of our measurement procedures undertaken before baseline measurement.

### Ethics approval and consent to participate

The study was approved in each country by local ethics committees before the start of the EuroFIT study (ethics committee of the VU University Medical Center [2015.184]; Regional committees for medical and health research ethics, Norway [2015/1862]; Ethics Council of the Faculty of Human Kinetics, University of Lisbon [CEFMH 36/2015]; and Ethics Committee at the University of Glasgow College of Medicine, Veterinary and Life Sciences [UK] [200140174]). Written informed consent to participate in the study was be obtained from all participants.

### Data access

SW, CB, EA, MNS, FvN, SK, JJ, SK, PMcS, ØR, GCR, AMcC, HvdP had full access to the data. All other authors contributed to data interpretation.

### Transparency declaration

The lead author (SW) affirms that this manuscript is an honest, accurate, and transparent account of the study being reported; that no important aspects of the study have been omitted; and there were no deviations from protocol.

The trial is registered in the International Standard Randomised Controlled Trials registry as ISRCTN32677491.

## Results

**Fig 1:**
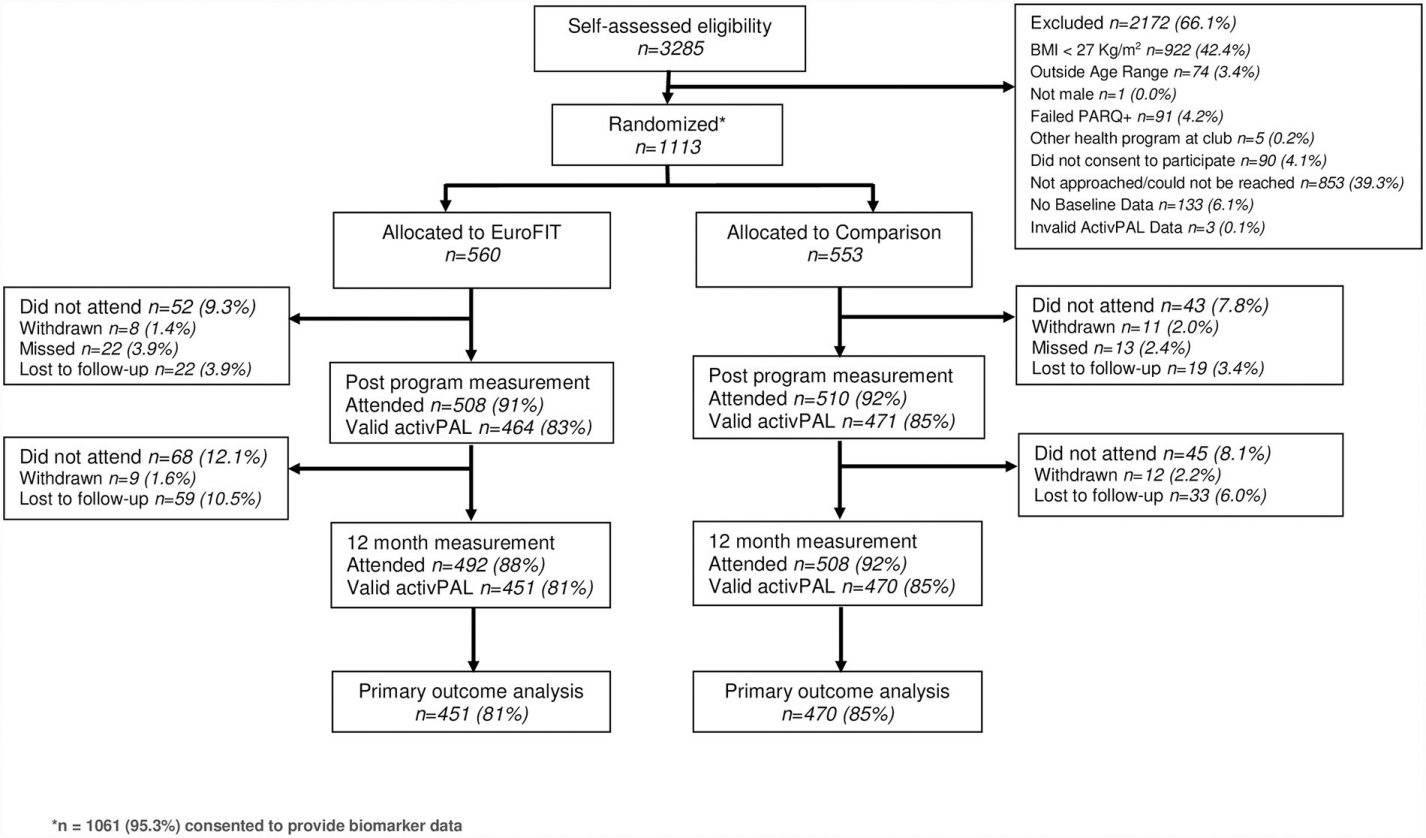
EuroFIT trial flowchart. BMI, body mass index; EuroFIT, European Fans in Training; PARQ+, Physical Activity Readiness Questionnaire-Plus.

Participants were recruited between September 19, 2015, and February 2, 2016. Participant flow through the trial is shown in Fig 1. Main reasons for exclusion for men who showed interest in the trial were BMI <27 kg/m^2^ (42.4%), inability to reach men after they expressed interest, men not being approached because the study had reached the maximum number of participants at a club (39.3%). Participants spanned all sociodemographic groups, but a majority were ‘native’ to the study country (meaning each of the participant, their mother, and their father was born there), had at least 12 years of education, were in full-time work, and were married or living with a partner (Table 1). At baseline, participants’ mean daily step count was 8,372 steps/day, sedentary time was 625 minutes/day, and BMI was 33.2 kg/m^2^.

**Table 1:** Baseline sociodemographic characteristics of participants allocated to the EuroFIT programme immediately (Intervention) or after 12 months (Comparison). Data are mean (SD), or *N* (%). *N* (missing) are reported where necessary.

Sociodemographic characteristics	Intervention (*N* = 560)	Comparison (*N* = 553)
**Age (years)**	45.9 (9.0)	45.6 (8.7)
**‘Native’ to study country (participant, mother, and father born there)**	552 (8)	545 (8)
	501 (90.8%)	482 (88.4%)
**Years of Education**	552 (8)	544 (9)
**<12 years**	137 (24.8%)	119 (21.9%)
**12–15 years**	205 (37.1%)	216 (39.7%)
**16+ years**	210 (38.0%)	209 (38.4%)
**Employment status**	551 (9)	543 (10)
**Working full time**	450 (81.7%)	432 (79.6%)
**Working part time**	32 (5.8%)	43 (7.9%)
**Not working (unable)**	27 (4.9%)	27 (5.0%)
**Not working (other)**	42 (7.6%)	41 (7.6%)
**Income**^a^	552 (8)	545 (8)
**Category 1 (low)**	36 (6.5%)	28 (5.1%)
**Category 2**	88 (15.9%)	100 (18.4%)
**Category 4**	137 (24.8%)	132 (24.3%)
**Category 5 (high)**	127 (23.0%)	123 (22.6%)
**Don’t know**	7 (1.3%)	11 (2.0%)
**Rather not answer**	44 (8.0%)	29 (5.3%)
**Relationship status**	552 (8)	545 (8)
**Married/living with Partner**	439 (79.5%)	447 (82.0%)
**Other**	113 (20.5%)	98 (18.0%)
**Long-standing illnesses**	558 (2)	549 (4)
**No**	327 (58.6%)	345 (62.8%)
**Yes, not limiting**	145 (26%)	144 (26.2%)
**Yes, limiting**	86 (15.4%)	60 (10.9%)

^a^Country-specific quintiles (low, lowest quintile of income in that country; high, highest quintile of income)

Abbreviations: EuroFIT, European Fans in Training; SD, standard deviation.

Those who provided outcome data (i.e., those who returned activPAL monitors with at least 4 valid days of measurements) were, on average, approximately 2 years older than those who did not, and slightly more likely to be married (S1 Tables, Table A). There was no clear difference in income in those who provided outcome data, nor in ethnicity, education, employment, or prevalent long-standing illness. In terms of baseline measures of study outcomes (S1 Tables, Table B), those who provided outcome were generally more active and less obese at baseline, compared with those who did not provide outcome data. This is a common feature of lifestyle intervention studies, in which those with the poorest lifestyle are hardest to engage in research.

We observed deliveries of the fourth session in 14/15 clubs. In these, coaches delivered 221 of 252 (88%) key tasks. Coaches in each of the 15 clubs provided attendance records for 553 programme participants: of these, 473 men (85.6%) attended at least 6 of the 12 sessions; 296 (53.5%) attended 10 or more sessions; and 85 (15.3%) attended all 12 sessions. Intervention participants rated their overall experience of the EuroFIT programme positively, producing a median score of 9 on a 10-point scale (IQR 8, 10; 70 missing). Asked to report their use of the SitFIT and MatchFIT, 65.1% of intervention participants reported they used the SitFIT ‘a great deal’ (score 4 on a scale of 0–4) and 36.8% reported they used MatchFIT ‘a great deal’.

**Fig 2:**
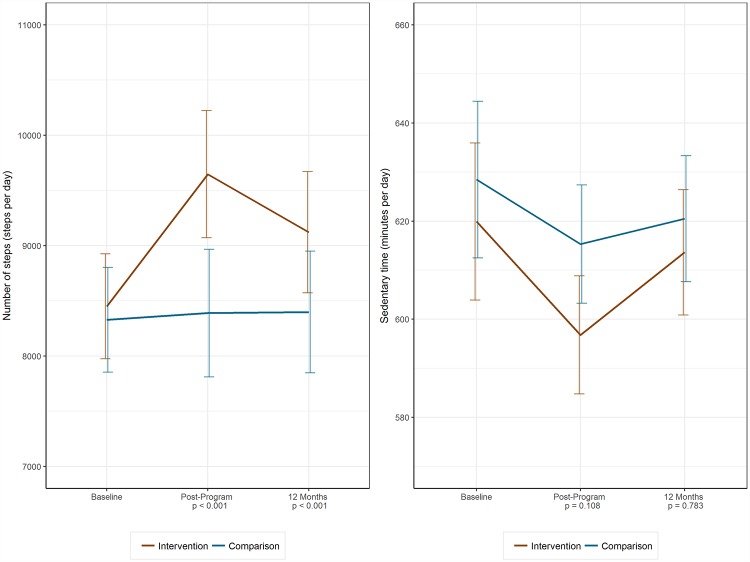
Primary outcomes (activPAL activity monitor). Model-predicted mean number of steps per day and daily sedentary time, based on repeated measures regression models.

The intervention group had a higher mean daily step count at 12 months than the comparison group (estimated difference: 678 steps/day [97.5% confidence interval (CI), 309–1,048], *p* < 0.001). There was no evidence of a difference between groups in sedentary time (estimated difference: −1.6 minutes/day [97.5% CI, −14.3–11.0], *p* = 0.77) (Table 2). In post-programme measurement, larger between-group differences in step counts (estimated difference: 1,208 steps/day [95% CI, 869–1,546]) and sedentary time (estimated difference: −14.4 minutes/day [95% CI, −25.1 to −3.8]) were observed (Fig 2). Sensitivity analyses using multiple imputations, adjusting for activPAL wear time and repeated measures analysis, showed broadly similar results (S1 Tables, Tables C, D and E).

**Table 2:** Objectively assessed physical activity and sedentary time outcome measures for participants allocated to the EuroFIT programme immediately (intervention) or after 12 months (comparison). Data are mean (SD). Intervention effects estimated are mean differences (95% CI), derived from mixed-effects regression models**.

Measures of objectively assessed physical activity and sedentary time		Intervention		Comparison		Intervention effect		
		*N*	Mean (SD)	*N*	Mean (SD)	Estimate	(95% CI)	*p*
**Number of steps (steps per day)**	**Baseline**	557	8,438 (3,211)	549	8,306 (3,146)	Difference		
	**Post-programme**	464	9,801 (3,730)	471	8,518 (3,254)	1,208	(869–1,546)	*p* < 0.001
	**12 months**	451	9,234 (3,530)	470	8,494 (3,168)	678	(309–1,048)*	*p* < 0.001
**Sedentary time (minutes per day)**	**Baseline**	557	621 (108)	549	630 (110)	Difference		
	**Post-programme**	464	597 (109)	471	613 (105)	−14.4	(−25.1 to −3.8)	*p* = 0.008
	**12 months**	451	612 (109)	470	618 (109)	−1.6	(−14.3–11.0)*	*p* = 0.772
**Number of valid days (days)**	**Baseline**	559	6.7 (0.6)	551	6.8 (0.6)	Difference		
	**Post-programme**	478	6.3 (1.2)	478	6.4 (1.0)	−0.11	(−0.25–0.02)	*p* = 0.101
	**12 months**	462	6.2 (1.1)	477	6.3 (1.0)	−0.08	(−0.21–0.05)	*p* = 0.224
**Waking wear time (minutes per day)**	**Baseline**	557	974 (69)	549	977 (68)	Difference		
	**Post-programme**	464	968 (70)	471	967 (73)	2.24	(−5.36–9.84)	*p* = 0.563
	**12 months**	451	969 (77)	470	969 (68)	0.42	(−7.63–8.48)	*p* = 0.918
**Standing time (minutes per day)**	**Baseline**	557	247 (87)	549	242 (80)	Difference		
	**Post-programme**	464	252 (85)	471	247 (83)	5.3	(−2.6–13.1)	*p* = 0.187
	**12 months**	451	244 (83)	470	244 (84)	−3.7	(−11.9–4.5)	*p* = 0.376
**Stepping time (minutes per day)**	**Baseline**	557	106 (37)	549	105 (38)	Difference		
	**Post-programme**	464	120 (41)	471	108 (39)	11.2	(7.6–14.8)	*p* < 0.001
	**12 months**	451	114 (39)	470	107 (38)	6.0	(2.4–9.6)	*p* = 0.001
**Upright time (minutes per day)**	**Baseline**	557	354 (108)	549	347 (102)	Difference		
	**Post-programme**	464	371 (106)	471	354 (104)	16.5	(6.8–26.2)	*p* < 0.001
	**12 months**	451	358 (104)	470	351 (107)	2.2	(−7.9–12.2)	*p* = 0.669

*CI (97.5%) reported for primary outcomes (12 months).

**All models of continuous outcomes were adjusted for baseline scores.

Abbreviations: CI, confidence interval; EuroFIT, European Fans in Training; SD, standard deviation.

Data summaries for participants who provided data at both baseline and post-programme, or baseline and 12 months, are provided in S1 Tables, Tables F and G.

There was no evidence that improvement in physical activity at 12 months varied by age, marital status, years of education, employment status, income, club, country, baseline BMI, long-standing illness, or pain in upper and lower joints. There was a significant interaction between the effect of the programme on sedentary time at 12 months and limiting long-standing illness (*p* = 0.034), so that those with limiting long-standing illness increased their sedentary time. There was no evidence of any other intervention effect differences between subgroups (S1 Fig, Figure A and B).

Mean body weight, BMI, waist circumference, and the proportion of participants with BMI ≥30 kg/m^2^ all improved significantly in favor of the intervention group (Table 3). The intervention effect on body weight varied by baseline BMI (interaction *p* < 0.001), with greater effects seen in those with larger BMI at baseline (S1 Fig, Figure C).

**Table 3:** Physical measures for participants allocated to the EuroFIT programme immediately (Intervention) or after 12 months (comparison). Data are mean (SD) or *N* (%). Intervention effects estimated are mean differences or odds ratios (95% CI), derived from mixed-effects regression models**.

Physical measures		Intervention		Comparison		Intervention effect		
		*N*	Mean (SD)	*N*	Mean (SD)	Estimate	(95% CI)	*p*
**Weight (kg)**	**Baseline**	559	105.3 (17.5)	550	106.5 (17.7)	Difference		
	**Post-programme**	500	102.2 (16.7)	504	105.8 (17.5)	−2.6	(−3.1 to −2.1)	*p* < 0.001
	**12 months**	484	101.8 (16.6)	501	105.7 (18.4)	−2.4	(−3.1 to −1.7)	*p* < 0.001
**BMI (kg/m**^**2**^**)**	**Baseline**	559	33.1 (4.6)	550	33.4 (4.7)	Difference		
	**Post-programme**	500	32.1 (4.4)	504	33.3 (4.7)	−0.8	(−1.0 to −0.7)	*p* < 0.001
	**12 months**	484	32.0 (4.4)	501	33.2 (5.0)	−0.8	(−1.0 to −0.5)	*p* < 0.001
**Waist circumference (cm)**	**Baseline**	559	111.0 (12.0)	550	111.6 (12.5)	Difference		
	**Post-programme**	502	107.3 (11.9)	507	110.9 (12.5)	−3.3	(−3.8 to −2.7)	*p* < 0.001
	**12 months**	480	107.6 (12.3)	503	110.9 (13.0)	−2.7	(−3.4 to −1.9)	*p* < 0.001
**BMI (≥30kg/m**^**2**^**)**	**Baseline**	559	398 (71.2%)	550	415 (75.5%)	Odds ratio		
	**Post-programme**	500	320 (64.0%)	504	383 (76.0%)	0.3433	(0.23–0.47)	*p* < 0.001
	**12 months**	484	308 (63.6%)	501	366 (73.1%)	0.5756	(0.38–0.82)	*p* = 0.003
Loss of at least 5% body weight*						Odds ratio		
	**Post-programme**	499	109 (21.8%)	504	32 (6.3%)	4.47	(2.91–6.87)	*p* < 0.001
	**12 months**	483	121 (25.1%)	501	60 (12.0%)	2.58	(1.82–3.65)	*p* < 0.001

*Models adjusted for baseline weight (kg).

**All models of continuous outcomes were adjusted for baseline scores, and all logistic regression models for binary outcomes were adjusted for the presence of the measure at baseline, with the exception of >5% weight loss, which was adjusted for weight at baseline as a continuous covariate.

Abbreviations: BMI, body mass index; CI, confidence interval; EuroFIT, European Fans in Training; SD, standard deviation.

All self-reported behaviours, including diet, improved post-programme and at 12 months in favor of the intervention, except alcohol intake, which improved only at 12 months (Table 4). In contrast to objective measurements, self-reported sitting time at 12 months significantly decreased in the intervention group compared with comparison.

**Table 4:** Self-reported behavioural outcomes for participants allocated to the EuroFIT programme immediately (intervention) or after 12 months (comparison). Data are mean (SD) or *N* (%). Intervention effects estimated are mean differences or odds ratios (95% CI), derived from mixed-effects regression models***.

Self-reported behavioural outcomes		Intervention		Comparison		Intervention effect		
		*N*	Mean (SD)	*N*	Mean (SD)	Estimate	(95% CI)	*p*
Total physical activity (IPAQ) (MET-minutes per week)**	**Baseline**	557	2,254 (2,686)	549	2,371 (2,797)	Difference		
	**Post-programme**	499	3,717 (3,307)	505	2,741 (2,951)	1,020	(691–1,348)	*p* < 0.001
	**12 months**	489	3,523 (3,158)	504	2,670 (2,899)	894	(571–1,216)	*p* < 0.001
**Recommended activity (IPAQ) (MVPA ≥ 150 minutes per week)**	**Baseline**	557	251 (45.1%)	549	255 (46.4%)	Odds ratio		
	**Post-programme**	499	338 (67.7%)	505	269 (53.3%)	1.98	(1.51–2.60)	*p* < 0.001
	**12 months**	489	310 (63.4%)	504	249 (49.4%)	1.90	(1.45–2.49)	*p* < 0.001
**Sitting time (Marshall) (hours per day)**	**Baseline**	552	11.3 (4.4)	545	11.2 (4.0)	Difference		
	**Post-programme**	490	10.4 (4.0)	495	11.3 (4.1)	−0.85	(−1.31 to −040)	*p* < 0.001
	**12 months**	487	10.1 (3.8)	503	11.1 (4.0)	−1.06	(−1.50 to −0.61)	*p* < 0.001
**Activity Choice Index (range 1–5)**	**Baseline**	495	2.4 (0.7)	484	2.4 (0.7)	Difference		
	**Post-programme**	448	3.2 (0.7)	452	2.5 (0.7)	0.66	(0.58–0.74)	*p* < 0.001
	**12 months**	437	3.0 (0.7)	447	2.5 (0.7)	0.44	(0.36–0.52)	*p* < 0.001
**Fatty food score (range 6.5–66.5)**	**Baseline**	554	18.9 (5.4)	547	18.9 (5.5)	Difference		
	**Post-programme**	498	16.5 (5.5)	505	18.1 (5.8)	−1.65	(−2.26 to −1.04)	*p* < 0.001
	**12 months**	488	16.9 (4.9)	503	18.3 (5.6)	−1.40	−1.97 to −0.84)	*p* < 0.001
**Sugary food score (range 3–18)**	**Baseline**	554	5.8 (3.2)	545	5.9 (3.4)	Difference		
	**Post-programme**	498	4.4 (2.7)	505	5.3 (3.0)	−0.94	(−1.231 to −0.66)	*p* < 0.001
	**12 months**	488	4.6 (2.4)	503	5.3 (3.1)	−0.67	(−0.97 to −0.38)	*p* < 0.001
**Fruit and vegetable score (range 1–12)**	**Baseline**	551	4.0 (2.8)	543	3.8 (2.6)	Difference		
	**Post-programme**	498	5.2 (3.0)	504	3.9 (2.5)	1.26	(0.94–1.58)	*p* < 0.001
	**12 months**	488	4.9 (3.1)	503	3.9 (2.5)	0.96	(0.63–1.28)	*p* < 0.001
**Alcohol intake (units per week)**	**Baseline**	538	6.4 (7.9)	527	6.4 (7.9)	Difference		
	**Post-programme**	478	5.5 (7.1)	470	6.3 (8.2)	−0.65	(−1.37–0.06)	*p* = 0.073
	**12 months**	486	5.0 (6.4)	503	6.0 (8.6)	−0.96	(−1.74 to −0.18)	*p* = 0.016

**IPAQ MET-minutes reported set maximum values at 180 minutes/day for walking, and other moderate and vigorous physical activity separately, before conversion to MET-minutes.

***All models of continuous outcomes were adjusted for baseline scores.

Abbreviations: CI, confidence interval; EuroFIT, European Fans in Training; IPAQ, International Physical Activity Questionnaire (Short Form); MET, metabolic equivalent task; MVPA, moderate to vigorous physical activity; SD, standard deviation.

The intervention also improved several cardiovascular risk biomarkers at 12 months. Systolic and diastolic blood pressure were both improved; fasting insulin and HOMA_IR_ were reduced by 15%; and fasting triglycerides, and ALT and GGT concentrations were reduced by 7%–8% (Table 5).

**Table 5:** Metabolic biomarkers for participants allocated to the EuroFIT programme immediately (intervention) or after 12 months (comparison). Data are mean (SD) or *N* (%). Intervention effects estimates are mean differences (with 95% CIs), derived from mixed-effects regression models, or geometric mean ratios (with 95% CIs) (95% CI estimates derived from mixed-effects regression models of log-transformed biomarkers).

Metabolic biomarkers		Intervention		Comparison		Intervention effect		
		*N*	Mean (SD)	*N*	Mean (SD)	Relative Estimate	(95% CI)	*p*
**Systolic blood pressure (mmHg)**	**Baseline**	559	133.6 (13.4)	549	135.4 (15.3)	Difference		
	**Post-programme**	501	130.9 (13.7)	507	132.6 (13.9)	−0.7	(−1.8–0.5)	*p* = 0.280
	**12 months**	479	131.3 (13.4)	501	133.8 (14.2)	−1.2	(−2.5–0.0)	*p* = 0.047
**Diastolic blood pressure (mmHg)**	**Baseline**	559	84.4 (9.7)	549	85.5 (10.0)	Difference		
	**Post-programme**	501	81.0 (9.2)	507	82.8 (9.5)	−0.8	(−1.6 to −0.1)	*p* = 0.035
	**12 months**	479	82.1 (9.4)	501	84.2 (9.5)	−1.2	(−2.1 to −0.4)	*p* = 0.004
**Fasting glucose (mmol/L)**	**Baseline**	506	4.56 (1.05)	494	4.62 (1.52)	Difference ratio		
	**12 months**	388	4.50 (0.93)	400	4.57 (1.35)	0.99	(0.97–1.02)	*p* = 0.643
**Fasting insulin (mmol/L)**	**Baseline**	509	19.11 (22.48)	497	19.61 (23.21)	Difference ratio		
	**12 months**	389	16.76 (18.24)	402	21.42 (27.77)	0.85	(0.78–0.94)	*p* < 0.001
**HOMA**_**IR**_	**Baseline**	506	4.1 (6.0)	493	4.9 (14.4)	Difference ratio		
	**12 months**	386	3.6 (4.6)	398	5.0 (11.4)	0.85	(0.76–0.94)	*p* = 0.002
**HbA1c (mmol/mol)**	**Baseline**	508	35.1 (7.1)	498	35.4 (8.9)	Difference ratio		
	**12 months**	386	34.6 (6.5)	399	35.7 (9.1)	0.99	(0.98–1.01)	*p* = 0.358
**Triglycerides (mmol/L)**	**Baseline**	509	2.19 (1.72)	497	2.31 (1.74)	Difference ratio		
	**12 months**	389	1.98 (1.39)	402	2.27 (1.37)	0.92	(0.87–0.98)	*p* = 0.006
**Total cholesterol (mmol/L)**	**Baseline**	509	4.96 (1.23)	497	4.98 (1.08)	Difference ratio		
	**12 months**	389	4.81 (1.09)	402	4.94 (0.99)	0.98	(0.96–1.00)	*p* = 0.064
**HDL cholesterol (mmol/L)**	**Baseline**	509	1.06 (0.3)	497	1.04 (0.28)	Difference ratio		
	**12 months**	389	1.1 (0.34)	402	1.05 (0.28)	1.02	(1.00–1.05)	*p* = 0.091
**AST (U/L)**	**Baseline**	509	30.8 (13.3)	496	31.5 (15.7)	Difference ratio		
	**12 months**	389	30.2 (28.5)	402	30.8 (12.5)	0.97	(0.93–1.01)	*p* = 0.123
**ALT (U/L)**	**Baseline**	508	37.7 (23.1)	496	38.3 (21.5)	Difference ratio		
	**12 months**	389	32.4 (16.9)	402	36.8 (20.7)	0.93	(0.88–0.98)	*p* = 0.004
**GGT (U/L)**	**Baseline**	509	42.8 (39.8)	497	45.7 (45.5)	Difference ratio		
	**12 months**	389	39.5 (51.8)	402	40.8 (32.9)	0.93	(0.88–0.97)	*p* = 0.003

Abbreviations: ALT, alanine aminotransferase; AST, aspartate aminotransferase; GGT, gamma-glutamyl transferase; HbA1c, hemoglobin A1c; HDL, high-density lipoprotein; HOMAIR, homeostasis model-estimated insulin resistance; SD, standard deviation.

The intervention significantly improved self-reported well-being, self-esteem, and vitality, but not quality of life, as measured by the EQ-5D-5L at 12 months (Table 6).

**Table 6:** Self-reported psychosocial outcomes for participants allocated to the EuroFIT programme immediately (intervention) or after 12 months (comparison). Data are mean (SD). Intervention effects estimated are mean differences (95% CI), derived from mixed-effects regression models.

Self-reported psychosocial outcomes		Intervention		Comparison		Intervention effect		
		*N*	Mean (SD)	*N*	Mean (SD)	Estimate	(95% CI)	*p*
**Well-being, Cantrill Good Life Ladder (range 0–10)**	**Baseline**	552	7.1 (1.4)	544	7.1 (1.4)	Difference		
	**Post-programme**	498	7.6 (1.2)	505	7.2 (1.4)	0.32	(0.20–0.45)	*p* < 0.001
	**12 months**	488	7.7 (1.2)	503	7.3 (1.3)	0.34	(0.20–0.47)	*p* < 0.001
**Rosenberg Self-Esteem Score (range 0–30)**	**Baseline**	553	22.1 (4.7)	546	22.0 (4.6)	Difference		
	**Post-programme**	498	23.3 (4.6)	505	22.2 (4.8)	0.96	(0.59–1.33)	*p* < 0.001
	**12 months**	488	23.8 (4.7)	503	22.3 (5.0)	1.16	(0.75–1.57)	*p* < 0.001
**Subjective Vitality Scale (range 4–28)**	**Baseline**	554	18.3 (5.2)	547	18.4 (5.2)	Difference		
	**Post-programme**	498	21.2 (4.6)	505	19.2 (5.1)	2.01	(1.50–2.51)	*p* < 0.001
	**12 months**	487	21.3 (4.8)	503	19.2 (5.4)	2.01	(1.46–2.55)	*p* < 0.001
**EQ-5D-5L Health Utility Score (range −0.285–1,000)**	**Baseline**	552	0.926 (0.1)	543	0.927 (0.9)	Difference		
	**Post-programme**	498	0.924 (0.1)	505	0.923 (0.1)	0.001	(−0.009–0.011)	*p* = 0.905
	**12 months**	487	0.920 (0.1)	502	0.923 (0.1)	−0.003	(−0.015–0.008)	*p* = 0.553

Abbreviations: CI, confidence interval; EQ-5D-5L, five-level EuroQoL questionnaire; EuroFIT, European Fans in Training; SD, standard deviation.

The intervention group reported more recent injuries and higher upper and lower joint pain scores post-programme, and a higher lower joint pain score at 12 months (Table 7).

**Table 7:** Self-reported injuries and joint pain for participants allocated to the EuroFIT programme immediately (intervention) or after 12 months (comparison). Data are *N* (%). Intervention effects estimated are odds ratios (95% CI), derived from mixed-effects regression models.

Self-reported injury or joint pain		Intervention		Comparison		Intervention effect		
		*N*	*N* (%)	*N*	*N* (%)	Estimate	(95% CI)	*p*
**Suffered a recent injury in last 3 months**	**Baseline**	558	23 (4.1%)	549	34 (6.2%)	Odds ratio		
	**Post-programme**	502	111 (22.1%)	508	57 (11.2%)	2.33	(1.64–3.32)	*p* < 0.001
	**12 months**	479	52 (10.9%)	500	43 (8.6%)	1.31	(0.84–2.02)	*p* = 0.231
Upper joint pain score, limiting activity*	**Baseline**	556	156 (28.1%)	548	132 (24.1%)	Odds ratio		
	**Post-programme**	502	162 (32.3%)	508	120 (23.6%)	1.58	(1.16–2.16)	*p* = 0.004
	**12 months**	480	158 (32.9%)	503	133 (26.4%)	1.43	(1.05–1.93)	*p* = 0.022
Lower joint pain score, limiting activity*	**Baseline**	557	123 (22.1%)	548	97 (17.7%)	Odds ratio		
	**Post-programme**	502	155 (30.9%)	508	104 (20.5%)	1.78	(1.30–2.43)	*p* < 0.001
	**12 months**	480	155 (32.3%)	501	117 (23.4%)	1.64	(1.20–2.24)	*p* = 0.002

*Limiting activity was scored as the maximum impact that any of the joints had on limiting activity, ranging from 0–4, with 0 = not at all, and 4 = a very great deal, which was dichotomised to not at all (0) or at least some impact (1–4).

Abbreviations: CI, confidence interval; EuroFIT, European Fans in Training.

Prices per cost item and unadjusted mean differences in costs between the two groups are presented in Table 8. Costs of the EuroFIT programme differed between countries, ranging from £189.5 to £267.5 per participant. There were no significant differences in any other cost categories between intervention and comparison groups except for visits to physiotherapists. There was no statistically significant difference in total societal costs.

**Table 8:** Multiple imputed, unadjusted costs used by participants allocated to the EuroFIT programme immediately (intervention) or after 12 months (comparison), and their unit costs (£, 2016) over 12-month follow-up.

Resource	Unit costs		Intervention (*n* = 560)	Comparison (*n* = 553)	Mean difference £ (95% CI)
	Unit	Unit costs or range (£)	Mean £ (SE)	Mean £ (SE)	
**GP**	Visit	31	93 (7.9)	95 (5.4)	−2 (−18–13)
**Physiotherapist**	Visit	40	155 (23.9)	84 (16.7)	71 (24–117)
**Dietician**	Visit	40	15 (3.8)	16 (2.6)	−1 (−8–7)
**Occupational therapist**	Visit	40	15 (3.7)	17 (2.6)	−2 (−9–5)
**Mental health therapist**^a^	Visit	121	49 (16.8)	50 (11.7)	−1 (−34–32)
**Complementary therapist**	Visit	48	19 (8.5)	10 (6)	9 (−11–39)
**Other healthcare professionals**	Visit	31–136	54 (18.6)	61 (13.3)	−7 (−44–29)
**Outpatient treatment**	Visit	136	135 (26.9)	116 (18.9)	19 (−34–72)
**Day treatment at hospital**	Visit	184	84 (20.5)	61 (13.9)	23 (−17–64)
**Inpatient treatment**	Per night spent at hospital	405	202 (84.5)	166 (59.5)	36 (−130–202)
**Medication**^c^	Cost per daily dose	0.06–419.62	136 (23.5)	135 (16.7)	1 (−45–47)
**EuroFIT programme**	Preparation and delivery	189.5–267.5	228	N/A	N/A
**Healthcare costs**	N/A	N/A	1,184 (131.5)	810 (89.9)	374 (116–632)
**Absenteeism**	£/hour missed	17.1	1,264 (208.9)	1,332 (155.7)	−68 (−609–339)
**Total costs**	N/A	N/A	2,447 (276.4)	2,141 (202.3)	306 (−244–855)

^a^Including social worker, psychologist, and psychiatrist.

^b^Including mainly medical specialists.

^c^Including cardiovascular, pain, inhalers, antidepressant, and other medication.

Abbreviations: CI, confidence interval; EuroFIT, European Fans in Training; GP, general practitioner; N/A, not applicable; SE, standard error.

The mean difference in QALYs between the intervention and comparison group was small and not statistically significant (Table 9). One QALY lost in the intervention group was associated with an incremental cost of £126,119 compared with the comparison group. The probability of EuroFIT being cost-effective compared with the comparison group was at most 0.13 for ceiling ratios up to 30,000 £/QALY (S2 Fig).

**Table 9:** Adjusted differences in mean costs (£, 2016) and effects (95% CI) at 12-month follow-up, and ICERs.

Analysis	ΔC (95% CI)	ΔE (95% CI)	ICER	CE plane quadrants			
Outcome	£	Units	£/Unit	NE	SE	SW	NW
**Societal perspective (main analysis)**							
QALYs	300 (−226–822)	−0.002 (−0.009–0.005)	−126,119	21	5	8	66
Number of daily steps (activPAL)	300 (−226–822)	730 (406–1,054)	0.41	87	13	0	0
Daily sedentary time (activPAL)	300 (−226–822)	1.74 (−9.8–13.3)	172	52	9	4	35
Meet physical activity guideline (IPAQ)	300 (−226–822)	0.15 (0.09–0.20)	2,056	87	13	0	0
Total weekly physical activity (IPAQ)	300 (−226–822)	920 (613–1,228)	0.33	87	13	0	0
≥5% decrease in weight	300 (−226–822)	0.14 (0.09–0.18)	2,228	87	13	0	0
**Healthcare provider perspective**							
QALYs	372 (125–625)	−0.002 (−0.009–0.005)	−156,696	27	0.5	0.5	72
Number of daily steps (activPAL)	372 (125–625)	730 (406–1,054)	0.51	99	1	0	0
Daily sedentary time (activPAL)	372 (125–625)	1.74 (−9.8–13.3)	214	61	0.5	0.5	38
Meet physical activity guideline (IPAQ)	372 (125–625)	0.15 (0.09–0.20)	2,554	99	1	0	0
Total weekly physical activity (IPAQ)	372 (125–625)	920 (613–1,228)	0.41	99	1	0	0
≥5% decrease in weight	372 (125–625)	0.14 (0.09–0.18)	2,768	99	1	0	0

Abbreviations: ΔC, mean difference in costs between the intervention and comparison; ΔE, mean difference in effects between the intervention and comparison; CE, cost-effectiveness; CI, confidence interval; EuroFIT, European Fans in Training; ICER, incremental cost-effectiveness ratio; IPAQ, International Physical Activity Questionnaire (Short Form); NE, northeast, i.e., EuroFIT is more expensive and more effective than comparison; NW, northwest, i.e., EuroFIT is more expensive and less effective than comparison; QALY, quality-adjusted life-year; SE, southeast, i.e., EuroFIT is less expensive and more effective than comparison; SW, southwest, i.e., EuroFIT is less expensive and less effective than comparison.

One additional step/day in the intervention group was associated with an incremental cost of £0.41 compared with the comparison group (equating to £410 per 1,000 extra steps/day). There was a 0.95 probability of EuroFIT being cost-effective compared with the comparison group at a ceiling ratio of £1.50 per extra step/day. One minute less sedentary time in the intervention group was associated with an incremental cost of £172 compared with the comparison group. The maximum probability of cost-effectiveness for sedentary time was 0.61 at a ceiling ratio of £1,800 per minute less sedentary time. The incremental cost of EuroFIT for an additional participant achieving a decrease in weight of at least 5% was £2,228. There was a 0.95 probability of EuroFIT being cost-effective compared with the comparison group at a ceiling ratio of £6,000 per additional participant achieving a decrease in weight of at least 5%, £1 per additional minute of physical activity, and £6,000 per additional participant meeting the physical activity guidelines.

The results of the cost-effectiveness analysis from a healthcare provider perspective (Table 8) and using complete cases only were comparable to the main analysis.

Seven SAEs were reported, six in the intervention group (diagnosis of heart disease, fractured wrist, fractured rib, two anterior cruciate ligament ruptures, and a torn meniscus) and one death in the comparison group. Five were deemed likely to be associated with EuroFIT (the fractured rib occurred during a warm-up at a EuroFIT session; the other injuries occurred during football matches organised by participants after the programme had finished, but still indirectly linked to participation in the programme).

## Discussion

### Principal findings

A large number of men expressed interest in the EuroFIT programme in each of the 15 football clubs. The programme helped participants to achieve increases in objectively measured physical activity but did not result in a lasting decrease in objectively measured sedentary time 12 months after baseline. The EuroFIT programme also helped men to improve secondary outcomes including weight, waist circumference, diet, well-being, self-esteem, and vitality. However, in the within-trial analysis the programme did not improve quality of life as measured by EQ-5D-5L and hence was not cost-effective based on QALYs.

### Strengths and weaknesses of the study

The EuroFIT programme was based on the successful weight loss and healthy living programme, FFIT and, like FFIT, had wide inclusion criteria. It had a sound theory base and logic model [13], the behaviour change technique ‘toolbox’ included those known to initiate and sustain behaviour change [33,34], and the programme drew on sociological understanding of masculinities to attract and retain participants [9,14]. EuroFIT was well regarded by participants, over 80% of whom attended at least half of the sessions. Post-programme, 65% of men in the intervention group reported using the SitFIT device to self-monitor steps and sitting time ‘a great deal’; 37% reported using the game-based MatchFIT app to encourage interaction between sessions and after the programme ended ‘a great deal’. Although EuroFIT attracted men from across the socioeconomic spectrum, the majority who took part were well educated and in paid work. With no obvious denominator population, we have no way of knowing if those attracted are representative of all men from local fan bases who support particular clubs.

There was an increase in recent injuries and in upper and lower joint pain scores post-programme, which might also explain higher physiotherapist costs observed in the intervention group. Although observations to assess overall fidelity showed that coaches delivered 88% of tasks as intended, preliminary analyses of other process evaluation data suggest that coaches sometimes delivered physical activity sessions that were more vigorous than specified and did not sufficiently emphasise warm-up and cooldown exercises. A focus during the 2-day coach training may be needed to avoid too many injuries.

The EuroFIT evaluation spanned four European countries and 15 professional football clubs, used objective measurement of physical activity and sedentary time, and retained over 80% of participants to objective 12-month outcome measurement. This suggests that the results are likely to be generalisable to other football clubs within Europe. It was not possible to blind participants to which group they were in, although physical activity and sedentary time were objectively assessed.

The men attracted to the programme already had quite high levels of physical activity at baseline (8,372 steps/day). This may have limited the room for improvement and led to underestimation of the potential effects of the programme if less active participants were recruited. It has been known for some time that recruitment of those most in need of physical activity interventions is more challenging than recruiting those who are already reasonably active [35]. It is possible that even more active, personalised approaches to recruitment [36] and limiting eligibility to those who do not achieve the recommended levels of physical activity would help to avoid an overrepresentation of more active men and would provide more opportunity for less active men to join the programme.

Another limitation is the potential for possible reactivity effects, in which participants change their physical activity and sedentary behaviours during the measurement week. Due to the unblinded nature of the study, the effectiveness of the intervention might have been overestimated if the intervention group did increase their activity levels more than the comparison group as a result of social desirability. However, no studies to date have reported on substantial reactivity effects in studies using 7-day accelerometer assessments.

### Strengths and weaknesses in relation to other studies

EuroFIT showed above average improvements in physical activity compared with systematic reviews and meta-analysis of other physical activity intervention programmes [37–39]. The findings from the EuroFIT trial reinforce those from a recent systematic review suggesting that gender-sensitised physical activity interventions in professional sports settings are a promising route for promoting men’s health [8]. The review identified several physical activity interventions in this setting; the FFIT study, designed to help overweight men lose weight through improvements in physical activity and diet, was the only large randomised controlled trial [40]. Recent long-term follow-up of participants in the FFIT study showed that improvements in weight loss and in self-reported physical activity were maintained 3.5 years after baseline [10]. The FFIT programme has been adapted for delivery Canada (in ice hockey) [41] and Australia (in Aussierules football) [42]. FFIT formed the basis for the development of EuroFIT; the success of the EuroFIT programme offers further evidence of the long-term public health potential of this approach.

The FFIT trial reported greater weight loss (4.94 kg; 95% CI, 4.0–5.9) than we found in EuroFIT (2.4 kg; 95% CI, 1.7–3.1), although improvements in self-reported physical activity were broadly comparable. These differences may be because dietary choice was introduced later in EuroFIT than in FFIT and weight loss emphasised only for those who wanted to do so. In FFIT, dietary and physical activity changes were both emphasised as ways of achieving and maintaining a healthier weight.

The focus of EuroFIT on reducing sedentary time was only successful in the short term. A systematic review showed similarly short-lived reductions in sedentary time [43], although some interventions showed effects up to 12 months. Workplace interventions have achieved larger reductions in sedentary time, although consistent long-term change has not yet been reported [44]. There are no clear, publicly known guidelines for reducing sedentary time, knowledge of the association between high levels of sedentary time and health is still not widespread, and sedentary time is often confused with physical inactivity [45]. Preliminary analyses of qualitative data from EuroFIT’s process evaluation suggest that both participants and coaches were confused by the combined messages of increasing physical activity and simultaneously increasing time spent upright. For example, the SitFIT device was liked by participants but mostly used to self-monitor stepping; few participants reported self-monitoring time spent upright. Future lifestyle intervention studies should attempt to ensure that participants understand the distinction and appreciate the benefits of decreasing sedentary time, as well as increasing physical activity.

Although the EuroFIT programme was not expensive to deliver (between £180 and £268 per participant), the lack of improvement in quality of life (as measured by the EQ-5D-5L) meant that the probability of it being cost-effective at ceiling ratios up to £30,000 per QALY was only 0.13 over a 12-month time frame. The equivalent probability for the FFIT programme, which estimated QALYs via the Short Form-12 (SF12) questionnaire rather than EQ-5D-5L, was 0.89 at the same ceiling ratio over the same time frame [40]. Although the EQ-5D-5L is now the preferred measure for cost-effectiveness analyses across Europe, baseline EQ-5D-5L utility scores were relatively high in EuroFIT (0.93), suggesting a ceiling effect that limits room for improvement in EQ-5D-5L utility scores. Whether the EuroFIT programme is considered cost-effective for physical activity and body weight at 12 months and shorter-term improvement in sedentary time depends on decision-makers’ willingness to pay for the observed improvements in these outcomes. We are in the process of developing a model of longer-term cost-effectiveness over a 5-year horizon to represent the benefits of physical activity in reducing the incidence of four chronic health conditions (colorectal cancer, type 2 diabetes, coronary heart disease, and stroke) and mortality.

### Meaning of the study

We have added to previous evidence [8,40] that suggests engaging men in physical activity through programmes that work with existing constructs of masculinity is a promising route for promoting men’s health. We have shown that, while participation in the EuroFIT programme did not result in improvement in sedentary time, it did result in improvements in physical activity, body weight, waist circumference, diet, well-being, vitality, and self-esteem and also to cardiovascular risk biomarkers.

A 678 steps/day increase in objectively measured physical activity is substantial. Objectively measured levels of physical activity are always lower than self-reported levels [46], and global physical activity recommendations are based on self-report. The association between objectively measured physical activity and health biomarkers is substantially stronger than the association with self-reported physical activity [47]. Given the observed improvements in cardiovascular risk biomarkers, EuroFIT is likely to result in important reduction in the risk of ill health if the improvement in physical activity is maintained.

Combining lessons learned from EuroFIT and its predecessor, FFIT, will allow the further refinement of evidence- and theory-based lifestyle change programmes delivered in professional sports settings.

## Supporting information

S1 CONSORT CONSORT checklist. (DOC)

S1 Appendix Description of the SitFIT device for self-monitoring physical activity and sedentary behaviour and of MatchFIT to encourage game-based social interaction. (PDF)

S2 Appendix Standard operating procedure for preparing activPAL data for analysis using automated sleep and non–wear-time algorithm. (PDF)

S3 Appendix EuroFIT self-complete baseline questionnaire. EuroFIT, European Fans in Training.
(PDF)

S4 Appendix EuroFIT self-complete post-programme questionnaire. EuroFIT, European Fans in Training.
(PDF)

S5 Appendix EuroFIT self-complete 12-month questionnaire. EuroFIT, European Fans in Training.
(PDF)

S1 Analysis Plan EuroFIT statistical analysis plan. EuroFIT, European Fans in Training.
(PDF)

S1 Tables Supplementary tables. (PDF)

S1 Fig Intervention effect heterogeneity, grouped by moderator interaction on physical activity, sedentary time, and weight. (PDF)

S2 Fig Probability of EuroFIT being cost-effective compared with the comparison group. EuroFIT, European Fans in Training.
(PDF)

## Abbreviations

ALT: alanine aminotransferase
BMI: body mass index
CEA: cost-effectiveness analysis
CI: confidence interval
DINE: Dietary Instrument for Nutrition Education
EQ-5D-5L: five-level EuroQoL questionnaire
EuroFIT: European Fans in Training
FFIT: Football Fans in Training
FIFA: Fédération Internationale de Football Association
GGT: gamma-glutamyl transferase
HbA1c: hemoglobin A1c
HOMA_IR_: homeostasis model-estimated insulin resistance
ICER: incremental cost-effectiveness ratio
INT$: international dollar
IPAQ: International Physical Activity Questionnaire (Short Form)
IQR: interquartile range
MET: metabolic equivalent task
NICE: National Institute for Health and Care Excellence
PAR-Q+: Physical Activity Readiness Questionnaire-Plus questionnaire
PSV: Philips Sport Vereniging
QALY: quality-adjusted life-year
SAE: serious adverse event
SD: standard deviation
SE: standard error
SF12: Short Form-12 questionnaire
SMS: short message service
TIDieR: template for intervention description and replication
UEFA: Union of European Football Associations
WHO: World Health Organisation
