# Correction: The effect of a programme to improve men's sedentary time and physical activity: The European Fans in Training (EuroFIT) randomised controlled trial

**DOI:** 10.1371/journal.pmed.1002772

**Published:** 2019-03-14

**Authors:** Sally Wyke, Christopher Bunn, Eivind Andersen, Marlene N. Silva, Femke van Nassau, Paula McSkimming, Spyros Kolovos, Jason M. R. Gill, Cindy M. Gray, Kate Hunt, Annie S. Anderson, Judith Bosmans, Judith G. M. Jelsma, Sharon Kean, Nicolas Lemyre, David W. Loudon, Lisa Macaulay, Douglas J. Maxwell, Alex McConnachie, Nanette Mutrie, Maria Nijhuis-van der Sanden, Hugo V. Pereira, Matthew Philpott, Glyn C. Roberts, John Rooksby, Øystein B. Røynesdal, Naveed Sattar, Marit Sørensen, Pedro J. Teixeira, Shaun Treweek, Theo van Achterberg, Irene van de Glind, Willem van Mechelen, Hidde P. van der Ploeg

There is an error in authorship contributions for author Christopher Bunn. The correct authorship contributions for Christopher Bunn should be: Data curation, Investigation, Methodology, Project administration, Supervision, Writing–original draft, Writing–review & editing
