# Supplementary material for: The effect of a programme to improve men’s sedentary time and physical activity: The European Fans in Training (EuroFIT) randomised controlled trial
Source: PLoS Med. 2019 Feb 5;16(2):e1002736. doi: 10.1371/journal.pmed.1002736 (PMC6363143; doi:10.1371/journal.pmed.1002736)
Supplement: S1 Appendix — (PDF) [file pmed.1002736.s002.pdf]

S1 Appendix. Description of the SitFIT device for self-monitoring physical activity and sedentary behaviour and of MatchFIT to encourage game-based social interaction

SitFIT

The EuroFIT team developed the SitFIT, a pocket-worn physical activity and sedentary behaviour monitor, to allow participants to actively self-monitor their daily physical activity (steps), upright time (minutes spent standing and walking), and sedentary time (percentage of time spent sedentary while wearing the device). The hardware and software was developed by EuroFIT partner PAL Technologies (PAL Technologies Ltd, Glasgow, UK) and the validity, user experience and integration into the EuroFIT lifestyle change program were activities led by the University of Edinburgh.[1]

In a pragmatic approach, participants were invited to use their SitFIT to track progress against individualized, incremental goals to increase both their daily step count *and* time spent upright. The SitFIT allowed self-monitoring of these daily goals by displaying daily steps, upright time, and a sedentary index for the current day and the previous six days (Figure S1.1-2). It also provided real-time, non-visual, haptic feedback on sedentary bout duration Each participant could obtain a more detailed historical record of his SitFIT data via computer upload (Windows and OSX were both supported) to the MatchFIT app (Figure S1.4). The SitFIT is a research-grade self-monitoring tool for use in evidence based interventions. In addition to the outcome measures disclosed to the participant for self-monitoring, it collects information on device performance and user interaction. This allows the evidence for the technology component of the intervention to be evaluated (Figure 1.3).

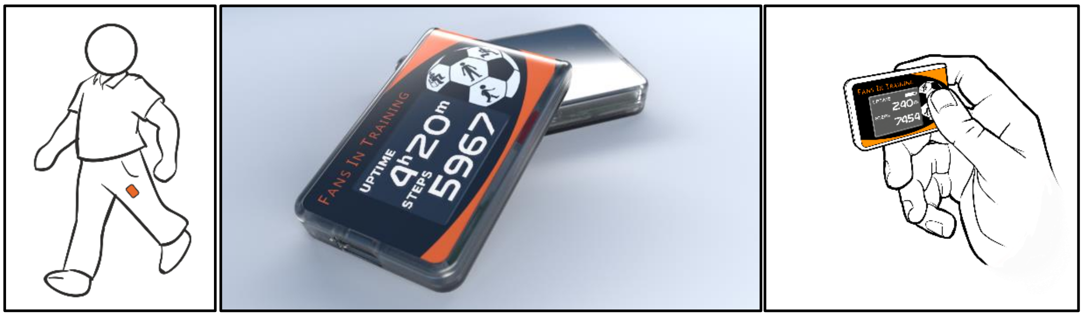

Figure 1. The SitFIT provided the wearer with feedback on their Physical Activity and Sedentary Behavior. From left to right (1) the SitFIT was worn in the trouser pocket, (2) It featured a screen for self-monitoring and (3) the screen was activated by a thumb press allowing self-monitoring behaviour to be recorded.

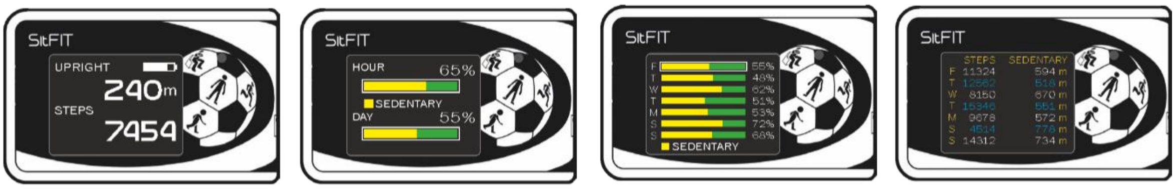

Figure 2. The SitFIT provides feedback to the wearer. From left to right (1) Real-time daily step count and upright time, (2) proportion of last hour and current day spent sitting, (3) proportion of day spent sitting for the last seven days and (4) daily totals for volume of steps and time upright.

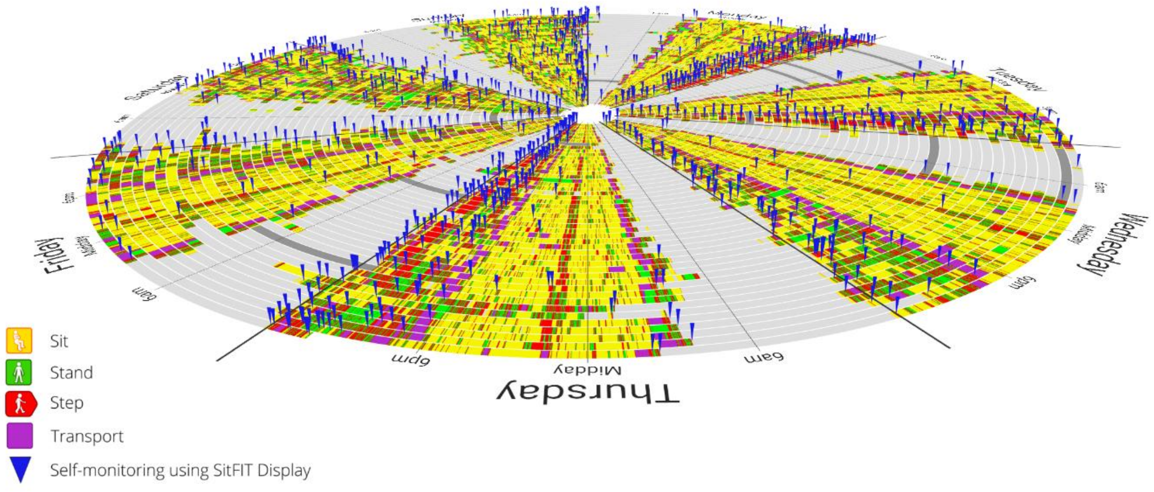

Figure 3. The SitFIT was designed not only to facilitate self-monitoring of Physical Activity and Sedentary Behaviour but also to capture the participant's interaction with the SitFIT, for example wear-time and self-monitoring events, providing the evidence for the effectiveness of the technology component of the intervention.

MatchFIT

The EuroFIT team developed MatchFIT, a web and mobile (iOS and Android) application, to enable participants to view their long-term SitFIT data, to play a team game, and to communicate with each other (Figure S1.3). Participants could only see seven days of data on their SitFIT device, but they could use MatchFIT to view their complete record from the device. This record was displayed aggregated by week, to enable participants to view week-by-week differences. The information for the week could be clicked on to view a day-by-day breakdown. The participant could also enter their own private notes on this page in free text. MatchFIT also contained a team game where the team's collective average step count for a week is compared with a virtual competitor team. To avoid over exertion and risk of injury, the competitor score was determined by a custom algorithm that took account of the team's previous week stepping. To avoid feelings of failure and de-motivation, whether the team won is stated in the app in an ephemeral message that lasts no more than one week. There was no league or permanent record of win or loss. To enhance social support participants could also use MatchFIT to chat with other members of their team. This chat was limited to groups that also meet face-to-face at sessions, to encourage social support within the group and to enhance group cohesion.

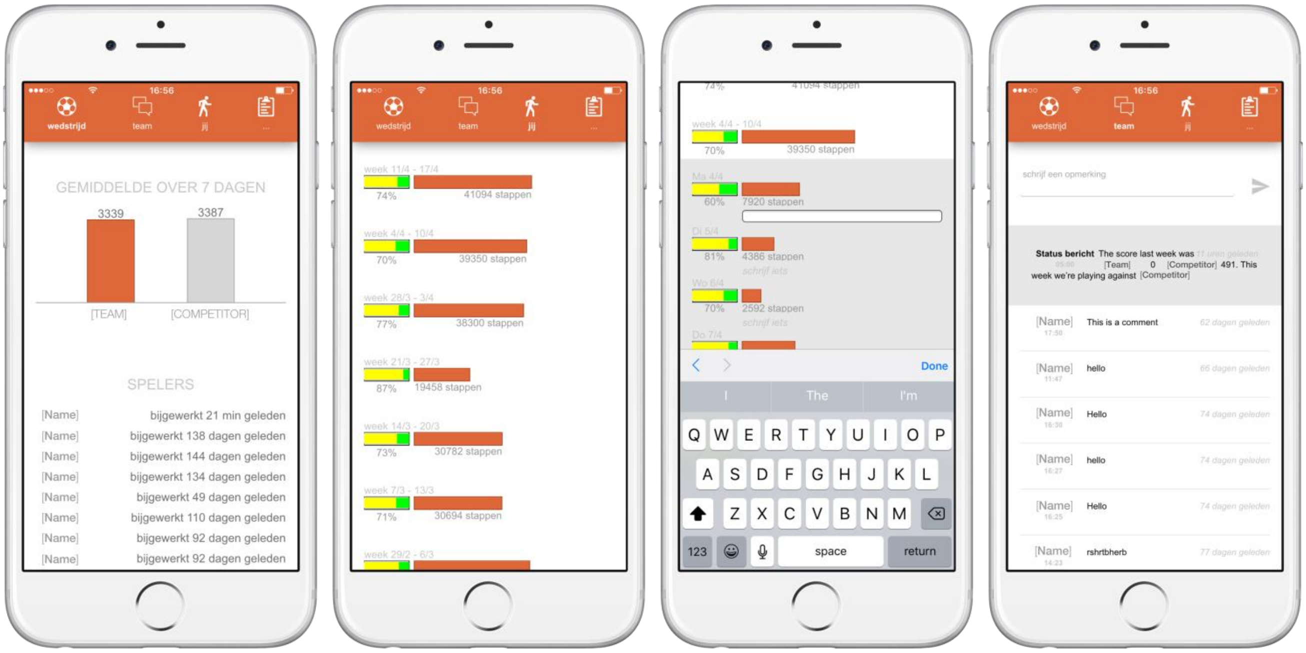

Figure 4. MatchFIT (iOS app version, anonymized screenshots). From left to right (1) The average score of the team is shown against a virtual competitor, (2) A week by week summary of SitFIT data, (3) Commenting on an individual day of data, (4) The team chat function.

Reference

1. Martin A, Adams JM, Bunn C, et al. Feasibility of a real-time self-monitoring device for sitting less and moving more: a randomised controlled trial. *BMJ Open Sport & Exercise Medicine* 2017; **3**(1).
