# Supplementary material for: The effect of a programme to improve men’s sedentary time and physical activity: The European Fans in Training (EuroFIT) randomised controlled trial
Source: PLoS Med. 2019 Feb 5;16(2):e1002736. doi: 10.1371/journal.pmed.1002736 (PMC6363143; doi:10.1371/journal.pmed.1002736)
Supplement: S2 Appendix — (PDF) [file pmed.1002736.s003.pdf]

S2 Appendix. Standard Operating Procedure for preparing activPAL data for analysis using automated sleep and non-wear time algorithm

Version: 4.5 **Date**: 17-10-2017

Follow the procedures to prepare activPAL data for analysis using automated sleep and non-wear time algorithm.

1. Preparation PAL – create PAL Event files with PAL tool

Download the PAL tool (v8.8.9.33) and then follow the instructions below:

Copy the PAL tool into the folder with the datx files (any problematic files including duplicates and files with incorrect dates need to be removed from the final set of datx files prior to using the AOSD algorithm)

Screenshot of where located in our system provided

In the address bar type "cmd" (see below)

Screenshot of where located in our system provided

A Command Line Window will open

Screenshot of where located in our system provided

Run the tool

Type “PALanalysis_cl_v8.8.9.33 -a AOSD -e EVENTS” at the command line prompt.

This will batch process all files in the folder but due to the large amount files the algorithm cannot process these in a single run, the program will stop working after x amount of files processed. Move the processed files and .csv generated to a sub folder and re-run the algorithm. This step should be repeated until all the files have been processed. The AOSD algorithm is the standard PAL algorithm with only the inverted wear correction enabled (i.e. no non-wear or lying algorithm).

Screenshot of what this would look like in our system provided

The events export parameter will output the events.csv files and a log will be saved in the automatic generated ‘Logs’ folder.

Run summary output

Next run Type “PALanalysis_cl_v8.8.9.33 -e DAILY_SUMMARIES_SF” at the command line prompt.

This will generate an excel file with the PAL algorithm, you need this later to identify the first wear day.

Making RAW datafile

The automated sleep and non-wear algorithm developed by Winkler et al. (2016) will be used in STATA 14.1.

Sources:

Link to published article: http://iopscience.iop.org/article/10.1088/0967-3334/37/10/1653?pageTitle=IOPscience

Link to appendices: http://iopscience.iop.org/article/10.1088/0967-3334/37/10/1653/data

instructions below based on workshop Charlotte Edwardson, May 2017 Leicester

Please note that you will complete the steps below separately for each time point (baseline, 3M and 12M). So in total, the whole process will be conducted three times.

STEP 1. RUN STATA code:

Open STATA code "Sleep algorithm.do"

Lines 21-69 contains code that should be changed. Change:

File path (where the event files are located) on line 26. Replace "???" with the file path where the event files are stored. For example "*C:\Eurofit activPAL\baseline\Event files*"

ID number and length on line 43, 44. So please double check the following "Start" = **1** and "Number" = **7**

Make sure you do not have duplicate IDNO files in one folder > this will not run well with the algorithm.

Do not change anything else!

Press save to save the changes.

Click on EXECUTE to run the code.

STEP 2: Create outputs:

Open STATA code "Create variables for analysis algorithm.do"

Change:

File path (where the event files are located) on line 18. Replace "???" with the file path where the event files are stored. For example "*C:\Eurofit activPAL\baseline\Event files*"

Do not change anything else!

Press save to save the changes.

Click on EXECUTE to run the code.

Clean data

The output file (all.csv) will present 1 line of data per participant with all variables presented per day. Below you can find an explanation of the generated variables. The variables of each day are presented first following by the averages across all valid days. The number at the end of each variable represents the number of the day.

Variable	Explanation
id	Subject and visit identifier
Each variable listed below will be generated per valid day. The number indicates what day the data belongs to.	
date1	Date of measurement (day 1)
sed_bouts0_total1	Number of sitting/lying bouts lasting 0-30 mins (for each valid day of data collection) (day 1)
sed_bouts30_total1	Number of sitting/lying bouts lasting 30-60 mins (for each valid day of data collection) (day 1)
sed_bouts60_total1	Number of sitting/lying bouts lasting 60-120 mins (for each valid day of data collection) (day 1)

sed_bouts120_total1	Number of sitting/lying bouts lasting 120+ mins (for each valid day of data collection) (day 1)
sed_bouts_total1	Total number of sitting/lying bouts (for each valid day of data collection) (day 1)
sed_to_upr1	Total number of sit-upright transitions (for each valid day of data collection) (day 1)
total_steps1	Total number of steps for 1 leg (for each valid day of data collection) (day 1)
weartime_total_h1	Total waking wear time in hours (for each valid day of data collection) (day 1)
sed_total_h1	Total sitting/lying time in hours (for each valid day of data collection) (day 1)
step_total_h1	Total stepping time in hours (for each valid day of data collection) (day 1)
light_total_h1	Total light stepping time in hours (for each valid day of data collection) (day 1)
mvpa_total_h1	Total MVPA stepping time in hours (for each valid day of data collection) (day 1)
stand_total_h1	Total standing time in hours (for each valid day of data collection) (day 1)
sed_bout_0_h1	Time in hours spent in sitting/lying bouts lasting 0-30 mins (for each valid day of data collection) (day 1)
sed_bout_30_h1	Time in hours spent in sitting/lying bouts lasting 30-60 mins (for each valid day of data collection) (day 1)
sed_bout_60_h1	Time in hours spent in sitting/lying bouts lasting 60-120 mins (for each valid day of data collection) (day 1)
sed_bout_120_h1	Time in hours spent in sitting/lying bouts lasting 120+ mins (for each valid day of data collection) (day 1)

NOTE 1: day 1 does not always represent day 1 from the diary – follow instructions below.

NOTE 2: do not use the generated averages and number of valid days variable. For EuroFIT we will only use the first seven wear days – follow instructions below.

IDENTIFY FIRST 24H WEAR DAY

The first wear date is the day that is identified by the PAL tool (step 1.5).

Link the RAWdata (step 2.2) file to the summary excel output (step 1.5);

We use a threshold of >10h wear time per day, which the STATA code has already implemented. But in addition, the <u>first wear day</u> of a person will need to be set to invalid if the wear time variable (weartime_total_h) is <u>exceeding 20 hours.</u>

IDENTIFY VALID DAYS:

Only output data files of participants with at least 4 valid days will be included in analyses;

As a lot of men have worn the activPAL during more than 7 days, we need to make sure to use only the first seven days (1st wear week), which starts on the first wear date identified above by the PAL tool (step 1.5).

Check if those first 7 days contain 4 valid days. If the participants has 4 valid days or more, set all days exceeding the 7th day or before day 1 as invalid.

If there are no 4 valid days, set all data as invalid at this time point for this participant.

Compute variables

<u>For each variable calculate the average over the valid days only</u>

**variable**	**label**	**How to construct?**
**OVERALL**		
AvNumSteps	average daily step count based on valid	Use variables: total_steps

	days	**>multiply value by two to obtain steps for 2 legs**<br>>calculate the average over the valid days only
AvSedTime	average daily time spent sitting based on valid days (minutes/day)	Use variables sed_total_h<br>>convert to minutes<br>>calculate the average over the valid days only
NumValid_days	number of valid days	Do not use the automated number of valid days variable, but calculate new variable<br>>add up number of valid days after cleaning process
AvWakeWearTime	average daily wake wear time based on valid days (minutes/day)	Use variables weartime_total_h<br>>convert to minutes<br>>calculate the average over the valid days only
AvStandTime	average daily time spent standing based on valid days (minutes/day)	Use variables stand_total_h<br>>convert to minutes<br>>calculate the average over the valid days only
AvStepTime	average daily time spent stepping based on valid days (minutes/day)	Use variables: step_total_h<br>>convert to minutes<br>>calculate the average over the valid days only
AvUprightTime	average daily time spent upright based on valid days (minutes/day)	> calculate by adding up step_total_h and stand_total_h<br>>convert to minutes<br>>calculate the average over the valid days only
AvNumSitToStandTrans	average daily number of sit-to-stand transitions based on valid days	Use variables sed_to_upr<br>>calculate the average over the valid days only
AvNumSedBouts0	Average daily number of sitting/lying bouts lasting 0-30 mins based on valid days	Use variables sed_bouts0_total<br>>calculate the average over the valid days only
AvNumSedBouts30	Average daily number of sitting/lying bouts lasting 30-60 mins based on valid days	Use variables sed_bouts30_total<br>>calculate the average over the valid days only
AvNumSedBouts60	Average daily number of sitting/lying bouts	Use variables

	lasting 60-120 mins based on valid days	sed_bouts60_total<br>>calculate the average over the valid days only
AvNumSedBouts120	Average daily number of sitting/lying bouts lasting 120+ mins based on valid days	Use variables sed_bouts120_total<br>>calculate the average over the valid days only
AvLightPATime	Average daily time spent in light stepping time in hours based on valid days (minutes/day)	Use variables light_total_h<br>>convert to minutes<br>>calculate the average over the valid days only
AvMVPATime	average daily time spent in moderate/vigorous activity based on valid days (minutes/day)	Use variables mvpa_total_h<br>>convert to minutes<br>>calculate the average over the valid days only
MeetingPAguide	meeting PA recommendation based on valid days (WHO definition)	Use variable AvMVPATime and multiply by 7<br>> dichotomize variable into<br>0-not meeting (<150min per week)<br>1-meeting (equal or more than 150min/week)
AvSedTime10h	average daily sitting time >10 hours/day based on valid days (minutes/day)	Use the average variable (AvSedTime) for his calculation!<br>> calculate by dichotomize variable AvSedTime into<br>0-less than 600 min/day<br>1-equal or more than 600min/day

Calculate all variables and rename the labels
