# Supplementary material for: The effect of a programme to improve men’s sedentary time and physical activity: The European Fans in Training (EuroFIT) randomised controlled trial
Source: PLoS Med. 2019 Feb 5;16(2):e1002736. doi: 10.1371/journal.pmed.1002736 (PMC6363143; doi:10.1371/journal.pmed.1002736)
Supplement: S3 Appendix — EuroFIT, European Fans in Training. (PDF) [file pmed.1002736.s004.pdf]

Participant IDNO: _________

SELF COMPLETE BASELINE QUESTIONNAIRE

Please allow <u>30-45 minutes</u> for this questionnaire completion, which includes questions about your physical activity, eating habits, general physical health and wellbeing.

The questions were developed by different people to measure different aspects of health and wellbeing. This means that the scale for responses is often different. Please look very carefully at the questions to check the scale.

There are no right or wrong answers and no trick questions. We simply want you to provide answers that are most relevant to you. Your responses will be confidential to the research team and only used for research purposes.

Your answers are important to us

DATE

		/			/		

1. Which of the following football clubs do you support, if any?

*(Please tick **ONE** box)*

Arsenal	Everton	Newcastle United	Manchester City	Stoke City	Other
☐ 1	☐ 2	☐ 3	☐ 4	☐ 5	☐ 6

☐ 7 other, please specify: ✎ ... ________________________________

2. Answer the following questions based on your feelings for the team named above.

*(Please tick **ONE** box)*

Question								
a. How important is it to you that your team wins?	Not important ☐ 1	☐ 2	☐ 3	☐ 4	☐ 5	☐ 6	☐ 7	Very important ☐ 8
b. How strongly do you see yourself as a fan of your team?	Not at all a fan ☐ 1	☐ 2	☐ 3	☐ 4	☐ 5	☐ 6	☐ 7	Very much a fan ☐ 8
c. How strongly do your friends see you as a fan of your team?	Not at all a fan ☐ 1	☐ 2	☐ 3	☐ 4	☐ 5	☐ 6	☐ 7	Very much a fan ☐ 8
d. During the season, how closely do you follow your team via ANY of the following: in person or on television, on the radio, or televised news or a newspaper, or website or social media?	Never ☐ 1	☐ 2	☐ 3	☐ 4	☐ 5	☐ 6	☐ 7	Almost every day ☐ 8
e. How important is being a fan of your team to you?	Not important ☐ 1	☐ 2	☐ 3	☐ 4	☐ 5	☐ 6	☐ 7	Very important ☐ 8
f. How much do you dislike the greatest rivals of your team	Do not dislike ☐ 1	☐ 2	☐ 3	☐ 4	☐ 5	☐ 6	☐ 7	Dislike very much ☐ 8
g. How often do you display the above team's name or insignia at your place of work, where you live, or on your clothing?	Never ☐ 1	☐ 2	☐ 3	☐ 4	☐ 5	☐ 6	☐ 7	Always ☐ 8

3. How far away is the home ground of the team you support from your home?
*(Please tick **ONE** box)*

Less than a mile	2-3 miles	4-5 miles	6-10 miles	More than10 miles
☐ 1	☐ 2	☐ 3	☐ 4	☐ 5

4. When you go to your team's home game, how do you usually travel there?
*(Please tick **ONE** box)*

Walk/cycle most or all of the way	Use public transport (bus/tram/metro/train)	Go by car	Do not got to my team's home games
☐ 1	☐ 2	☐ 3	☐ 4

☐ 4 other, please specify: ✎ … ___________________________________

We are interested in finding out about the kinds of physical activities that people do as part of their everyday lives. The questions will ask you about the time you spent being physically active in the last 7 days. Please answer each question even if you do not consider yourself to be an active person. Please think about the activities you do at work, as part of your house and yard work, to get from place to place, and in your spare time for recreation, exercise or sport.

Think about all the **vigorous** activities that you did in the **last 7 days**. **Vigorous** physical activities refer to activities that take hard physical effort and make you breathe much harder than normal. Think *only* about those physical activities that you did for at least 10 minutes at a time.

5. **During the <u>last 7 days</u>, on how many days did you do <u>vigorous</u> physical activities like heavy lifting, digging, aerobics, or fast bicycling?**

✎… _____ **days PER WEEK**

☐₁ No vigorous physical activities ➡ ***Skip to question 7***

6. **How much time did you usually spend doing <u>vigorous</u> physical activities on one of those days?**

✎… ______ **hours PER DAY**

✎… ______ **minutes PER DAY**

☐₁ Don't know/Not sure

Think about all the **moderate** activities that you did in the **last 7 days**. **Moderate** activities refer to activities that take moderate physical effort and make you breathe somewhat harder than normal. Think only about those physical activities that you did for at least 10 minutes at a time.

7. **During the <u>last 7 days</u>, on how many days did you do <u>moderate</u> physical activities like carrying light loads, bicycling at a regular pace, or doubles tennis? Do not include walking.**

✎… ______ **days PER WEEK**

☐₁ No moderate physical activities ➡ ***Skip to question 9***

8. **How much time did you usually spend doing <u>moderate</u> physical activities on one of those days?**

✎... _____ **hours PER DAY**

✎... _____ **minutes PER DAY**

☐₁ Don't know/Not sure

Think about the time you spent **walking** in the **last 7 days**. This includes at work and at home, walking to travel from place to place, and any other walking that you might do solely for recreation, sport, exercise, or leisure.

9. **During the <u>last 7 days</u>, on how many days did you <u>walk</u> for at least 10 minutes at a time?**

✎... _____ **days PER WEEK**

☐₁ No walking ➡ ***Skip to question 11***

10. **How much time did you usually spend <u>walking</u> on one of those days?**

✎... _____ **hours PER DAY**

✎... _____ **minutes PER DAY**

☐₁ Don't know/Not sure

The next question asks you about the time you spend sitting.

(Please write your answers in the spaces provided)

During the last 7 days, how much time did you spent sitting in the following situations on a **usual week day** and **a usual weekend day**:

	WEEK day Hours	WEEK day Minutes	WEEKEND day Hours	WEEKEND day Minutes
**11. For TRANSPORT** (e.g. in car, bus, train, etc.)	✎… Per day	✎… Per day	✎… Per day	✎… Per day
**12. At WORK** (e.g. sitting at a desk or using a computer)	✎… Per day	✎… Per day	✎… Per day	✎… Per day
**13. Watching TV**	✎… Per day	✎… Per day	✎… Per day	✎… Per day
**14. Using a computer at home** (e.g. email, games, information, chatting)	✎… Per day	✎… Per day	✎… Per day	✎… Per day
**15. Other leisure activities** (e.g. socialising, movies etc., but NOT including TV or computer use)	✎… Per day	✎… Per day	✎… Per day	✎… Per day

(Please put "0" if you do not spend any time doing it)

About how many hours in each 24 hour day do you usually spend doing the following:

16. Sleeping (including at night and naps)

✎… ________ hours PER DAY

17. Standing

✎… ________ hours PER DAY

18. Please indicate how often you have engaged in the following activities <u>in the LAST MONTH</u>:

*(Please tick **ONE** box on **EACH** line)*	Never	Occasionally	Sometimes	Frequently	Always
**a. Used stairs instead of escalators or lifts** *(example: in your apartment block, at a shopping mall, at work, etc.)*	☐1	☐2	☐3	☐4	☐5
**b. Walked instead of driving or taking public transport** *(example: to go shopping, when you are out and about in your local area, to the post office or pharmacy, etc.)*	☐1	☐2	☐3	☐4	☐5
**c. Parked away from destination or got off public transport early to have a longer walk** *(example: in a shopping mall, store, cinema, work, etc.)*	☐1	☐2	☐3	☐4	☐5
**d. Used work breaks to be physically active** *(example: walk during lunch break, walk to deliver a message to a colleague instead of using email or telephone, choose a bathroom further away from your office room, etc.)*	☐1	☐2	☐3	☐4	☐5
	☐6 not applicable				
**e. Chosen to stand up instead of sitting** *(example: at home, while on the telephone, while waiting in a public place, while waiting for transportation, etc.)*	☐1	☐2	☐3	☐4	☐5
**f. Chosen to do things by hand instead of using mechanical/automatic tools** *(example: washing your car or windows)*	☐1	☐2	☐3	☐4	☐5

The next questions looks at what you may have **eaten** over the last 7 days. Please read each question carefully, ticking the appropriate box for each option.

19. About how many times over the LAST 7 DAYS did you eat breakfast?

*(Please tick **ONE** box)*

☐1 No times

☐2 1-2 times

☐3 3-5 times

☐4 6 or more times

20. About how many times over the LAST 7 DAYS did you eat a serving of the following?

*(Please tick **ONE** box on **EACH** line)*

	No times	1-2 times	3-5 times	6 or more times
a. **Cheese** *(any except low fat soft cheese such as cottage cheese)*	☐1	☐2	☐3	☐4
b. **Burgers or sausages**	☐1	☐2	☐3	☐4
c. **Beef, Pork or Lamb** *(e.g. roast, mince, steak, stews etc.)*	☐1	☐2	☐3	☐4
d. **Fried food** *(e.g. fried fish, fried chicken, fried eggs)*	☐1	☐2	☐3	☐4
e. **Chips or French fries**	☐1	☐2	☐3	☐4
f. **Bacon, ham, pate, etc.**	☐1	☐2	☐3	☐4
g. **Savoury pies, pasties, sausage rolls, pork pies, etc.**	☐1	☐2	☐3	☐4
h. **Savoury snacks** *(e.g. crisps, twiglets, tortilla chips, etc)*	☐1	☐2	☐3	☐4

21. Are you vegetarian?

☐1 Yes

☐2 No

22. Thinking about the <u>LAST 7 DAYS</u>: about <u>how many times</u> A DAY did you eat or drink the following:

*(Please tick **ONE** box on **EACH** line)*

→PLEASE REPORT THE AMOUNT OF TIMES PER DAY

	Less than once a day	1-2 times a day	3-5 times a day	6 or more times a day
**a. Fruit**	☐1	☐2	☐3	☐4
**b. Vegetables** *(not potatoes)*	☐1	☐2	☐3	☐4
**c. Chocolate, sweets**	☐1	☐2	☐3	☐4
**d. Biscuits**	☐1	☐2	☐3	☐4
**e. Sugary drinks** *(fizzy drinks, diluting/ fruit juice)*	☐1	☐2	☐3	☐4

23. Thinking about the LAST 7 DAYS: about <u>how much milk</u> did you use in A DAY, for drinking or in cereal, tea or coffee?

*(Please tick **ONE** box)*	Less than a quarter pint	About a quarter pint	About half a pint	1 pint or more
	☐1	☐2	☐3	☐4

☐0 **I do not drink milk**

24. What <u>kind of milk</u> do you usually use?

*(Please tick **ONE** box)*	Full cream (blue top)	Semi skimmed (green top)	Skimmed (red top)
	☐1	☐2	☐3

☐0 **I do not drink milk**

25. About how many <u>alcoholic drinks</u> do you have each week?
one drink = a glass of wine, half pint of beer or single measure of spirits

(Please put "0" if you do not drink, or have less than one drink each week)

✐… ________ number of alcoholic drinks each week

26. On <u>how many days</u> each week do you usually drink alcohol?

✐… ________ days each week

27. Have you <u>ever</u> smoked?
*(Please tick **ONE** box)*

☐ 1 No, I've never smoked

☐ 2 Yes, but I am an ex-smoker ➘
When did you give up? ✐… ________________ **(MONTH)** ✐… ________ **(YEAR)**

☐ 3 Yes, I smoke now ➘
How many do you usually smoke per day? ✐… ________ cigarettes/cigars/
other, please specify: ✐… ________________________________

The ladder below depicts life satisfaction.

28. How good is your life when you step back and think about it?

The 10 at the top represents the best possible life for you, with lower numbers indicating lesser degrees of fulfilment. On which step of the ladder do you feel you stand now?

*(Please tick **ONE** box)*

THE BEST POSSIBLE LIFE

☐ 10
☐ 9
☐ 8
☐ 7
☐ 6
☐ 5
☐ 4
☐ 3
☐ 2
☐ 1

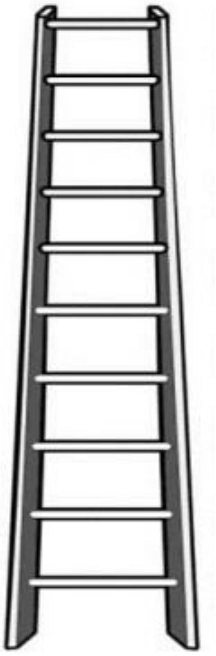

THE LEAST POSSIBLE LIFE

29. Please tick one box on each line below to show whether you strongly agree, agree, disagree or strongly disagree with each statement....

*(Please tick **ONE** box on **EACH** line)*

		Strongly Agree	Agree	Disagree	Strongly Disagree
A	On the whole, I am satisfied with myself.	☐ 1	☐ 2	☐ 3	☐ 4
B	At times, I think I am no good at all.	☐ 1	☐ 2	☐ 3	☐ 4
C	I feel that I have a number of good qualities.	☐ 1	☐ 2	☐ 3	☐ 4
D	I am able to do things as well as most other people.	☐ 1	☐ 2	☐ 3	☐ 4
E	I feel I do not have much to be proud of.	☐ 1	☐ 2	☐ 3	☐ 4

		Strongly Agree	Agree	Disagree	Strongly Disagree
F	I certainly feel useless at times.	☐ 1	☐ 2	☐ 3	☐ 4
G	I feel that I'm a person of worth, at least on an equal plane with others.	☐ 1	☐ 2	☐ 3	☐ 4
H	I wish I could have more respect for myself.	☐ 1	☐ 2	☐ 3	☐ 4
I	All in all, I am inclined to feel that I am a failure.	☐ 1	☐ 2	☐ 3	☐ 4
J	I take a positive attitude toward myself.	☐ 1	☐ 2	☐ 3	☐ 4

30. The following items ask about how you have felt during the PAST MONTH.

Please tick one box on each line below to show whether each statement is not at all true or very true for you in general in your life on a 7-point scale.

*(Please tick **ONE** box on **EACH** line)*

	Not at all true for me			Somewhat true for me			Very true for me
a. I felt alive and vital.	☐ 1	☐ 2	☐ 3	☐ 4	☐ 5	☐ 6	☐ 7
b. I had energy and spirit.	☐ 1	☐ 2	☐ 3	☐ 4	☐ 5	☐ 6	☐ 7
c. I nearly always felt alert and awake.	☐ 1	☐ 2	☐ 3	☐ 4	☐ 5	☐ 6	☐ 7
d. I felt energized.	☐ 1	☐ 2	☐ 3	☐ 4	☐ 5	☐ 6	☐ 7

Under each of the 5 headings below, please tick the one box that best describes **your health TODAY.**

31. Mobility

I have no problems in walking about	☐ 1
I have slight problems in walking about	☐ 2
I have moderate problems in walking about	☐ 3
I have severe problems in walking about	☐ 4
I am unable to walk about	☐ 5

32. Self-Care

I have no problems washing or dressing myself	☐ 1
I have slight problems washing or dressing myself	☐ 2
I have moderate problems washing or dressing myself	☐ 3
I have severe problems washing or dressing myself	☐ 4
I am unable to wash or dress myself	☐ 5

33. Usual Activities (e.g. work, study, housework, family or leisure activities)

I have no problems doing my usual activities	☐ 1
I have slight problems doing my usual activities	☐ 2
I have moderate problems doing my usual activities	☐ 3
I have severe problems doing my usual activities	☐ 4
I am unable to do my usual activities	☐ 5

34. Pain/Discomfort

I have no pain or discomfort	☐ 1
I have slight pain or discomfort	☐ 2
I have moderate pain or discomfort	☐ 3
I have severe pain or discomfort	☐ 4
I have extreme pain or discomfort	☐ 5

35. Anxiety/Depression

I am not anxious or depressed	☐ 1
I am slightly anxious or depressed	☐ 2
I am moderately anxious or depressed	☐ 3
I am severely anxious or depressed	☐ 4
I am extremely anxious or depressed	☐ 5

36. We would like to know how good or bad your health is today.

- This scale on the right is numbered from 0 to 100.
- 100 means the best health you can imagine.
  0 means the worst health you can imagine.
- Mark an X on the scale to indicate how your health is today.
- Now, please write the number you marked on the scale in the box below.

Your Health Today =

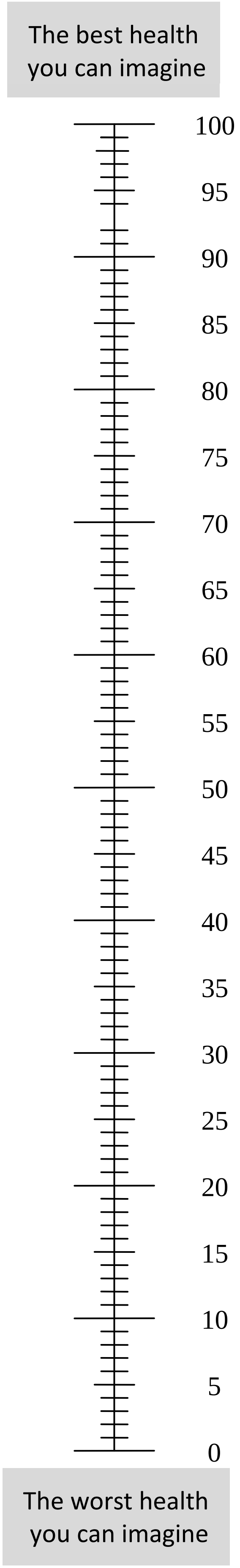

In the past 3 months, did you use any of the medications listed below on prescription from a doctor?

Please write down the name of the medication and the number of days you used this medication.
(Please write your answers in the spaces provided)

37. Cardiovascular medication (such as diuretics, ACE inhibitors, blood pressure lowering drugs, cholesterol inhibitors)

☐0 I did not use cardiovascular medication

	Name	Number of days
✎…	✎…	
✎…	✎…	
✎…	✎…	

38. Painkillers

☐0 I did not use painkillers

	Name	Number of days
✎…		
✎…		
✎…		

39. Inhalers

☐0 I did not use inhalers

	Name	Number of days
✎…		
✎…		
✎…		

40. Antidepressants

☐₀ I did not use antidepressants

	Name	Number of days
✎…		
✎…		
✎…		

41. Other drugs

☐₀ I did not use other drugs

	Name	Number of days
✎…		
✎…		
✎…		

The next section asks you for a few details about you and your current circumstances.

42. What is your date of birth

✎… ________ / ________ / ________ dd/mm/yyyy

43. What is your country of birth?

✎… ____

44. What is the country of birth of your mother?

✎… ____

45. What is the country of birth of your father?

✎… ____________________________________

46. What best describes your current situation?

(Please put a cross in the most appropriate box)

☐ 1 single

☐ 2 married

☐ 3 co-habiting /living with a partner

☐ 4 separated

☐ 5 widowed

☐ 6 divorced

☐ 7 other, please specify: ✏... ___________________________________

47. How many years of formal education since the age of 5 did you have?

With formal education we mean education such as school or intermediate certificate (e.g. GCSE), higher school or leaving certificate (e.g. a level or equivalent), trade/apprenticeship (e.g. hairdresser, chef), certificate/diploma (e.g. technician), university degree or higher (e.g. BA/MSc/PhD), etc.

(Please put a cross in the box that fits best)

☐ 1 less than 7 years of education

☐ 2 7–9 years of education

☐ 3 10–11 years of education

☐ 4 12–13 years of education

☐ 5 14-15 years of education

☐ 6 16-17 years of education

☐ 7 18 years of education or more

☐ 8 other, please specify: ✏... ___________________________________

48. What is your employment status?

(You can put a cross more than one boxes)

☐1 Working full time, please specify your job: ✎... ___________________________________

☐2 Working part-time, please specify your job: ✎... ___________________________________

☐3 Retired

☐4 Caring for the home

☐5 Student

☐6 Not working (but not retired)

☐7 Permanently unable to work/ill

☐8 Other, please specify: ✎... ___________________________________

49. What is the net income of your household PER MONTH, from all sources?

(please include benefits, pensions, superannuation, etc.)

☐1 £ 1,100 or less

☐2 £ 1,100 - 1,700

☐3 £ 1,700 - 2,500

☐4 £ 2,500 - 3,600

☐5 £ 3,600 or more

☐6 Don’t know

☐7 I would rather not answer this question

---

50. People have different reasons why they participate in exercise or do physical activity. If you were to be involved in exercise or do physical activity, please rate the extent to which each statement below is true for you.

*(Please tick **ONE** box on **EACH** line)*

	**Not true for me**		**Sometimes true for me**		**Very true for me**
a. I exercise because other people say I should	☐1	☐2	☐3	☐4	☐5
b. I feel guilty when I don’t exercise	☐1	☐2	☐3	☐4	☐5
c. I value the benefits of exercise	☐1	☐2	☐3	☐4	☐5

	Not true for me		Sometimes true for me		Very true for me
d. I exercise because it's fun	☐ 1	☐ 2	☐ 3	☐ 4	☐ 5
e. I feel ashamed when I miss an exercise session	☐ 1	☐ 2	☐ 3	☐ 4	☐ 5
f. It's important to me to exercise regularly	☐ 1	☐ 2	☐ 3	☐ 4	☐ 5

	Not true for me		Sometimes true for me		Very true for me
g. I can't see why I should bother exercising	☐ 1	☐ 2	☐ 3	☐ 4	☐ 5
h. I enjoy my exercise sessions	☐ 1	☐ 2	☐ 3	☐ 4	☐ 5
i. I exercise because others will not be pleased with me if I don't	☐ 1	☐ 2	☐ 3	☐ 4	☐ 5

	Not true for me		Sometimes true for me		Very true for me
j. I don't see the point in exercising	☐ 1	☐ 2	☐ 3	☐ 4	☐ 5
k. I feel like a failure when I haven't exercised in a while	☐ 1	☐ 2	☐ 3	☐ 4	☐ 5
l. I feel under pressure from my friends/family to exercise	☐ 1	☐ 2	☐ 3	☐ 4	☐ 5

	Not true for me		Sometimes true for me		Very true for me
m. I get pleasure and satisfaction from participating in exercise	☐ 1	☐ 2	☐ 3	☐ 4	☐ 5
n. I think exercising is a waste of time	☐ 1	☐ 2	☐ 3	☐ 4	☐ 5
o. Being physically active is an important part of who I am	☐ 1	☐ 2	☐ 3	☐ 4	☐ 5

51. What does 'success' in being physically activity mean to you?
There are no right or wrong answers.

*(Please tick **ONE** box on **EACH** line)*

When doing physical activity, I feel most successful when…

	Strongly agree		Neutral		Strongly disagree
1. I show other people I am the best	☐ 1	☐ 2	☐ 3	☐ 4	☐ 5
2. I reach personal goals	☐ 1	☐ 2	☐ 3	☐ 4	☐ 5
3. I try hard	☐ 1	☐ 2	☐ 3	☐ 4	☐ 5
4. I beat other people	☐ 1	☐ 2	☐ 3	☐ 4	☐ 5
5. I show clear personal improvement	☐ 1	☐ 2	☐ 3	☐ 4	☐ 5
6. I am the best	☐ 1	☐ 2	☐ 3	☐ 4	☐ 5

52. To what extent do you use the following strategies <u>in order to manage your weight?</u>

*(Please tick **ONE** box on **EACH** line)*

	Never	Rarely	Some times	Frequently	Always
**a. Eating breakfast on a daily basis**	☐ 1	☐ 2	☐ 3	☐ 4	☐ 5
**b. Limiting quantity** *(reducing food portions)*	☐ 1	☐ 2	☐ 3	☐ 4	☐ 5
**c. Restrict intake of certain types of food** *(such as. fats, sugars)*	☐ 1	☐ 2	☐ 3	☐ 4	☐ 5
**d. Drinking fewer sugary drinks**	☐ 1	☐ 2	☐ 3	☐ 4	☐ 5
**e. Drinking less alcohol**	☐ 1	☐ 2	☐ 3	☐ 4	☐ 5
**f. Consciously eating more slowly**	☐ 1	☐ 2	☐ 3	☐ 4	☐ 5

53 How much have you done any of the following over the last 3 months?

*(Please tick **ONE** box on **EACH** line)*

		Not at all	1-2 times a month	About weekly	Every day or most days
a	tried to limit what you eat or drink to try to lose weight?	☐ 1	☐ 2	☐ 3	☐ 4
b	done an exercise workout (including video/DVD workouts) at home?	☐ 1	☐ 2	☐ 3	☐ 4
c	attended a commercial weight loss programme (e.g. Weight Watchers)	☐ 1	☐ 2	☐ 3	☐ 4
d	attended a gym, leisure centre or local sport facility to swim or take part in other physical activity sessions?	☐ 1	☐ 2	☐ 3	☐ 4
e	attended a weight-reduction clinic at your GP surgery or another NHS setting?	☐ 1	☐ 2	☐ 3	☐ 4

54 Is there anything else you have done over the last 3 months to be more physically active, improve your lifestyle or lose weight?

*(Please tick **ONE** box)*

Yes ☐ 1 If yes, please specify: ✎... ______________________

No ☐ 2

55.How did you hear about the programme?

(You can put a cross more than one boxes)

- ☐ 1 club website
- ☐ 2 email from club
- ☐ 3 fans' websites
- ☐ 4 social media (such as Twitter/Facebook)
- ☐ 5 posters at the clubs
- ☐ 6 match day recruitment
- ☐ 7 word of mouth
- ☐ 8 articles or news pieces in the media (newspaper, Radio and TV)
- ☐ 9 work-based advertisement
- ☐ 10 other, please specify: ✎... ______________________

56.What was your reason for joining the programme?

(You can put a cross more than one boxes)

To get fitter	☐	
To lose weight	☐	
To sit less	☐	
To get fitter for a specific reason	☐	What reason? (e.g. to do more with children) ___
To lose weight for a specific reason	☐	What reason? (e.g. family wedding) ✎ … ___
To improve lifestyle	☐	
Health reasons	☐	What health reasons? (personal or family history) ✎ … ___
Someone recommended it to me	☐	Who? (e.g. former participant) ✎ … ___
Someone told me I needed to go on it	☐	Who? (e.g. wife) ✎ … ___
Because it was at the club	☐	
Because it was with men like me	☐	
Other		Please specify ✎ … ___

57. Do you have any formal role in the club, such as being a steward or being employed in the club?

(You can put a cross more than one boxes)

☐ 1 No

☐ 2 Yes, I am a steward at my club

☐ 3 Yes, I am employed by the club

☐ 4 Yes, I have another role (please tell us what this is)

✎ … ___

(YOU ARE FINISHED)
