# Supplementary material for: The effect of a programme to improve men’s sedentary time and physical activity: The European Fans in Training (EuroFIT) randomised controlled trial
Source: PLoS Med. 2019 Feb 5;16(2):e1002736. doi: 10.1371/journal.pmed.1002736 (PMC6363143; doi:10.1371/journal.pmed.1002736)
Supplement: S4 Appendix — EuroFIT, European Fans in Training. (PDF) [file pmed.1002736.s005.pdf]

Participant IDNO: _________

SELF COMPLETE
POST PROGRAMME
QUESTIONNAIRE

Please allow 30-45 minutes for this questionnaire completion, which includes questions about your physical activity, eating habits, general physical health and wellbeing.

The questions were developed by different people to measure different aspects of health and wellbeing. This means that the scale for responses is often different. Please look very carefully at the questions to check the scale.

There are no right or wrong answers and no trick questions. We simply want you to provide answers that are most relevant to you. Your responses will be confidential to the research team and only used for research purposes.

Your answers are important to us

DATE

		/			/		

1. Which of the following football clubs do you support, if any?

*(Please tick **ONE** box)*

Arsenal	Everton	Newcastle United	Manchester City	Stoke City	Other
☐ 1	☐ 2	☐ 3	☐ 4	☐ 5	☐ 6

☐ 7 other, please specify: ✎ … ___________________________________

2. Answer the following questions based on your feelings for the team named above.

*(Please tick **ONE** box)*

a. How important is it to you that your team wins?	Not important ☐ 1	☐ 2	☐ 3	☐ 4	☐ 5	☐ 6	☐ 7	Very important ☐ 8
b. How strongly do you see yourself as a fan of your team?	Not at all a fan ☐ 1	☐ 2	☐ 3	☐ 4	☐ 5	☐ 6	☐ 7	Very much a fan ☐ 8
c. How strongly do your friends see you as a fan of your team?	Not at all a fan ☐ 1	☐ 2	☐ 3	☐ 4	☐ 5	☐ 6	☐ 7	Very much a fan ☐ 8
d. During the season, how closely do you follow your team via ANY of the following: in person or on television, on the radio, or televised news or a newspaper, or website or social media?	Never ☐ 1	☐ 2	☐ 3	☐ 4	☐ 5	☐ 6	☐ 7	Almost every day ☐ 8
e. How important is being a fan of your team to you?	Not important ☐ 1	☐ 2	☐ 3	☐ 4	☐ 5	☐ 6	☐ 7	Very important ☐ 8
f. How much do you dislike the greatest rivals of your team	Do not dislike ☐ 1	☐ 2	☐ 3	☐ 4	☐ 5	☐ 6	☐ 7	Dislike very much ☐ 8
g. How often do you display the above team's name or insignia at your place of work, where you live, or on your clothing?	Never ☐ 1	☐ 2	☐ 3	☐ 4	☐ 5	☐ 6	☐ 7	Always ☐ 8

3. How far away is the home ground of the team you support from your home?
*(Please tick **ONE** box)*

Less than a mile	*2-3 miles*	*4-5 miles*	*6-10 miles*	*More than10 miles*
☐ 1	☐ 2	☐ 3	☐ 4	☐ 5

4. When you go to your team's home game, how do you usually travel there?
*(Please tick **ONE** box)*

Walk/cycle most or all of the way	*Use public transport (bus/tram/metro/train)*	*Go by car*	*Do not got to my team's home games*
☐ 1	☐ 2	☐ 3	☐ 4

☐ 4 other, please specify: ✎... ___________________________________

We are interested in finding out about the kinds of physical activities that people do as part of their everyday lives. The questions will ask you about the time you spent being physically active in the last 7 days. Please answer each question even if you do not consider yourself to be an active person. Please think about the activities you do at work, as part of your house and yard work, to get from place to place, and in your spare time for recreation, exercise or sport.

Think about all the **vigorous** activities that you did in the **last 7 days**. **Vigorous** physical activities refer to activities that take hard physical effort and make you breathe much harder than normal. Think *only* about those physical activities that you did for at least 10 minutes at a time.

5. **During the last 7 days, on how many days did you do vigorous physical activities like heavy lifting, digging, aerobics, or fast bicycling?**

✎... _____ **days PER WEEK**

☐₁ No vigorous physical activities → ***Skip to question 7***

6. **How much time did you usually spend doing <u>vigorous</u> physical activities on one of those days?**

✏… _____ **hours PER DAY**

✏… _____ **minutes PER DAY**

☐₁ Don't know/Not sure

Think about all the **moderate** activities that you did in the **last 7 days**. **Moderate** activities refer to activities that take moderate physical effort and make you breathe somewhat harder than normal. Think only about those physical activities that you did for at least 10 minutes at a time.

7. **During the <u>last 7 days</u>, on how many days did you do <u>moderate</u> physical activities like carrying light loads, bicycling at a regular pace, or doubles tennis? Do not include walking.**

✏… _____ **days PER WEEK**

☐₁ No moderate physical activities → ***Skip to question 9***

8. **How much time did you usually spend doing <u>moderate</u> physical activities on one of those days?**

✏… _____ **hours PER DAY**

✏… _____ **minutes PER DAY**

☐₁ Don't know/Not sure

Think about the time you spent **walking** in the **last 7 days**. This includes at work and at home, walking to travel from place to place, and any other walking that you might do solely for recreation, sport, exercise, or leisure.

9. **During the last 7 days, on how many days did you walk for at least 10 minutes at a time?**

✎… _____ **days PER WEEK**

☐₁ No walking ➡ ***Skip to question 11***

10. **How much time did you usually spend walking on one of those days?**

✎… _____ **hours PER DAY**

✎… _____ **minutes PER DAY**

☐₁ Don't know/Not sure

The next question asks you about the time you spend sitting.

(Please write your answers in the spaces provided)

During the last 7 days, how much time did you spent sitting in the following situations on a **usual week day and a usual weekend day**:

	WEEK day Hours	WEEK day Minutes	WEEKEND day Hours	WEEKEND day Minutes
**11. For TRANSPORT** (e.g. in car, bus, train, etc.)	… Per day	… Per day	… Per day	… Per day
**12. At WORK** (e.g. sitting at a desk or using a computer)	… Per day	… Per day	… Per day	… Per day
**13. Watching TV**	… Per day	… Per day	… Per day	… Per day
**14. Using a computer at home** (e.g. email, games, information, chatting)	… Per day	… Per day	… Per day	… Per day
**15. Other leisure activities** (e.g. socialising, movies etc., but NOT including TV or computer use)	… Per day	… Per day	… Per day	… Per day

(Please put "0" if you do not spend any time doing it)

About how many hours in each 24 hour day do you usually spend doing the following:

16. Sleeping (including at night and naps)

… ________ hours PER DAY

17. Standing

… ________ hours PER DAY

18. Please indicate how often you have engaged in the following activities in the LAST MONTH:

*(Please tick **ONE** box on **EACH** line)*	Never	Occasionally	Some-times	Frequently	Always
**a. Used stairs instead of escalators or lifts** *(example: in your apartment block, at a shopping mall, at work, etc.)*	☐1	☐2	☐3	☐4	☐5
**b. Walked instead of driving or taking public transport** *(example: to go shopping, when you are out and about in your local area, to the post office or pharmacy, etc.)*	☐1	☐2	☐3	☐4	☐5
**c. Parked away from destination or got off public transport early to have a longer walk** *(example: in a shopping mall, store, cinema, work, etc.)*	☐1	☐2	☐3	☐4	☐5
**d. Used work breaks to be physically active** *(example: walk during lunch break, walk to deliver a message to a colleague instead of using email or telephone, choose a bathroom further away from your office room, etc.)*	☐1	☐2	☐3	☐4	☐5
	☐6 not applicable				
**e. Chosen to stand up instead of sitting** *(example: at home, while on the telephone, while waiting in a public place, while waiting for transportation, etc.)*	☐1	☐2	☐3	☐4	☐5
**f. Chosen to do things by hand instead of using mechanical/automatic tools** *(example: washing your car or windows)*	☐1	☐2	☐3	☐4	☐5

The next questions looks at what you may have **eaten** over the last 7 days. Please read each question carefully, ticking the appropriate box for each option.

19. About how many times over the LAST 7 DAYS did you eat breakfast?

*(Please tick **ONE** box)*

☐1 No times

☐2 1-2 times

☐3 3-5 times

☐4 6 or more times

20. About how many times over the LAST 7 DAYS did you eat a serving of the following?

*(Please tick **ONE** box on **EACH** line)*

	No times	1-2 times	3-5 times	6 or more times
a. **Cheese** *(any except low fat soft cheese such as cottage cheese)*	☐1	☐2	☐3	☐4
b. **Burgers or sausages**	☐1	☐2	☐3	☐4
c. **Beef, Pork or Lamb** *(e.g. roast, mince, steak, stews etc.)*	☐1	☐2	☐3	☐4
d. **Fried food** *(e.g. fried fish, fried chicken, fried eggs)*	☐1	☐2	☐3	☐4
e. **Chips or French fries**	☐1	☐2	☐3	☐4
f. **Bacon, ham, pate, etc.**	☐1	☐2	☐3	☐4
g. **Savoury pies, pasties, sausage rolls, pork pies, etc.**	☐1	☐2	☐3	☐4
h. **Savoury snacks** *(e.g. crisps, twiglets, tortilla chips, etc)*	☐1	☐2	☐3	☐4

21. Are you vegetarian?

☐1 Yes

☐2 No

22. Thinking about the LAST 7 DAYS: about how many times A DAY did you eat or drink the following:

*(Please tick **ONE** box on **EACH** line)*

→PLEASE REPORT THE AMOUNT OF TIMES PER DAY

	Less than once a day	1-2 times a day	3-5 times a day	6 or more times a day
**a. Fruit**	☐1	☐2	☐3	☐4
**b. Vegetables** *(not potatoes)*	☐1	☐2	☐3	☐4
**c. Chocolate, sweets**	☐1	☐2	☐3	☐4
**d. Biscuits**	☐1	☐2	☐3	☐4
**e. Sugary drinks** *(fizzy drinks, diluting/ fruit juice)*	☐1	☐2	☐3	☐4

23. Thinking about the LAST 7 DAYS: about how much milk did you use in A DAY, for drinking or in cereal, tea or coffee?

*(Please tick **ONE** box)*	Less than a quarter pint	About a quarter pint	About half a pint	1 pint or more
	☐1	☐2	☐3	☐4

☐0 **I do not drink milk**

24. What kind of milk do you usually use?

*(Please tick **ONE** box)*	Full cream (blue top)	Semi skimmed (green top)	Skimmed (red top)
	☐1	☐2	☐3

☐0 **I do not drink milk**

25. About how many alcoholic drinks do you have each week?

one drink = a glass of wine, half pint of beer or single measure of spirits

(Please put "0" if you do not drink, or have less than one drink each week)

✎… ________ number of alcoholic drinks each week

26. On how many days each week do you usually drink alcohol?

✎… ________ days each week

27. Have you ever smoked?

*(Please tick **ONE** box)*

☐1 No, I've never smoked

☐2 Yes, but I am an ex-smoker ➘

When did you give up? ✎… ________________ **(MONTH)** ✎… ________ **(YEAR)**

☐3 Yes, I smoke now ➘

How many do you usually smoke per day? ✎… ________ cigarettes/cigars/

other, please specify: ✎… ________________________________

The ladder below depicts life satisfaction.

28. How good is your life when you step back and think about it?

The 10 at the top represents the best possible life for you, with lower numbers indicating lesser degrees of fulfilment. On which step of the ladder do you feel you stand now?

*(Please tick **ONE** box)*

THE BEST POSSIBLE LIFE

☐ 10
☐ 9
☐ 8
☐ 7
☐ 6
☐ 5
☐ 4
☐ 3
☐ 2
☐ 1

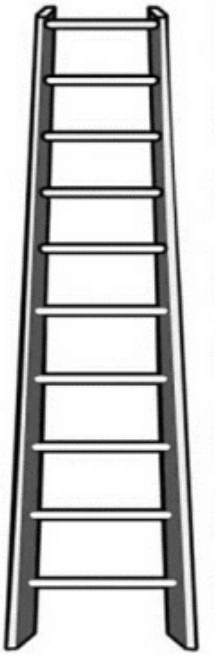

THE LEAST POSSIBLE LIFE

29. Please tick one box on each line below to show whether you strongly agree, agree, disagree or strongly disagree with each statement....

*(Please tick **ONE** box on **EACH** line)*

		Strongly Agree	Agree	Disagree	Strongly Disagree
A	On the whole, I am satisfied with myself.	☐ 1	☐ 2	☐ 3	☐ 4
B	At times, I think I am no good at all.	☐ 1	☐ 2	☐ 3	☐ 4
C	I feel that I have a number of good qualities.	☐ 1	☐ 2	☐ 3	☐ 4
D	I am able to do things as well as most other people.	☐ 1	☐ 2	☐ 3	☐ 4
E	I feel I do not have much to be proud of.	☐ 1	☐ 2	☐ 3	☐ 4

		Strongly Agree	Agree	Disagree	Strongly Disagree
F	I certainly feel useless at times.	☐ 1	☐ 2	☐ 3	☐ 4
G	I feel that I'm a person of worth, at least on an equal plane with others.	☐ 1	☐ 2	☐ 3	☐ 4
H	I wish I could have more respect for myself.	☐ 1	☐ 2	☐ 3	☐ 4
I	All in all, I am inclined to feel that I am a failure.	☐ 1	☐ 2	☐ 3	☐ 4
J	I take a positive attitude toward myself.	☐ 1	☐ 2	☐ 3	☐ 4

30. The following items ask about how you have felt during the PAST MONTH.

Please tick one box on each line below to show whether each statement is not at all true or very true for you in general in your life on a 7-point scale.

*(Please tick **ONE** box on **EACH** line)*

	Not at all true for me			Somewh at true for me			Very true for me
a. I felt alive and vital.	☐ 1	☐ 2	☐ 3	☐ 4	☐ 5	☐ 6	☐ 7
b. I had energy and spirit.	☐ 1	☐ 2	☐ 3	☐ 4	☐ 5	☐ 6	☐ 7
c. I nearly always felt alert and awake.	☐ 1	☐ 2	☐ 3	☐ 4	☐ 5	☐ 6	☐ 7
d. I felt energized.	☐ 1	☐ 2	☐ 3	☐ 4	☐ 5	☐ 6	☐ 7

Under each of the 5 headings below, please tick the one box that best describes **your health TODAY.**

31. Mobility

I have no problems in walking about	☐ 1
I have slight problems in walking about	☐ 2
I have moderate problems in walking about	☐ 3
I have severe problems in walking about	☐ 4
I am unable to walk about	☐ 5

32. Self-Care

I have no problems washing or dressing myself	☐ 1
I have slight problems washing or dressing myself	☐ 2
I have moderate problems washing or dressing myself	☐ 3
I have severe problems washing or dressing myself	☐ 4
I am unable to wash or dress myself	☐ 5

33. Usual Activities (e.g. work, study, housework, family or leisure activities)

I have no problems doing my usual activities	☐	1
I have slight problems doing my usual activities	☐	2
I have moderate problems doing my usual activities	☐	3
I have severe problems doing my usual activities	☐	4
I am unable to do my usual activities	☐	5

34. Pain/Discomfort

I have no pain or discomfort	☐	1
I have slight pain or discomfort	☐	2
I have moderate pain or discomfort	☐	3
I have severe pain or discomfort	☐	4
I have extreme pain or discomfort	☐	5

35. Anxiety/Depression

I am not anxious or depressed	☐	1
I am slightly anxious or depressed	☐	2
I am moderately anxious or depressed	☐	3
I am severely anxious or depressed	☐	4
I am extremely anxious or depressed	☐	5

36. We would like to know how good or bad your health is today.

- This scale on the right is numbered from 0 to 100.
- 100 means the best health you can imagine.
  0 means the worst health you can imagine.
- Mark an X on the scale to indicate how your health is today.
- Now, please write the number you marked on the scale in the box below.

Your Health Today =

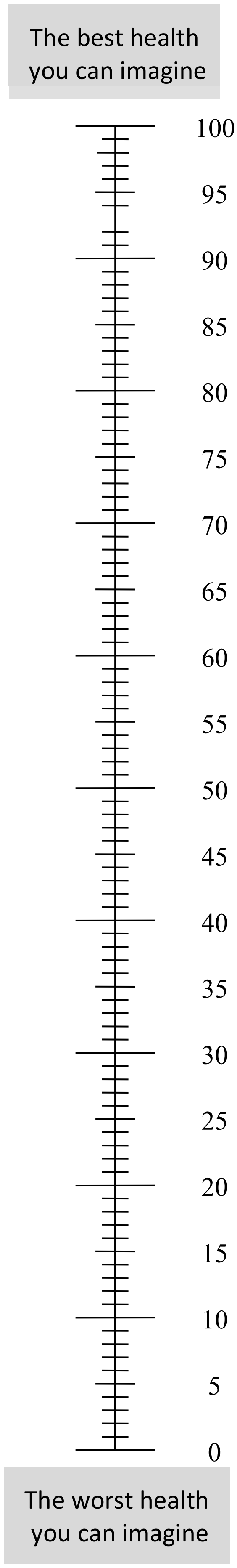

37. People have different reasons why they participate in exercise or do physical activity. If you were to be involved in exercise or do physical activity, please rate the extent to which each statement below is true for you.

*(Please tick **ONE** box on **EACH** line)*

	Not true for me		Sometimes true for me		Very true for me
a. I exercise because other people say I should	☐ 1	☐ 2	☐ 3	☐ 4	☐ 5
b. I feel guilty when I don't exercise	☐ 1	☐ 2	☐ 3	☐ 4	☐ 5
c. I value the benefits of exercise	☐ 1	☐ 2	☐ 3	☐ 4	☐ 5

	Not true for me		Sometimes true for me		Very true for me
d. I exercise because it's fun	☐ 1	☐ 2	☐ 3	☐ 4	☐ 5
e. I feel ashamed when I miss an exercise session	☐ 1	☐ 2	☐ 3	☐ 4	☐ 5
f. It's important to me to exercise regularly	☐ 1	☐ 2	☐ 3	☐ 4	☐ 5

	Not true for me		Sometimes true for me		Very true for me
g. I can't see why I should bother exercising	☐ 1	☐ 2	☐ 3	☐ 4	☐ 5
h. I enjoy my exercise sessions	☐ 1	☐ 2	☐ 3	☐ 4	☐ 5
i. I exercise because others will not be pleased with me if I don't	☐ 1	☐ 2	☐ 3	☐ 4	☐ 5

	Not true for me		Sometimes true for me		Very true for me
j. I don’t see the point in exercising	☐ 1	☐ 2	☐ 3	☐ 4	☐ 5
k. I feel like a failure when I haven’t exercised in a while	☐ 1	☐ 2	☐ 3	☐ 4	☐ 5
l. I feel under pressure from my friends/family to exercise	☐ 1	☐ 2	☐ 3	☐ 4	☐ 5

	Not true for me		Sometimes true for me		Very true for me
m. I get pleasure and satisfaction from participating in exercise	☐ 1	☐ 2	☐ 3	☐ 4	☐ 5
n. I think exercising is a waste of time	☐ 1	☐ 2	☐ 3	☐ 4	☐ 5
o. Being physically active is an important part of who I am	☐ 1	☐ 2	☐ 3	☐ 4	☐ 5

38. To what extent do you use the following strategies <u>in order to manage your weight?</u>

*(Please tick **ONE** box on **EACH** line)*

	Never	Rarely	Some times	Frequently	Always
**a. Eating breakfast on a daily basis**	☐ 1	☐ 2	☐ 3	☐ 4	☐ 5
**b. Limiting quantity** *(reducing food portions)*	☐ 1	☐ 2	☐ 3	☐ 4	☐ 5
**c. Restrict intake of certain types of food** *(such as. fats, sugars)*	☐ 1	☐ 2	☐ 3	☐ 4	☐ 5
**d. Drinking fewer sugary drinks**	☐ 1	☐ 2	☐ 3	☐ 4	☐ 5
**e. Drinking less alcohol**	☐ 1	☐ 2	☐ 3	☐ 4	☐ 5
**f. Consciously eating more slowly**	☐ 1	☐ 2	☐ 3	☐ 4	☐ 5

39 How much have you done any of the following over the <u>last 3 months</u>?

*(Please tick **ONE** box on **EACH** line)*

		Not at all	*1-2 times a month*	*About weekly*	*Every day or most days*
a	tried to limit what you eat or drink to try to lose weight?	☐ 1	☐ 2	☐ 3	☐ 4
b	done an exercise workout (including video/DVD workouts) at home?	☐ 1	☐ 2	☐ 3	☐ 4
c	attended a commercial weight loss programme (e.g. Weight Watchers)	☐ 1	☐ 2	☐ 3	☐ 4
d	attended a gym, leisure centre or local sport facility to swim or take part in other physical activity sessions?	☐ 1	☐ 2	☐ 3	☐ 4
e	attended a weight-reduction clinic at your GP surgery or another NHS setting?	☐ 1	☐ 2	☐ 3	☐ 4

40 Is there anything else you have done over the <u>last 3 months</u> to be more physically active, improve your lifestyle or lose weight?

*(Please tick **ONE** box)*

Yes ☐ 1 If yes, please specify: ✎... ________________________

No ☐ 2

41. In the past 3 months, did you consult the healthcare providers below? Only consultations for your health count. If yes, how many times did you visit the healthcare provider in the past three months?

Please add up all visits to appointments, house calls, telephone consultations and surgeries over the past 3 months.

	Visited?		Number of visits
a. General practitioner	☐ 1 No	☐ 2 Yes	✎ ...
b. Physical therapist	☐ 1 No	☐ 2 Yes	✎ ...
c. Dietician	☐ 1 No	☐ 2 Yes	✎ ...
d. Occupational health doctor	☐ 1 No	☐ 2 Yes	✎ ...
e. Social worker, psychologist or psychiatrist	☐ 1 No	☐ 2 Yes	✎ ...
f. Complementary therapist, such as an acupuncturist, homeopath, or reiki therapist.	☐ 1 No	☐ 2 Yes	✎ ...
f. Outpatient appointment	☐ 1 No	☐ 2 Yes	✎ ...
h. Day treatment at a hospital	☐ 1 No	☐ 2 Yes	✎ ...
i. Inpatient stay	☐ 1 No	☐ 2 Yes	***(If Yes, also answer question 42)***
j. Other healthcare providers	☐ 1 No	☐ 2 Yes	***(If Yes, go to question 43)***<br>***(If No, go to question 44)***

42. In the past 3 months, how many times were you admitted to a hospital for more than one day (that is, one or more nights)?

Please indicate how many days you spent at the Intensive Care Unit (ICU) and a general ward separately.

(Please write your answers in the spaces provided)

	Number of days at ICU	Number of days at a general ward
a ✎…		
b ✎…		
c ✎…		
d ✎…		
e ✎…		

43. In the past 3 months, how many times did you visit another healthcare provider than the ones mentioned in question 41?

Please indicate the type of healthcare provider and the number of visits.

(Please write your answers in the spaces provided)

Type of healthcare provider	Number of visits
a ✎…	
b ✎…	
c ✎…	
d ✎…	
e ✎…	

This part concerns the consequences of health problems for employment in a paid job.

These questions pertain to the period covering the past 3 months.

44. Do you have a paid job?

☐ 1 Yes

☐ 2 No *(If No, go to question 51 for intervention group, comparison group finished)*

45. How many hours per week are you paid to work?

✏… ________ hours per week

46. How many days a week do you work?

✏… ________ days per week

47. Did you have to call in sick because of health problems at any time in the past 3 months?

☐ 1 No *(If No, go to question 51 for intervention group, comparison group finished)*

☐ 2 Yes, I was off work during the full three months. *(If Yes, go to question 51 for intervention group, comparison group finished)*

☐ 3 Yes, I was off work during a limited number of days. *(If Yes, go to question 48)*

48. On which date did you call in sick from work first because of health problems?

✏… ________ / ________ / ________ dd/mm/yyyy *(Go to question 49)*

49. How many times have you had to call in sick because of health problems in the last 3 months?

✎… ________ times

50. How many days did you have call in sick because of health problems in the last 3 months? Please add up all working days you called in sick.

✎… ________ working days

Whilst you were taking part in the EuroFIT program, you might have learnt about different ways to help you to become more active, sit less and eat more healthy.

51.During EuroFIT, to what extent were you encouraged to…
0 (not at all) to 4 (a great deal)

	Not at all				A Great Deal
a. use the **SitFIT** for monitoring your step count and/or upright time	☐ 0	☐ 1	☐ 2	☐ 3	☐ 4
b. set **SMART goals** for behavioural change (e.g. for upright time, step count, nutrition or drinking)	☐ 0	☐ 1	☐ 2	☐ 3	☐ 4
c. discuss strategies to overcome **setbacks**	☐ 0	☐ 1	☐ 2	☐ 3	☐ 4

	Not at all				A Great Deal
d. discuss **personal benefits** of, and barriers to, becoming more active and less sedentary	☐ 0	☐ 1	☐ 2	☐ 3	☐ 4
e. become more aware of **conscious** and **non-conscious** influences on behaviour	☐ 0	☐ 1	☐ 2	☐ 3	☐ 4
f. think about of what **motivates** you to make lifestyle changes	☐ 0	☐ 1	☐ 2	☐ 3	☐ 4

	Not at all				A Great Deal
g. get **support** from your friends and/or family in becoming more active, sitting less and eating a healthier diet	☐ 0	☐ 1	☐ 2	☐ 3	☐ 4
h. **sit less** by breaking up your sitting time and standing more	☐ 0	☐ 1	☐ 2	☐ 3	☐ 4
i. become **more active** by making small changes to your everyday life	☐ 0	☐ 1	☐ 2	☐ 3	☐ 4

	Not at all				A Great Deal
j. exercise with EuroFIT men **during the sessions**	☐ 0	☐ 1	☐ 2	☐ 3	☐ 4
k. exercise with EuroFIT men **in between the sessions**	☐ 0	☐ 1	☐ 2	☐ 3	☐ 4
l. use **MatchFIT**	☐ 0	☐ 1	☐ 2	☐ 3	☐ 4

	Not at all				A Great Deal
m. weigh yourself on a **regular basis**	☐ 0	☐ 1	☐ 2	☐ 3	☐ 4
n. use a **food diary** for monitoring what you ate and drank	☐ 0	☐ 1	☐ 2	☐ 3	☐ 4
o. discuss the **healthy eating plate** to reduce portion sizes and choose more healthier options within each category	☐ 0	☐ 1	☐ 2	☐ 3	☐ 4

	Not at all				A Great Deal
p. discuss **drinking behaviour** to limit your intake of sugary drinks and alcohol	☐ 0	☐ 1	☐ 2	☐ 3	☐ 4
q. read **food labels** to make healthier food choices	☐ 0	☐ 1	☐ 2	☐ 3	☐ 4
r. discuss tips for **eating out**	☐ 0	☐ 1	☐ 2	☐ 3	☐ 4

Whilst you were taking part in the EuroFIT program, you might have learnt about different ways to help you to become more active, sit less and eat more healthy**.**

52. During EuroFIT, to what extent did you find <*listed activities below*> useful for making positive changes in your lifestyles?

0 (not useful at all) to 4 (very useful)

	Not useful at all				Very Useful
a. using the **SitFIT** for monitoring step count and/or upright time	☐ 0	☐ 1	☐ 2	☐ 3	☐ 4
b. setting **SMART goals** for behavioural change (e.g. for upright time, step count, nutrition or drinking)	☐ 0	☐ 1	☐ 2	☐ 3	☐ 4
c. discussing strategies to overcome **setbacks**	☐ 0	☐ 1	☐ 2	☐ 3	☐ 4

	Not useful at all				Very Useful
d. discussing **personal benefits** of, and barriers to, becoming more active and less sedentary	☐ 0	☐ 1	☐ 2	☐ 3	☐ 4
e. becoming more aware of **conscious** and **non-conscious** influences on behaviour	☐ 0	☐ 1	☐ 2	☐ 3	☐ 4
f. thinking about of what **motivates** you to make lifestyle changes	☐ 0	☐ 1	☐ 2	☐ 3	☐ 4

	Not useful at all				Very Useful
g. getting **support** from your friends and/or family in becoming more active, sitting less	☐ 0	☐ 1	☐ 2	☐ 3	☐ 4
h. **sitting less** by break up your sitting time and standing more	☐ 0	☐ 1	☐ 2	☐ 3	☐ 4
i. becoming **more active** by making small changes to your everyday life	☐ 0	☐ 1	☐ 2	☐ 3	☐ 4

	Not useful at all				Very Useful
j. exercising with EuroFIT men **during the sessions**	☐ 0	☐ 1	☐ 2	☐ 3	☐ 4
k. exercising with EuroFIT men **in between the sessions**	☐ 0	☐ 1	☐ 2	☐ 3	☐ 4
l. using **MatchFIT**	☐ 0	☐ 1	☐ 2	☐ 3	☐ 4

	Not useful at all				Very Useful
m. weighing yourself on a **regular basis**	☐ 0	☐ 1	☐ 2	☐ 3	☐ 4
n. using a **food diary** for monitoring what you ate and drunk	☐ 0	☐ 1	☐ 2	☐ 3	☐ 4
o. discussing the **healthy eating plate** to reduce portion sizes and choose more healthier options within each category	☐ 0	☐ 1	☐ 2	☐ 3	☐ 4

	Not useful at all				Very Useful
p. discussing **drinking behaviour** to limit your intake of sugary drinks and alcohol	☐ 0	☐ 1	☐ 2	☐ 3	☐ 4
q. reading **food labels** to make healthier food choices	☐ 0	☐ 1	☐ 2	☐ 3	☐ 4
r. discussing tips for **eating out**	☐ 0	☐ 1	☐ 2	☐ 3	☐ 4

53. The EuroFIT programme consisted of 12 sessions. How many sessions were you able to attend?

✎… ________ Sessions

54. What were your main reasons for not attending sessions that you missed?

✎… ____

☐ 1 Not Applicable

55. On a 1-10 scale, how would you rate the EuroFIT programme overall?

☐	☐	☐	☐	☐	☐	☐	☐	☐	☐
1	2	3	4	5	6	7	8	9	10

56. On a 1-10 scale, how would you rate your coach(es) overall?

☐	☐	☐	☐	☐	☐	☐	☐	☐	☐
1	2	3	4	5	6	7	8	9	10

57. Would you recommend the EuroFIT programme to other men?

☐ 1 Yes

☐ 2 No

58. If the EuroFIT programme was not free, how much would you be willing to pay to do it?

✎… _________________ Great British Pounds

59. If the SitFIT device was not free, how much would you be willing to pay for a SitFIT?

✎... __________________ Great British Pounds

60. Is there anything else you'd like to say about the programme?

✎...

____

____

____

____

____

____

____

____

____

____

____

____

____

____

____

____

61. Please tick one box on each line below to show whether each statement is not at all true or very true for you.

*(Please tick **ONE** box on **EACH** line)*

"The EuroFIT coaches…"

	Not true for me		Sometimes true for me		Very true for me
a. Provided me with choices and options	☐ 1	☐ 2	☐ 3	☐ 4	☐ 5
b. Encouraged me to take my own initiative	☐ 1	☐ 2	☐ 3	☐ 4	☐ 5
c. Considered my personal needs	☐ 1	☐ 2	☐ 3	☐ 4	☐ 5

	Not true for me		Sometimes true for me		Very true for me
d. Gave me good advice	☐ 1	☐ 2	☐ 3	☐ 4	☐ 5
e. Made it clear to me what I needed to do to get results	☐ 1	☐ 2	☐ 3	☐ 4	☐ 5
f. Gave me exercises that were suited to my level	☐ 1	☐ 2	☐ 3	☐ 4	☐ 5
g. Helped me to feel confident about exercising	☐ 1	☐ 2	☐ 3	☐ 4	☐ 5

	Not true for me		Sometimes true for me		Very true for me
h. Made time for me even though they were busy	☐ 1	☐ 2	☐ 3	☐ 4	☐ 5
i. Looked after me well	☐ 1	☐ 2	☐ 3	☐ 4	☐ 5
j. Cared about me	☐ 1	☐ 2	☐ 3	☐ 4	☐ 5

62. Please tick one box on each line below to show whether each statement is not at all true or very true for you.

*(Please tick **ONE** box on **EACH** line)*

"During the EuroFIT sessions,…"

	strongly disagree						strongly agree
a. I felt forced to follow decisions the coaches made for me.	☐ 1	☐ 2	☐ 3	☐ 4	☐ 5	☐ 6	☐ 7
b. I felt under pressure to agree with the training regimen the coaches provided	☐ 1	☐ 2	☐ 3	☐ 4	☐ 5	☐ 6	☐ 7
c. I felt pushed by the coaches to behave in certain ways	☐ 1	☐ 2	☐ 3	☐ 4	☐ 5	☐ 6	☐ 7

	strongly disagree						strongly agree
d. At times during the sessions with the coaches I was made to feel incapable	☐ 1	☐ 2	☐ 3	☐ 4	☐ 5	☐ 6	☐ 7
e. There were times when the coaches said things to me directly that made me feel incompetent	☐ 1	☐ 2	☐ 3	☐ 4	☐ 5	☐ 6	☐ 7
f. At times during the sessions the coaches made me feel inadequate	☐ 1	☐ 2	☐ 3	☐ 4	☐ 5	☐ 6	☐ 7

	strongly disagree						strongly agree
g. I felt the coaches could be dismissive of me	☐ 1	☐ 2	☐ 3	☐ 4	☐ 5	☐ 6	☐ 7
h. I felt the coaches disliked me	☐ 1	☐ 2	☐ 3	☐ 4	☐ 5	☐ 6	☐ 7
i. I felt rejected by the coaches	☐ 1	☐ 2	☐ 3	☐ 4	☐ 5	☐ 6	☐ 7

63. Please tick one box on each line below to show whether each statement is not at all true or very true for you.

*(Please tick **ONE** box on **EACH** line)*

"In the EuroFIT programme, the coach thinks I am successful when…"

	**Not at all true**						**Very true**
a. I can demonstrate I am better than others	☐ 1	☐ 2	☐ 3	☐ 4	☐ 5	☐ 6	☐ 7
b. I improve faster than the others	☐ 1	☐ 2	☐ 3	☐ 4	☐ 5	☐ 6	☐ 7
c. I do not make any mistakes	☐ 1	☐ 2	☐ 3	☐ 4	☐ 5	☐ 6	☐ 7

	**Not at all true**						**Very true**
d. I demonstrate personal improvement	☐ 1	☐ 2	☐ 3	☐ 4	☐ 5	☐ 6	☐ 7
e. I make a real effort	☐ 1	☐ 2	☐ 3	☐ 4	☐ 5	☐ 6	☐ 7
f. I learn new skills	☐ 1	☐ 2	☐ 3	☐ 4	☐ 5	☐ 6	☐ 7

64. Think about how you feel when you are with other members of your EuroFIT group and rate how true each of the statements below is for you.

*(Please tick **ONE** box on **EACH** line)*

	Not at all true						Very true
a. I don’t really feel connected with other people in the group	☐ 1	☐ 2	☐ 3	☐ 4	☐ 5	☐ 6	☐ 7
b. I feel part of the group	☐ 1	☐ 2	☐ 3	☐ 4	☐ 5	☐ 6	☐ 7
c. I don’t really mix with other people in the group	☐ 1	☐ 2	☐ 3	☐ 4	☐ 5	☐ 6	☐ 7
d. I can talk with others in the group about things that really matter to me	☐ 1	☐ 2	☐ 3	☐ 4	☐ 5	☐ 6	☐ 7
e. I often feel alone when I am with the other group members	☐ 1	☐ 2	☐ 3	☐ 4	☐ 5	☐ 6	☐ 7
f. I feel close to some people in the group	☐ 1	☐ 2	☐ 3	☐ 4	☐ 5	☐ 6	☐ 7

THANK YOU FOR COMPLETING THIS QUESTIONNAIRE
