# Supplementary material for: The effect of a programme to improve men’s sedentary time and physical activity: The European Fans in Training (EuroFIT) randomised controlled trial
Source: PLoS Med. 2019 Feb 5;16(2):e1002736. doi: 10.1371/journal.pmed.1002736 (PMC6363143; doi:10.1371/journal.pmed.1002736)
Supplement: S5 Appendix — EuroFIT, European Fans in Training. (PDF) [file pmed.1002736.s006.pdf]

Participant IDNO: _________

SELF COMPLETE
12 MONTH PROGRAMME
QUESTIONNAIRE

Please allow 30-45 minutes for this questionnaire completion, which includes questions about your physical activity, eating habits, general physical health and wellbeing.

The questions were developed by different people to measure different aspects of health and wellbeing. This means that the scale for responses is often different. Please look very carefully at the questions to check the scale.

There are no right or wrong answers and no trick questions. We simply want you to provide answers that are most relevant to you. Your responses will be confidential to the research team and only used for research purposes.

Your answers are important to us

DATE

		/			/		
		/			/		

1. Which of the following football clubs do you support, if any?

*(Please tick **ONE** box)*

Arsenal	Everton	Newcastle United	Manchester City	Stoke City	Other
☐ 1	☐ 2	☐ 3	☐ 4	☐ 5	☐ 6

☐ 7 other, please specify: ✎... ___________________________________

2. How far away is the home ground of the team you support from your home?

*(Please tick **ONE** box)*

Less than a mile	*2-3 miles*	*4-5 miles*	*6-10 miles*	*More than 10 miles*
☐ 1	☐ 2	☐ 3	☐ 4	☐ 5

3. When you go to your team's home game, how do you usually travel there?

*(Please tick **ONE** box)*

Walk/cycle most or all of the way	*Use public transport (bus/ tram/ metro/ train)*	*Go by car*	*Do not got to my team's home games*
☐ 1	☐ 2	☐ 3	☐ 4

☐ 4 other, please specify: ✎... ___________________________________

We are interested in finding out about the kinds of physical activities that people do as part of their everyday lives. The questions will ask you about the time you spent being physically active in the last 7 days. Please answer each question even if you do not consider yourself to be an active person. Please think about the activities you do at work, as part of your house and yard work, to get from place to place, and in your spare time for recreation, exercise or sport.

Think about all the **vigorous** activities that you did in the **last 7 days**. **Vigorous** physical activities refer to activities that take hard physical effort and make you breathe much harder than normal. Think *only* about those physical activities that you did for at least 10 minutes at a time.

4. **During the last 7 days, on how many days did you do vigorous physical activities like heavy lifting, digging, aerobics, or fast bicycling?**

✎ … _____ **days PER WEEK**

☐ 1 No vigorous physical activities → ***Skip to question 6***

5. **How much time did you usually spend doing vigorous physical activities on one of those days?**

✎ … ______ **hours PER DAY**

✎ … ______ **minutes PER DAY**

☐ 1 Don’t know/Not sure

Think about all the **moderate** activities that you did in the **last 7 days**. **Moderate** activities refer to activities that take moderate physical effort and make you breathe somewhat harder than normal. Think only about those physical activities that you did for at least 10 minutes at a time.

6. **During the <u>last 7 days</u>, on how many days did you do <u>moderate</u> physical activities like carrying light loads, bicycling at a regular pace, or doubles tennis? Do not include walking.**

✎... _____ **days PER WEEK**

☐1 No moderate physical activities ➡ ***Skip to question 8***

7. **How much time did you usually spend doing <u>moderate</u> physical activities on one of those days?**

✎... _____ **hours PER DAY**

✎... _____ **minutes PER DAY**

☐1 Don't know/Not sure

Think about the time you spent **walking** in the **last 7 days**. This includes at work and at home, walking to travel from place to place, and any other walking that you might do solely for recreation, sport, exercise, or leisure.

8. **During the <u>last 7 days</u>, on how many days did you <u>walk</u> for at least 10 minutes at a time?**

✎... _____ **days PER WEEK**

☐1 No walking ➡ ***Skip to question 10***

9. **How much time did you usually spend <u>walking</u> on one of those days?**

✎... _____ **hours PER DAY**

✎... _____ **minutes PER DAY**

☐1 Don't know/Not sure

The next question asks you about the time you spend sitting.

(Please write your answers in the spaces provided)

During the last 7 days, how much time did you spent sitting in the following situations on a **usual week day** and **a usual weekend day**:

	WEEK day Hours	WEEK day Minutes	WEEKEND day Hours	WEEKEND day Minutes
**10. For TRANSPORT** (e.g. in car, bus, train, etc.)	… Per day	… Per day	… Per day	… Per day
**11. At WORK** (e.g. sitting at a desk or using a computer)	… Per day	… Per day	… Per day	… Per day
**12. Watching TV**	… Per day	… Per day	… Per day	… Per day
**13. Using a computer at home** (e.g. email, games, information, chatting)	… Per day	… Per day	… Per day	… Per day
**14. Other leisure activities** (e.g. socialising, movies etc., but NOT including TV or computer use)	… Per day	… Per day	… Per day	… Per day

(Please put "0" if you do not spend any time doing it)

About how many hours in each 24 hour day do you usually spend doing the following:

15. Sleeping (including at night and naps)

… ________ hours PER DAY

16. Standing

… ________ hours PER DAY

17. Please indicate how often you have engaged in the following activities <u>in the LAST MONTH</u>:

*(Please tick **ONE** box on **EACH** line)*	Never	Occasionally	Some-times	Frequently	Always
**a. Used stairs instead of escalators or lifts** *(example: in your apartment block, at a shopping mall, at work, etc.)*	☐ 1	☐ 2	☐ 3	☐ 4	☐ 5
**b. Walked instead of driving or taking public transport** *(example: to go shopping, when you are out and about in your local area, to the post office or pharmacy, etc.)*	☐ 1	☐ 2	☐ 3	☐ 4	☐ 5
**c. Parked away from destination or got off public transport early to have a longer walk** *(example: in a shopping mall, store, cinema, work, etc.)*	☐ 1	☐ 2	☐ 3	☐ 4	☐ 5
**d. Used work breaks to be physically active** *(example: walk during lunch break, walk to deliver a message to a colleague instead of using email or telephone, choose a bathroom further away from your office room, etc.)*	☐ 1	☐ 2	☐ 3	☐ 4	☐ 5
	☐ 6 not applicable				
**e. Chosen to stand up instead of sitting** *(example: at home, while on the telephone, while waiting in a public place, while waiting for transportation, etc.)*	☐ 1	☐ 2	☐ 3	☐ 4	☐ 5
**f. Chosen to do things by hand instead of using mechanical/automatic tools** *(example: washing your car or windows)*	☐ 1	☐ 2	☐ 3	☐ 4	☐ 5

The next questions looks at what you may have **eaten** over the last 7 days. Please read each question carefully, ticking the appropriate box for each option.

18. About how many times over the LAST 7 DAYS did you eat breakfast?

*(Please tick **ONE** box)*

☐1 No times

☐2 1-2 times

☐3 3-5 times

☐4 6 or more times

19. About how many times over the LAST 7 DAYS did you eat a serving of the following?

*(Please tick **ONE** box on **EACH** line)*

	No times	1-2 times	3-5 times	6 or more times
**a. Cheese** *(any except low fat soft cheese such as cottage cheese)*	☐1	☐2	☐3	☐4
**b. Burgers or sausages**	☐1	☐2	☐3	☐4
**c. Beef, Pork or Lamb** *(e.g. roast, mince, steak, stews etc.)*	☐1	☐2	☐3	☐4
**d. Fried food** *(e.g. fried fish, fried chicken, fried eggs)*	☐1	☐2	☐3	☐4
**e. Chips or French fries**	☐1	☐2	☐3	☐4
**f. Bacon, ham, pate, etc.**	☐1	☐2	☐3	☐4
**g. Savoury pies, pasties, sausage rolls, pork pies, etc.**	☐1	☐2	☐3	☐4
**h. Savoury snacks** *(e.g. crisps, twiglets, tortilla chips, etc)*	☐1	☐2	☐3	☐4

20. Are you vegetarian?

☐ 1 Yes

☐ 2 No

21. Thinking about the LAST 7 DAYS: about how many times A DAY did you eat or drink the following:

*(Please tick **ONE** box on **EACH** line)*

→PLEASE REPORT THE AMOUNT OF TIMES PER DAY

	Less than once a day	1-2 times a day	3-5 times a day	6 or more times a day
a. **Fruit**	☐ 1	☐ 2	☐ 3	☐ 4
b. **Vegetables** *(not potatoes)*	☐ 1	☐ 2	☐ 3	☐ 4
c. **Chocolate, sweets**	☐ 1	☐ 2	☐ 3	☐ 4
d. **Biscuits**	☐ 1	☐ 2	☐ 3	☐ 4
e. **Sugary drinks** *(fizzy drinks, diluting/ fruit juice)*	☐ 1	☐ 2	☐ 3	☐ 4

22. Thinking about the LAST 7 DAYS: about how much milk did you use in A DAY, for drinking or in cereal, tea or coffee?

*(Please tick **ONE** box)*

Less than a quarter pint	About a quarter pint	About half a pint	1 pint or more
☐ 1	☐ 2	☐ 3	☐ 4

☐ 0 **I do not drink milk**

23. What kind of milk do you usually use?

*(Please tick **ONE** box)*

Full cream (blue top)	Semi skimmed (green top)	Skimmed (red top)
☐ 1	☐ 2	☐ 3

☐ 0 **I do not drink milk**

24. About how many alcoholic drinks do you have each week?
one drink = a glass of wine, half pint of beer or single measure of spirits

(Please put "0" if you do not drink, or have less than one drink each week)

✐… ________ number of alcoholic drinks each week

25. On how many days each week do you usually drink alcohol?

✐… ________ days each week

26. Have you ever smoked?
*(Please tick **ONE** box)*

☐1 No, I've never smoked

☐2 Yes, but I am an ex-smoker ➘

When did you give up? ✐… ________________ **(MONTH)** ✐… ________ **(YEAR)**

☐3 Yes, I smoke now ➘

How many do you usually smoke per day? ✐… ________ cigarettes/cigars/
other, please specify: ✐… ________________________________

The ladder below depicts life satisfaction.

27. How <u>good is your life</u> when you step back and think about it?

The 10 at the top represents the best possible life for you, with lower numbers indicating lesser degrees of fulfilment. On which step of the ladder do you feel you stand now?

*(Please tick **ONE** box)*

THE BEST POSSIBLE LIFE

☐ 10
☐ 9
☐ 8
☐ 7
☐ 6
☐ 5
☐ 4
☐ 3
☐ 2
☐ 1

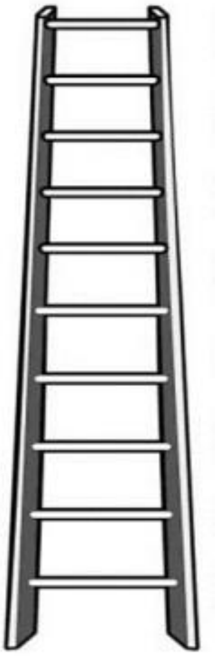

THE LEAST POSSIBLE LIFE

28. Please tick one box on each line below to show whether you strongly agree, agree, disagree or strongly disagree with each statement....

*(Please tick **ONE** box on **EACH** line)*

		Strongly Agree	Agree	Disagree	Strongly Disagree
A	On the whole, I am satisfied with myself.	☐ 1	☐ 2	☐ 3	☐ 4
B	At times, I think I am no good at all.	☐ 1	☐ 2	☐ 3	☐ 4
C	I feel that I have a number of good qualities.	☐ 1	☐ 2	☐ 3	☐ 4
D	I am able to do things as well as most other people.	☐ 1	☐ 2	☐ 3	☐ 4
E	I feel I do not have much to be proud of.	☐ 1	☐ 2	☐ 3	☐ 4

		Strongly Agree	Agree	Disagree	Strongly Disagree
F	I certainly feel useless at times.	☐ 1	☐ 2	☐ 3	☐ 4
G	I feel that I'm a person of worth, at least on an equal plane with others.	☐ 1	☐ 2	☐ 3	☐ 4
H	I wish I could have more respect for myself.	☐ 1	☐ 2	☐ 3	☐ 4
I	All in all, I am inclined to feel that I am a failure.	☐ 1	☐ 2	☐ 3	☐ 4
J	I take a positive attitude toward myself.	☐ 1	☐ 2	☐ 3	☐ 4

29. The following items ask about how you have felt <u>during the PAST MONTH</u>.

Please tick one box on each line below to show whether each statement is not at all true or very true for you in general in your life on a 7-point scale.

*(Please tick **ONE** box on **EACH** line)*

	Not at all true for me			Somewhat true for me			Very true for me
a. I felt alive and vital.	☐ 1	☐ 2	☐ 3	☐ 4	☐ 5	☐ 6	☐ 7
b. I had energy and spirit.	☐ 1	☐ 2	☐ 3	☐ 4	☐ 5	☐ 6	☐ 7
c. I nearly always felt alert and awake.	☐ 1	☐ 2	☐ 3	☐ 4	☐ 5	☐ 6	☐ 7
d. I felt energized.	☐ 1	☐ 2	☐ 3	☐ 4	☐ 5	☐ 6	☐ 7

Under each of the 5 headings below, please tick the one box that best describes **your health <u>TODAY</u>.**

30. Mobility

I have no problems in walking about	☐ 1
I have slight problems in walking about	☐ 2
I have moderate problems in walking about	☐ 3
I have severe problems in walking about	☐ 4
I am unable to walk about	☐ 5

31. Self-Care

I have no problems washing or dressing myself	☐ 1
I have slight problems washing or dressing myself	☐ 2
I have moderate problems washing or dressing myself	☐ 3
I have severe problems washing or dressing myself	☐ 4
I am unable to wash or dress myself	☐ 5

32. Usual Activities (e.g. work, study, housework, family or leisure activities)

I have no problems doing my usual activities	☐ 1
I have slight problems doing my usual activities	☐ 2
I have moderate problems doing my usual activities	☐ 3
I have severe problems doing my usual activities	☐ 4
I am unable to do my usual activities	☐ 5

33. Pain/Discomfort

I have no pain or discomfort	☐ 1
I have slight pain or discomfort	☐ 2
I have moderate pain or discomfort	☐ 3
I have severe pain or discomfort	☐ 4
I have extreme pain or discomfort	☐ 5

34. Anxiety/Depression

I am not anxious or depressed	☐ 1
I am slightly anxious or depressed	☐ 2
I am moderately anxious or depressed	☐ 3
I am severely anxious or depressed	☐ 4
I am extremely anxious or depressed	☐ 5

35. We would like to know how good or bad your health is today.

- This scale on the right is numbered from 0 to 100.
- 100 means the best health you can imagine.
  0 means the worst health you can imagine.
- Mark an X on the scale to indicate how your health is today.
- Now, please write the number you marked on the scale in the box below.

Your Health Today = ☐

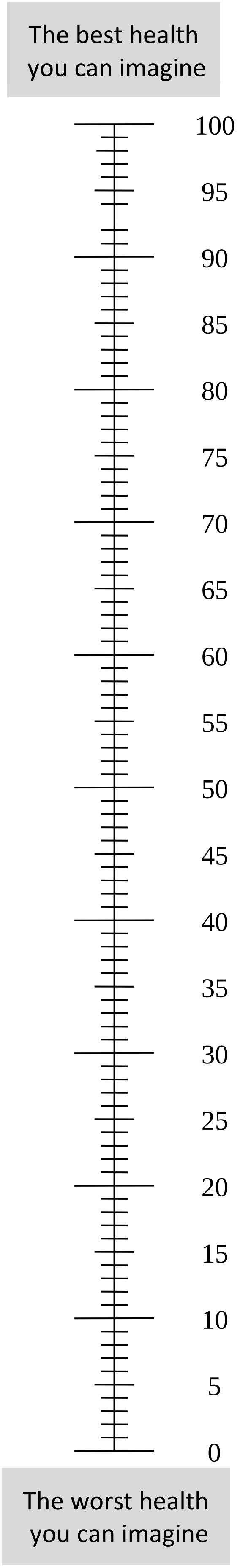

36. People have different reasons why they participate in exercise or do physical activity. If you were to be involved in exercise or do physical activity, please rate the extent to which each statement below is true for you.

*(Please tick **ONE** box on **EACH** line)*

	Not true for me		Sometimes true for me		Very true for me
a. I exercise because other people say I should	☐ 1	☐ 2	☐ 3	☐ 4	☐ 5
b. I feel guilty when I don't exercise	☐ 1	☐ 2	☐ 3	☐ 4	☐ 5
c. I value the benefits of exercise	☐ 1	☐ 2	☐ 3	☐ 4	☐ 5

	Not true for me		Sometimes true for me		Very true for me
d. I exercise because it's fun	☐ 1	☐ 2	☐ 3	☐ 4	☐ 5
e. I feel ashamed when I miss an exercise session	☐ 1	☐ 2	☐ 3	☐ 4	☐ 5
f. It's important to me to exercise regularly	☐ 1	☐ 2	☐ 3	☐ 4	☐ 5

	Not true for me		Sometimes true for me		Very true for me
g. I can't see why I should bother exercising	☐ 1	☐ 2	☐ 3	☐ 4	☐ 5
h. I enjoy my exercise sessions	☐ 1	☐ 2	☐ 3	☐ 4	☐ 5
i. I exercise because others will not be pleased with me if I don't	☐ 1	☐ 2	☐ 3	☐ 4	☐ 5

	Not true for me		Sometimes true for me		Very true for me
j. I don't see the point in exercising	☐ 1	☐ 2	☐ 3	☐ 4	☐ 5
k. I feel like a failure when I haven't exercised in a while	☐ 1	☐ 2	☐ 3	☐ 4	☐ 5
l. I feel under pressure from my friends/family to exercise	☐ 1	☐ 2	☐ 3	☐ 4	☐ 5

	Not true for me		Sometimes true for me		Very true for me
m. I get pleasure and satisfaction from participating in exercise	☐ 1	☐ 2	☐ 3	☐ 4	☐ 5
n. I think exercising is a waste of time	☐ 1	☐ 2	☐ 3	☐ 4	☐ 5
o. Being physically active is an important part of who I am	☐ 1	☐ 2	☐ 3	☐ 4	☐ 5

37. What does 'success' in being physically active mean to you?
There are no right or wrong answers.

(Please tick ***ONE*** *box on* ***EACH*** *line)*

<u>**When doing physical activity, I feel most successful when…**</u>

	Strongly agree		Neutral		Strongly disagree
1. I show other people I am the best	☐ 1	☐ 2	☐ 3	☐ 4	☐ 5
2. I reach personal goals	☐ 1	☐ 2	☐ 3	☐ 4	☐ 5
3. I try hard	☐ 1	☐ 2	☐ 3	☐ 4	☐ 5
4. I beat other people	☐ 1	☐ 2	☐ 3	☐ 4	☐ 5
5. I show clear personal improvement	☐ 1	☐ 2	☐ 3	☐ 4	☐ 5
6. I am the best	☐ 1	☐ 2	☐ 3	☐ 4	☐ 5

38. To what extent do you use the following strategies in order to manage your weight?

*(Please tick **ONE** box on **EACH** line)*

	Never	Rarely	Some times	Frequently	Always
**a. Eating breakfast on a daily basis**	☐ 1	☐ 2	☐ 3	☐ 4	☐ 5
**b. Limiting quantity** *(reducing food portions)*	☐ 1	☐ 2	☐ 3	☐ 4	☐ 5
**c. Restrict intake of certain types of food** *(such as. fats, sugars)*	☐ 1	☐ 2	☐ 3	☐ 4	☐ 5
**d. Drinking fewer sugary drinks**	☐ 1	☐ 2	☐ 3	☐ 4	☐ 5
**e. Drinking less alcohol**	☐ 1	☐ 2	☐ 3	☐ 4	☐ 5
**f. Consciously eating more slowly**	☐ 1	☐ 2	☐ 3	☐ 4	☐ 5

39. How much have you done any of the following over the last 3 months?

*(Please tick **ONE** box on **EACH** line)*		*Not at all*	*1-2 times a month*	*About weekly*	*Every day or most days*
a	tried to limit what you eat or drink to try to lose weight?	☐ 1	☐ 2	☐ 3	☐ 4
b	done an exercise workout (including video/DVD workouts) at home?	☐ 1	☐ 2	☐ 3	☐ 4
c	attended a commercial weight loss programme (e.g. Weight Watchers)	☐ 1	☐ 2	☐ 3	☐ 4
d	attended a gym, leisure centre or local sport facility to swim or take part in other physical activity sessions?	☐ 1	☐ 2	☐ 3	☐ 4
e	attended a weight-reduction clinic at your GP surgery or another NHS setting?	☐ 1	☐ 2	☐ 3	☐ 4

40. Is there anything else you have done over the last 3 months to be more physically active, improve your lifestyle or lose weight?

*(Please tick **ONE** box)*

Yes ☐ 1 If yes, please specify: ✎… ________________________

No ☐ 2

People have different feelings when they engage in physical activity.

41. Using the scale provided, please answer the following questions by considering how you typically feel when participating in physical activity.

*(Please tick **ONE** box on **EACH** line)*

	False	Mostly false	More false than true	More true than false	Mostly true	True
a. I feel capable of completing physical activities that are challenging to me	☐ 1	☐ 2	☐ 3	☐ 4	☐ 5	☐ 6
b. I feel free to choose which physical activities I participate in	☐ 1	☐ 2	☐ 3	☐ 4	☐ 5	☐ 6
c. I feel a sense of camaraderie with my physical activity companions because we do physical activity for the same reason	☐ 1	☐ 2	☐ 3	☐ 4	☐ 5	☐ 6

	False	Mostly false	More false than true	More true than false	Mostly true	True
d. I feel confident in my ability to perform physical activities that personally challenge me	☐ 1	☐ 2	☐ 3	☐ 4	☐ 5	☐ 6
e. I feel like I am the one who decide what physical activities I do	☐ 1	☐ 2	☐ 3	☐ 4	☐ 5	☐ 6
f. I feel connected to the people who I interact with while we do physical activity together	☐ 1	☐ 2	☐ 3	☐ 4	☐ 5	☐ 6

	False	Mostly false	More false than true	More true than false	Mostly true	True
g. I feel good about the way I am able to complete challenging physical activities	☐ 1	☐ 2	☐ 3	☐ 4	☐ 5	☐ 6
h. I feel like I am in charge of my physical activity decisions	☐ 1	☐ 2	☐ 3	☐ 4	☐ 5	☐ 6
i. I feel like I get along well with other people who I interact with while we do physical activity together	☐ 1	☐ 2	☐ 3	☐ 4	☐ 5	☐ 6

42. In the past 3 months, did you consult the healthcare providers below? Only consultations for your health count. If yes, how many times did you visit the healthcare provider in the past three months?

Please add up all visits to appointments, house calls, telephone consultations and surgeries over the past 3 months.

	Visited?		Number of visits
a. General practitioner	☐ 1 No	☐ 2 Yes	✎ ...
b. Physical therapist	☐ 1 No	☐ 2 Yes	✎ ...
c. Dietician	☐ 1 No	☐ 2 Yes	✎ ...
d. Occupational health doctor	☐ 1 No	☐ 2 Yes	✎ ...
e. Social worker, psychologist or psychiatrist	☐ 1 No	☐ 2 Yes	✎ ...
f. Complementary therapist, such as an acupuncturist, homeopath, or reiki therapist.	☐ 1 No	☐ 2 Yes	✎ ...
g. Outpatient appointment	☐ 1 No	☐ 2 Yes	✎ ...
h. Day treatment at a hospital	☐ 1 No	☐ 2 Yes	✎ ...
i. Inpatient stay	☐ 1 No	☐ 2 Yes	***(If Yes, also answer question 43)***
j. Other healthcare providers	☐ 1 No	☐ 2 Yes	***(If Yes, go to question 44)***<br>***(If No, go to question 45)***

43. In the past 3 months, how many times were you admitted to a hospital for more than one day (that is, one or more nights)?

Please indicate how many days you spent at the Intensive Care Unit (ICU) and a general ward separately.

(Please write your answers in the spaces provided)

	Number of days at ICU	Number of days at a general ward
a ✎...		
b ✎...		
c ✎...		
d ✎...		
e ✎...		

44. In the past 3 months, how many times did you visit another healthcare provider than the ones mentioned in question 42?

Please indicate the type of healthcare provider and the number of visits.

(Please write your answers in the spaces provided)

Type of healthcare provider	Number of visits
a ✎...	
b ✎...	
c ✎...	
d ✎...	
e ✎...	

This part concerns the consequences of health problems for employment in a paid job.

These questions pertain to the period covering the past 3 months.

45. Do you have a paid job?

☐1 Yes

☐2 No *(If No, go to question 52)*

46. How many hours per week are you paid to work?

✐... ________ hours per week

47. How many days a week do you work?

✐... ________ days per week

48. Did you have to call in sick because of health problems at any time in the past 3 months?

☐1 No *(If No, go to question 52)*

☐2 Yes, I was off work during the full three months. *(If Yes, go to question 52)*

☐3 Yes, I was off work during a limited number of days. *(If Yes, go to question 49)*

49. On which date did you call in sick from work first because of health problems?

✐... ________ / ________ / ________ dd/mm/yyyy *(Go to question 50)*

50. How many times have you had to call in sick because of health problems in the last 3 months?

✏... ________ times

51. How many days did you have call in sick because of health problems in the last 3 months? Please add up all working days you called in sick.

✏... ________ working days

In the past 3 months, did you use any of the medications listed below on prescription from a doctor?

Please write down the name of the medication and the number of days you used this medication.
(Please write your answers in the spaces provided)

52. Cardiovascular medication (such as diuretics, ACE inhibitors, blood pressure lowering drugs, cholesterol inhibitors)

☐0 I did not use cardiovascular medication

	Name	Number of days
✏...	✏...	
✏...	✏...	
✏...	✏...	

53. Painkillers

☐0 I did not use painkillers

	Name	Number of days
✏...		
✏...		
✏...		

54. Inhalers

☐₀ I did not use inhalers

	Name	Number of days
✎…		
✎…		
✎…		

55. Antidepressants

☐₀ I did not use antidepressants

	Name	Number of days
✎…		
✎…		
✎…		

56. Other drugs

☐₀ I did not use other drugs

	Name	Number of days
✎…		
✎…		
✎…		

COMPARISON GROUP ONLY

57. Have you made any changes to your lifestyle since signing up for EuroFIT?

☐ 1No *(If No, you are finished)*

☐ 2Yes *(If Yes, go to question 58)*

58. If yes, have the changes been influenced by:

**a.** my own general knowledge about how to make changes to my life	☐ 1No	☐ 2Yes
**b.** the information sheet/booklet about being physical active you were given	☐ 1No	☐ 2Yes
**c.** being measured at the EuroFIT measurement session	☐ 1No	☐ 2Yes

59. Please tell us about what changes you have made.

✎…

THANK YOU FOR COMPLETING THIS QUESTIONNAIRE

INTERVENTION GROUP ONLY

Whilst you were taking part in the EuroFIT program, you might have learnt about different ways to help you to become more active, sit less and eat more healthy.

57. How much of each of the following have you done since you completed the EuroFIT programme?

0 (not at all) to 4 (a great deal)	**Not at all**				**A Great Deal**
a. use the **SitFIT** for monitoring your step count and/or upright time	☐ 0	☐ 1	☐ 2	☐ 3	☐ 4
b. set **SMART goals** for behavioural change (e.g. for upright time, step count, nutrition or drinking)	☐ 0	☐ 1	☐ 2	☐ 3	☐ 4
c. discuss strategies to overcome **setbacks**	☐ 0	☐ 1	☐ 2	☐ 3	☐ 4

	**Not at all**				**A Great Deal**
d. discuss **personal benefits** of, and barriers to, becoming more active and less sedentary	☐ 0	☐ 1	☐ 2	☐ 3	☐ 4
e. become more aware of **conscious** and **non-conscious** influences on behaviour	☐ 0	☐ 1	☐ 2	☐ 3	☐ 4
f. think about of what **motivates** you to make lifestyle changes	☐ 0	☐ 1	☐ 2	☐ 3	☐ 4

	**Not at all**				**A Great Deal**
g. get **support** from your friends and/or family in becoming more active, sitting less and eating a healthier diet	☐ 0	☐ 1	☐ 2	☐ 3	☐ 4
h. **sit less** by breaking up your sitting time and standing more	☐ 0	☐ 1	☐ 2	☐ 3	☐ 4
i. become **more active** by making small changes to your everyday life	☐ 0	☐ 1	☐ 2	☐ 3	☐ 4

	**Not at all**				**A Great Deal**
j. use **MatchFIT**	☐ 0	☐ 1	☐ 2	☐ 3	☐ 4
k. weigh yourself on a **regular basis**	☐ 0	☐ 1	☐ 2	☐ 3	☐ 4

l.	use a **food diary** for monitoring what you ate and drank	☐ 0	☐ 1	☐ 2	☐ 3	☐ 4

		Not at all				A Great Deal
m.	discuss **drinking behaviour** to limit your intake of sugary drinks and alcohol	☐ 0	☐ 1	☐ 2	☐ 3	☐ 4
n.	read **food labels** to make healthier food choices	☐ 0	☐ 1	☐ 2	☐ 3	☐ 4
o.	discuss tips for **eating out**	☐ 0	☐ 1	☐ 2	☐ 3	☐ 4

58. How much of each of the following have you done since you completed the EuroFIT programme?

*(Please tick **ONE** box on **EACH** line)*

		Very Frequently	Frequently	Occasionally	Rarely	Never
a.	Been in contact with other men who have taken part in EuroFIT at your club?	☐ 0	☐ 1	☐ 2	☐ 3	☐ 4
b.	Been in contact with the coaches who deliver/have delivered EuroFIT at your club?	☐ 0	☐ 1	☐ 2	☐ 3	☐ 4
c.	Attended any other health or fitness programmes or groups at your club?	☐ 0	☐ 1	☐ 2	☐ 3	☐ 4

59. If you are married, or have a partner, have you noticed them becoming more physically active since you participated in EuroFIT? *(Please tick **ONE** box)*

☐ 1 I do not have a wife/partner *(go to question 62)*

☐ 2 My wife/partner has become more physically active

☐ 3 My wife/partner has NOT become more physically active

60. Have you noticed your wife/partner sitting down less/ standing up more since you participated in EuroFIT?

☐ 1 My wife/partner has sat down less/ stood up more

☐ 2 My wife/ partner has NOT sat down less/ stood up more

61. Have you noticed your wife/partner eating more healthily since you participated in EuroFIT?

☐ 1 My wife/partner has eaten more healthily

☐ 2 My wife/partner has NOT eaten more healthily

---

62. If you have children, or care for children, have you noticed them becoming more physically active since you participated in EuroFIT?

*(Please tick **ONE** box)*

☐ 1 I do not have children or care for children *(go to question 64)*

☐ 2 My children have become more physically active

☐ 3 My children have NOT become more physically active

63. Have you noticed your children eating more healthily since you participated in EuroFIT?

☐ 1 My children have eaten more healthily

☐ 2 My children have NOT eaten more healthily

---

64. If you work or volunteer, have you noticed any of your colleagues becoming more physically active since you participated in EuroFIT?

*(Please tick **ONE** box)*

☐ 1 I do not work or volunteer *(go to question 67)*

☐ 2 Some of my colleagues have become more physically active

☐ 3 None of my colleagues have become more physically active

65. Have you noticed any of your colleagues sitting down less/ standing up more since you participated in EuroFIT?

☐ 1 Some of my colleagues have sat down less/ stood up more

☐ 2 None of my colleagues have sat down less/ stood up more

66. Have you noticed any of your colleagues eating more healthily since you participated in EuroFIT?

☐ 1 Some of my colleagues have eaten more healthily

☐ 2 None of my colleagues have eaten more healthily

67. Have you noticed any of your friends becoming more physically active since you participated in EuroFIT? *(Please tick **ONE** box)*

☐ 1 I do not socialise much *(You are finished with the questionnaire)*

☐ 2 Some of my friends have become more physically active

☐ 3 None of my friends have become more physically active

68. Have you noticed any of your friends sitting down less/ standing up more since you participated in EuroFIT?

☐ 1 Some of my friends have sat down less/ stood up more

☐ 2 None of my friends have sat down less/ stood up more

69. Have you noticed any of your friends eating more healthily since you participated in EuroFIT?

☐ 1 Some of my friends have eaten more healthily

☐ 2 None of my friends have eaten more healthily

THANK YOU FOR COMPLETING THIS QUESTIONNAIRE
