# Supplementary material for: The effect of a programme to improve men’s sedentary time and physical activity: The European Fans in Training (EuroFIT) randomised controlled trial
Source: PLoS Med. 2019 Feb 5;16(2):e1002736. doi: 10.1371/journal.pmed.1002736 (PMC6363143; doi:10.1371/journal.pmed.1002736)
Supplement: S1 Analysis Plan — EuroFIT, European Fans in Training. (PDF) [file pmed.1002736.s007.pdf]

European Fans in Training (EuroFIT): a multi-centre randomised controlled trial of a gender-sensitised programme to increase physical activity and reduce sedentary time delivered to men aged 30-65 in elite European football clubs

EuroFIT

STATISTICAL ANALYSIS PLAN

Study Title:	European Fans in Training (EuroFIT): a multi-centre randomised controlled trial of a gender-sensitised programme to increase physical activity and reduce sedentary time delivered to men aged 30-65 in elite European football clubs
Short Title:	EuroFIT
ISRCTN:	81935608
Sponsor:	University of Glasgow
Funded by:	European Commission FP7 program

Protocol Version:	v10	Date:	24th July 2015
SAP Version:	v1.0	Date:	14th November 2017

		Signature	Date
Prepared by:	Paula McSkimming<br>Trainee Biostatistician<br>Robertson Centre for Biostatistics		16/11/2017
Approved by:	Dr Alex McConnachie<br>Assistant Director of Biostatistics<br>Robertson Centre for Biostatistics		16/11/17
	Professor Sally Wyke<br>Principal Investigator,<br>Institute of Health and Wellbeing		16/11/17
	Dr Christopher Bunn<br>Project Manager,<br>Institute of Health and Wellbeing		14/11/17

CONTENTS

1. Introduction 4
1.1. Study Background 4
1.2. Study Objectives 4
1.3. Study Design 4
1.4. Sample Size and Power 4
1.5. Study Population 5
1.5.1. Inclusion Criteria 5
1.5.2. Exclusion Criteria 5
1.6. Statistical Analysis Plan (SAP) 5
1.6.1. SAP Objectives 5
1.6.2. General Principles 5
1.6.3. Current Protocol (or Current IDMC Charter) 6
1.6.4. Deviations To Those Specified In Study Protocol 6
1.6.5. Additional Analyses To Those Specified In Study Protocol 6
1.6.6. Software 6
2. Analysis 7
2.1. Study Populations 7
2.2. Baseline Characteristics 7
2.3. Effectiveness Outcomes 9
2.3.1. Data Summaries 9
2.3.2. Primary Outcomes 9
2.3.3. Intervention Effects 9
2.3.4. Multiple Imputation 10
2.3.5. Subgroup Analyses 11
2.4. Mediator Variables 11
2.5. Safety Outcomes 12
2.5.1. Premature Withdrawal 12
2.5.2. Adverse Events 12
2.6. Other Variables 12
2.7. Additional Analyses 13
2.7.1. Multiple Imputation 13
2.7.2. Subgroup Analyses 13
2.7.3. Mediation Analyses 14
2.7.4. Other Analyses 14
3. Document History 14
4. Tables 14
5. Figures 14

6. Listings.. 14

1. INTRODUCTION

1.1. STUDY BACKGROUND

The Football Fans in Training (FFIT) study demonstrated that men recruited to exercise groups run through Scottish professional football clubs achieved greater weight loss 12 months later than men randomised to a waiting list control group. The EuroFIT trial is a similar trial, being carried out in top-level clubs in England, the Netherlands, Portugal and Norway. The trial is one part of a wider EU-funded project.

1.2. STUDY OBJECTIVES

The objective of the EuroFIT trial is to assess the effectiveness and cost-effectiveness of the EuroFIT programme in supporting men to increase their daily physical activity and to decrease their daily time spent sitting, at least 12 months after the start of their participation in EuroFIT.

1.3. STUDY DESIGN

A multi-centre, randomised controlled trial.

1.4. SAMPLE SIZE AND POWER

Section 6.5 of the study protocol states:

> *"We have powered the study to detect small, but health-relevant, changes in objective measured physical activity and sedentary time: both are powered (separately, not as a composite primary outcome) at 90% to detect an effect size of 0.25 standard deviation (SD) units at a 2.5% significance level. In relation to physical activity, which has a standard deviation of approximately 4000 steps per day, we will be powered to detect an average increase of at least 1000 steps per day (about 10 minutes on average per day, or 70 minutes per week) of at least moderate intensity activity. In relation to sedentary time, which has a SD of almost 100 minutes/day, we will be powered to detect an average decrease of at least 25 minutes per day spent sitting. A sample size of 400 per group will allow us to detect these minimum changes. We have been conservative in estimating loss to follow up. To achieve this sample size we estimate that we will need to recruit up to 100 interested men per club (around 60-80 per club will be included) (Mutrie et al., 2012).*
>
> *In relation to blood-based biomarkers, we estimate that >70% of participants will opt-in to blood sampling (>245 in each arm). 245 in each arm will have 90% power, at 5% significance, to detect an effect size of 0.29 SD units for a normally-distributed outcome measure. Fasting insulin is the biomarker of principal interest; its SD in non-diabetic adults is ~6*

mU/l (87), thus the study will be powered to detect a change in fasting insulin with the EuroFIT intervention of ~1.7 mU/l."

1.5. Study Population

1.5.1. Inclusion Criteria

Inclusion criteria are:

- men;
- aged 30 to 65;
- self-reported BMI ≥27 kg/m^2 at initial screening;
- consent to randomisation.

1.5.2. Exclusion Criteria

Exclusion criteria are:

- failure to provide at least 4 out of 7 days of usable data from objective measurement of physical activity/sedentary time (activPAL) at baseline;
- do not pass the screening of the adapted PAR-Q+;
- already participating in a health promotion programme at their club.

1.6. Statistical Analysis Plan (SAP)

1.6.1. SAP Objectives

The objective of this SAP is to describe the statistical analyses to be carried out for the EuroFIT trial.

1.6.2. General Principles

Data will primarily be analysed by the intention to treat principle, i.e. in relation to randomised group, regardless of participation in the intervention.

Data will be summarised overall and by randomised group, where applicable. For all data items, the number of available observations and number of missing values will be reported. Continuous data will be summarised with the mean, standard deviation, median, quartiles and range. Categorical data will be summarised with frequencies and percentages of non-missing values.

Analysis of outcomes at each time point will use linear mixed effects regression methods (normal, logistic or other generalised linear model, as required), including random effects for country and football club, and fixed effects for study group and baseline measurement of the outcome (where applicable). In some instances, football club may be included as a fixed effect to account for the variation between club and country, with no random effects in the model.

Between-group comparisons will be reported as estimated mean differences (or other statistic, according to the regression model used) with a 95% confidence interval and p-value. Confidence intervals and p-values will be two-sided and derived by the Wald method.

For primary outcomes, measurements at all time points will also be analysed simultaneously using repeated measures regression methods, including fixed effects for study group, time point, and study group × time interactions. Baseline measurements will be included as outcome variables. A general (unstructured) covariance structure will be assumed for repeat measurements within each individual. For model simplicity, football club will be included as a fixed effect to account for the variation between club and country.

Results will be presented in the first instance without imputation of missing data. For primary outcomes, results will also be presented from analyses using multiple imputation for missing baseline data.

1.6.3. Current Protocol (or Current IDMC Charter)

The current study protocol at the time of writing is version 10, dated 24th July 2015. Future amendments to the protocol will be reviewed for their impact on this SAP, which will be updated only if necessary. If no changes are required to this SAP following future amendments to the study protocol, this will be documented as part of the Robertson Centre Change Impact Assessment processes.

1.6.4. Deviations To Those Specified In Study Protocol

This SAP does not represent a deviation from the analyses specified in the study protocol. Should any deviations be required, based on the characteristics of the study data, these will be justified and documented in the final study report.

1.6.5. Additional Analyses To Those Specified In Study Protocol

This SAP does not include any analyses additional to those specified in the study protocol. Should any additional analyses be considered worthwhile, these will be included in a Supplementary Statistical Analysis Plan.

1.6.6. Software

All analyses will be carried out using recognised statistical software. This is likely to be SAS for Windows v9.2, and/or R for Windows v3.0.0, or more recent versions of these programs.

2. ANALYSIS

2.1. STUDY POPULATIONS

The numbers of men who were screened will be reported overall, by country, and by club. The number and percentage of those screened who were randomised will be presented overall, by country, and by club. For those not randomised, the reasons for exclusion will be summarised overall, by country and by club.

The number of men screened and the number and percentage randomised will be reported overall, by country, and by club, for the subgroup of men who consent to provide blood samples.

For all randomised men, and for the subgroup who consent to provide blood samples, the number and percentage who provide data at 12 weeks and at 12 months will be reported (for those in the blood sampling subgroup, this will entail attending the 12 month follow-up visit and providing a blood sample).

2.2. BASELINE CHARACTERISTICS

Baseline characteristics will be summarised for all randomised men and by randomised group. No statistical comparisons will be made between randomised groups. The following baseline characteristics will be presented:

- age (years);
- ethnicity, marital status, years of education, current employment status, income;
- weight (kg), height (m), BMI (kg/m^2) and waist circumference (cm);
- systolic and diastolic blood pressure (mmHg), heart rate (bpm);
- activPal measures (based on valid days):
    - number of valid days;
    - average daily step count;
    - average daily time spent sitting (minutes/day);
    - average daily wake wear time (minutes/day);
    - average daily time spent standing (minutes/day);
    - average daily time spent stepping (minutes/day);
    - average daily time spent upright (minutes/day);
    - average daily number of sit-to-stand transitions;
    - average daily number of sitting/lying bouts lasting 0-30 mins;
    - average daily number of sitting/lying bouts lasting 30-60 mins;
    - average daily number of sitting/lying bouts lasting 60-120 mins;
    - average daily number of sitting/lying bouts lasting 120+ mins;
    - average daily time spent sitting/lying in bouts lasting 0-30 mins (minutes/day);
    - average daily time spent sitting/lying in bouts lasting 30-60 mins (minutes/day);

    - average daily time spent sitting/lying in bouts lasting 60-120 mins (minutes/day);
    - average daily time spent sitting/lying in bouts lasting 120+ mins (minutes/day);
    - average daily sitting time >10 hours/day;
- self-reported physical activity:
    - average daily time spent walking (MET-minutes);
    - average daily time spent in other moderate physical activity (MET-minutes);
    - average daily time spent in vigorous physical activity (MET-minutes);
    - average daily total physical activity (MET-minutes);
    - meeting PA recommendation (WHO definition);
- self-reported sedentary time:
    - average daily time (hours) spent sitting, overall and by weekdays/weekends;
    - average daily time (hours) spent sitting for transport, at work, watching TV, using a computer at home, or other leisure activities, overall and by weekdays/weekends;
    - average daily time (hours) spent standing;
    - average daily time (hours) spent sleeping;
- activity choice index;
- dietary intake (adapted DINE questionnaire):
    - frequency of breakfast consumption;
    - fatty food score;
    - sugary food score;
    - fruit score;
    - vegetable score;
    - fruit and vegetable score;
- total alcohol consumption (units per weeks);
- smoking status, number smoked per day;
- wellbeing (Cantril ladder);
- self-esteem (Rosenberg);
- subjective vitality scale;
- prevalent long-standing illness;
- upper and lower joint pain scores;
- recent injuries;
- Sport Spectator Identification Scale;
- motivation for physical activity, ego/task involvement;
- weight management strategies;
- weight loss activities;
- cardio-metabolic biomarkers:
    - glucose, insulin, HbA1c, lipids (total and HDL cholesterol, and triglycerides), liver function (ALT, AST, GGT).

Baseline characteristics will also be summarised in relation to whether or not men provided activPal data at 12 weeks and at 12 months. Those with and without outcome data will be compared using t-tests or Wilcoxon-Mann-

Whitney tests as appropriate, for continuous or ordinal variables, and Fisher's Exact test for categorical variables.

2.3. Effectiveness Outcomes

2.3.1. Data Summaries

All effectiveness outcome measures will be summarised at 12 weeks (post-intervention) and 12 months after randomisation, or those time points at which they are measured. For continuous outcome measures, changes from baseline will also be summarised.

2.3.2. Primary Outcomes

The primary study outcomes are the average daily step count and average daily sedentary (sitting) time, obtained from the activPal at 12 months. All other outcomes will be considered secondary.

2.3.3. Intervention Effects

Each outcome measure will be summarised for all randomised men and by randomised group, and will be analysed using mixed effects regression models as described in section 1.6.2. In addition, for the primary outcome measures, 97.5% confidence intervals for the intervention effects will be reported to allow for multiple comparisons.

The following outcome measures will be reported:

- activPal measures (based on valid days) at 12 weeks and 12 months:
  - average daily step count;
  - average daily time spent sitting (minutes/day);
  - average daily time spent standing (minutes/day);
  - average daily time spent stepping (minutes/day);
  - average daily time spent upright (minutes/day);
  - average daily number of sit-to-stand transitions;
  - average daily number of sitting/lying bouts lasting 0-30 mins;
  - average daily number of sitting/lying bouts lasting 30-60 mins;
  - average daily number of sitting/lying bouts lasting 60-120 mins;
  - average daily number of sitting/lying bouts lasting 120+ mins;
  - average daily time spent sitting/lying in bouts lasting 0-30 mins (minutes/day);
  - average daily time spent sitting/lying in bouts lasting 30-60 mins (minutes/day);
  - average daily time spent sitting/lying in bouts lasting 60-120 mins (minutes/day);
  - average daily time spent sitting/lying in bouts lasting 120+ mins (minutes/day);
  - average daily sitting time >10 hours/day;

- weight (kg), BMI (kg/m^2) and waist circumference (cm) at 12 weeks and 12 months;
- systolic and diastolic blood pressure (mmHg) and heart rate (bpm) at 12 weeks and 12 months;
- self-reported physical activity at 12 weeks and 12 months:
  - average daily time spent walking (MET-minutes);
  - average daily time spent in other moderate physical activity (MET-minutes);
  - average daily time spent in vigorous physical activity (MET-minutes);
  - average daily total physical activity (MET-minutes);
  - meeting PA recommendation (WHO definition);
- self-reported sedentary time at 12 weeks and 12 months:
  - average daily time (hours) spent sitting, overall and by weekdays/weekends;
  - average daily time (hours) spent sitting for transport, at work, watching TV, using a computer at home, or other leisure activities, overall and by weekdays/weekends;
  - average daily time (hours) spent standing;
  - average daily time (hours) spent sleeping;
- activity choice index at 12 weeks and 12 months;
- dietary intake (adapted DINE questionnaire) at 12 weeks and 12 months:
  - frequency of breakfast consumption;
  - fatty food score;
  - sugary food score;
  - fruit score;
  - vegetable score;
  - fruit and vegetable score;
- total alcohol consumption (units per weeks) at 12 weeks and 12 months;
- wellbeing (Cantril ladder) at 12 weeks and 12 months;
- self-esteem (Rosenberg) at 12 weeks and 12 months;
- subjective vitality scale at 12 weeks and 12 months;
- upper and lower joint pain scores at 12 weeks and 12 months;
- cardio-metabolic biomarkers at 12 months:
  - glucose, insulin, HbA1c, lipids (total and HDL cholesterol, and triglycerides), liver function (ALT, AST, GGT)

2.3.4. Multiple Imputation

For the primary outcomes, if there is any missing data for the baseline measurement of the outcome, a multiple imputation procedure will be applied to impute the missing baseline data. The prediction model used will be based on available baseline data, and will be described in full in the final statistical outputs.

2.3.5. Subgroup Analyses

For the primary outcomes, and for weight at 12 months, subgroup analyses will be performed for potential moderator variables. The following baseline characteristics will be considered as potential moderators of any intervention effects:

- age;
- marital status;
- years of education;
- employment status;
- income;
- club;
- country;
- baseline BMI;
- prevalent longstanding illness;
- upper and lower joint pain scores.

Intervention effect moderation will be assessed by first extending the primary analysis regression model to include an adjustment for the potential moderator. Categorical moderators may be recoded if the numbers in some groups are small. This model will then be extended further to include a moderator-by-intervention interaction. Intervention effect estimates with 95% confidence intervals and p-values will be reported for the adjusted model and the interaction model. For the interaction model, the intervention effect estimates will be specific to each level of the moderator (if categorical) or to the 10th, 25th, 50th, 75th and 90th percentiles of the moderator (if continuous). The interaction p-value will also be reported.

The intervention effect estimates and confidence intervals will also be presented graphically, in forest plots.

2.4. Mediator Variables

Certain variables have been measured as potential mediators of any intervention effects. The following potential mediators will be measured in both randomised groups, at 12 weeks and 12 months, unless otherwise stated:

- motivation for physical activity;
- ego/task involvement, measured at 12 months;
- club identification;
- weight management strategies;
- weight loss activities.

These variables will be summarised and analysed in the same way as effectiveness outcomes. For weight management strategies and weight loss activities, only the scores will be analysed as outcomes, the individual components will only be summarised.

Incident injuries and joint pain will also be considered as potential mediators.

The following potential mediator variables will be measured only in the intervention group at 12 weeks (unless otherwise stated):

- support provided by coach (autonomy, competence, relatedness);
- need thwarting by coach (autonomy, competence, relatedness);
- climate of coaching sessions (performance, mastery);
- relatedness to group;

These variables will be summarised for the intervention group as a whole.

For those in the intervention group, the number of sessions attended will be summarised for the intervention group as a whole, and will be treated as a potential mediator variable.

2.5. Safety Outcomes

2.5.1. Premature Withdrawal

The number and percentage of men who withdraw from the study between baseline and 12 weeks, and between 12 weeks and 12 months, will be reported for all randomised men and by randomised group. Reasons for withdrawal will also be summarised.

The number and percentage of men in the intervention group who withdraw from the exercise programme will be reported.

2.5.2. Adverse Events

Recent injuries will be summarised at 12 weeks and 12 months for all randomised men and by randomised group.

No statistical comparisons will be made between randomised groups.

2.6. Other Variables

The following variables will be summarised at 12 weeks and 12 months for all randomised men and by randomised group (where applicable). No statistical comparisons will be made between randomised groups:

- smoking status, number smoked per day;
- employment status;
- percentage change in weight (kg);
- BMI category;
- activPAL data:
  - number of valid days;
  - average daily wake wear time based on valid days (minutes/day);
- prevalent long-standing illness;

- upper and lower joint pain (frequency, severity , limiting);
- satisfaction from physical activity (autonomy, competence, relatedness), measured at 12 months.
- process evaluation (summarised overall):
  - Baseline:
    - How did you hear about programme?
    - Reasons for joining
    - Role at club
  - 12 weeks
    - Intervention Group
      - Programme evaluation (encouragement/usefulness)
      - Number of sessions attended
      - Rating of programme and coaches
      - Amount willing to pay for EuroFIT/SitFIT
      - Recommend the programme
  - 12 months
    - Changes in Lifestyle (summarised by randomised group)
    - Activities since programme (Intervention only)
    - Ripple effect of programme (Intervention only)

2.7. Additional Analyses

Following the completion of the analyses specified in this SAP, it is intended that some additional analyses will be carried out including blood inflammatory marker analysis. At this stage, a Supplementary Statistical Analysis Plan will be agreed before any additional analyses are carried out.

2.7.1. Multiple Imputation

For the primary outcomes, the sensitivity of the main analysis to missing outcome data may be assessed by multiple imputation. The prediction model used will be based on data available at baseline and at 12 weeks in both randomised groups, but will not include randomised group, and will be described in full in the final statistical outputs.

After considering the intervention effects on the secondary outcome measures, and the impact of multiple imputation on the primary analyses, similar imputation procedures may be applied to selected secondary outcome measures.

2.7.2. Subgroup Analyses

The subgroup analyses specified for the primary outcomes in this SAP may be extended to include additional secondary outcomes and/or additional potential moderator variables.

2.7.3. Mediation Analyses

After considering the intervention effects on the primary and secondary outcome measures, and the potential mediator variables (where measured in both randomised groups), a plan will be drawn up as to which outcomes and potential mediators will be taken forward to additional mediation analyses. In these analyses, the mixed effects regression model used to assess the intervention effect on the outcome will be extended to include (changes in) the potential mediator variable. A bootstrapping procedure will be applied to estimate the intervention effect under both models, to obtain an estimate of the change in the intervention effect estimate after adjustment for the mediator, with a 95% confidence interval and p-value.

2.7.4. Other Analyses

If any additional analyses not covered in this SAP are considered worthwhile, they will be included in the Supplementary Statistical Analysis Plan.

3. Document History

This is v1.0 of the SAP for the EuroFIT trial, dated 14th November 2017, the initial creation.

4. Tables

Dummy tables will be produced during the development of the statistical analysis programs for review and feedback. Approval of the content of the final statistical outputs will be a requirement for database lock.

5. Figures

Dummy figures will be produced during the development of the statistical analysis programs for review and feedback. Approval of the content of the final statistical outputs will be a requirement for database lock.

6. Listings

No formal data listings will be produced as part of the final statistical outputs. Raw and derived datasets will be made available to the study team after the final statistical outputs have been produced.
