# Supplementary material for: The effect of a programme to improve men’s sedentary time and physical activity: The European Fans in Training (EuroFIT) randomised controlled trial
Source: PLoS Med. 2019 Feb 5;16(2):e1002736. doi: 10.1371/journal.pmed.1002736 (PMC6363143; doi:10.1371/journal.pmed.1002736)
Supplement: S1 Tables — (PDF) [file pmed.1002736.s008.pdf]

S1 Table. Supplementary tables

Table A. Baseline sociodemographic characteristics of participants who did or did not provide primary outcome data (activPAL) at the post-programme and 12 month assessment points.

	activPAL Data Available Post Programme			activPAL Data Available at 12 Months		
	Yes (N=935)	No (N=178)	p-value	Yes (N=921)	No (N=192)	p-value
Age (years)	[0 missing]	[0 missing)		[0 missing]	[0 missing]	
Mean (SD)	46.1 (8.9)	44.0 (8.4)	0.002	46.1 (8.9)	44.2 (8.2)	0.005
'Native' to Study Country	[14 missing]	[2 missing)		[13 missing]	[3 missing]	
Participant, mother and father born there	832 (90.3%)	151 (85.8%)	0.079	820 (90.3%)	163 (86.2%)	0.115
Years of Education	[14 missing]	[2 missing)		[13 missing]	[3 missing]	
<12 years	210 (22.8%)	46 (26.1%)		205 (22.6%)	51 (27.0%)	
12-15 years	356 (38.7%)	65 (36.9%)	0.633	345 (38.0%)	76 (40.2%)	0.187
16+ years	355 (38.5%)	65 (36.9%)		358 (39.4%)	62 (32.8%)	
Employment Status	[15 missing]	[4 missing]		[15 missing]	[4 missing]	
Working Full Time	742 (80.7%)	140 (80.5%)		727 (80.2%)	155 (82.4%)	
Working Part Time	64 (7.0%)	11 (6.3%)	0.935	62 (6.8%)	13 (6.9%)	0.829
Not Working (Unable)	44 (4.8%)	10 (5.7%)		45 (5.0%)	9 (4.8%)	
Not Working (Other)	70 (7.6%)	13 (7.5%)		72 (7.9%)	11 (5.9%)	
Income	[14 missing]	[3 missing]		[14 missing]	[3 missing]	
Category 1 (Low)	50 (5.4%)	14 (8.0%)		52 (5.7%)	12 (6.3%)	
Category 2	160 (17.4%)	28 (16.0%)		156 (17.2%)	32 (16.9%)	
Category 3	197 (21.4%)	37 (21.1%)		200 (22.1%)	34 (18.0%)	
Category 4	241 (26.2%)	28 (16.0%)	0.036	235 (25.9%)	34 (18.0%)	0.051
Category 5 (High)	197 (21.4%)	53 (30.3%)		195 (21.5%)	55 (29.1%)	
Don't know	15 (1.6%)	3 (1.7%)		14 (1.5%)	4 (2.1%)	
Rather not answer	61 (6.6%)	12 (6.9%)		55 (6.1%)	18 (9.5%)	
Relationship Status	[14 missing]	[2 missing]		[13 missing]	[3 missing]	
Married/Living with Partner	754 (81.9%)	132 (75.0%)	0.037	742 (81.7%)	144 (76.2%)	0.085
Other	167 (18.1%)	44 (25.0%)		166 (18.3%)	45 (23.8%)	

Table A. Baseline sociodemographic characteristics of participants who did or did not provide primary outcome data (activPAL) at the post-programme and 12 month assessment points.

Long-Standing Illnesses	[5 missing]	[1 missing]		[5 missing]	[1 missing]	
No	564 (60.6%)	108 (61.0%)		555 (60.6%)	117 (61.3%)	
Yes, not limiting	247 (26.6%)	42 (23.7%)	0.558	244 (26.6%)	45 (23.6%)	0.524
Yes, Limiting	119 (12.8%)	27 (15.3%)		117 (12.8%)	29 (15.2%)	

Table B. Baseline measurements of outcome variables for participants who did or did not provide primary outcome data (activPAL) at the post-programme and 12 month assessment points. Data are mean (SD) or N (%). P-values for continuous measures are from two-sample t-tests (T), or Wilcoxon-Mann-Whitney tests (W). P-values for binary measures are from Fisher's Exact test (F).

	activPAL Data Available Post Programme			activPAL Data Available at 12 Months		
	Yes (N=935)	No (N=178)	p-value	Yes (N=921)	No (N=192)	p-value
activPAL Measures	[14 missing]	[2 missing]		[13 missing]	[3 missing]	
Average daily step count	8507 (3195)	7652 (2994)	<0.001[T]	8535 (3210)	7595 (2908)	<0.001[T]
Average daily time spent sitting (minutes)	623.0 (109.2)	637.4 (106.7)	0.104[T]	621.2 (109.7)	644.4 (102.9)	0.005[T]
Number of valid days	6.8 (0.5)	6.5 (0.9)	<0.001[W]	6.8 (0.6)	6.7 (0.7)	0.073[W]
Average waking wear time (minutes)	976.8 (67.2)	969.4 (75.4)	0.194[T]	976.2 (68.2)	972.7 (70.2)	0.531[T]
Average daily time spent standing (minutes)	246.5 (83.8)	234.9 (80.3)	0.083[T]	247.5 (83.9)	231.2 (79.6)	0.011[T]
Average daily time spent stepping (minutes)	107.3 (37.6)	97.1 (34.5)	<0.001[T]	107.5 (37.7)	97.1 (34.0)	<0.001[T]
Average daily time spent upright (minutes)	353.8 (105.5)	332.0 (101.1)	0.010[T]	355.0 (105.7)	328.3 (99.2)	<0.001[T]
Physical Measures	[4 missing]	[0 missing]		[4 missing]	[0 missing]	
Weight (kg)	105.3 (17.0)	109.1 (20.4)	0.009[T]	105.1 (17.4)	109.7 (18.1)	0.001[T]
Body Mass Index (kg/m^2)	33.1 (4.4)	34.1 (5.7)	0.008[T]	33.1 (4.6)	33.9 (5.1)	0.027[T]
Waist Circumference (cm)	111.0 (11.9)	112.7 (13.9)	0.082[T]	110.8 (12.0)	113.5 (13.4)	0.006[T]
IPAQ Measures	[6 missing]	[1 missing]		[6 missing]	[1 missing]	
Total Physical Activity (MET-mins/week)	2312 (2740)	2311 (2754)	0.416[W]	2380 (2797)	1985 (2431)	0.018[W]
Recommended activity (MVPA ≥150 minutes per week)	424 (45.6%)	82 (46.3%)	0.870[F]	425 (46.4%)	81 (42.4%)	0.338[F]
Marshall Questionnaire	[14 missing]	[2 missing]		[14 missing]	[2 missing]	
Sitting Time (hours per day)	11.1 (4.1)	11.8 (4.9)	0.089[W]	11.1 (4.2)	12.0 (4.4)	0.006[W]
Activity Choice Index	[113 missing]	[21 missing]		[109 missing]	[25 missing]	
(Range 1-5)	2.4 (0.7)	2.3 (0.6)	0.023[T]	2.4 (0.7)	2.2 (0.6)	<0.001[T]

Table C: Sensitivity analysis: Multiple imputation of follow up data for primary outcomes. Intervention effects estimated as mean differences (95% CI), derived from mixed effects regression models.

		Intervention Effect		
		Estimated Difference	(95% CI)	p
Number of steps (count per day)	Post-Program	1204	(866, 1542)	p <0·001
	12 Months	657	(333, 982)	p <0·001
Sedentary time (minutes per day)	Post-Program	-13·5	(-24·1, -2·8)	p=0·013
	12 Months	-1·4	(-12·5, 9·7)	p=0·808

Table D: Sensitivity analysis: Primary outcomes adjusted for wear time. Number of steps each day divided by total wear time and expressed as steps per hour; sedentary time each day divided by total wear time and expressed as the percentage of time in sedentary behavior. Intervention effects estimated as mean differences (95% CI), derived from mixed effects regression models.

		Intervention Effect		
		Estimated Difference	(95% CI)	p
Number of steps (average per hour)	Baseline	Difference		
	Post-Program	73·3	(52·5, 94·0)	p <0·001
	12 Months	40·9	(21·0, 60·8)	p <0·001
Sedentary time (% of time spent sedentary)	Baseline	Difference		
	Post-Program	-1·7	(-2·61, -0·7)	p <0·001
	12 Months	-0·2	(-1·2, 0·8)*	p=0·653

Table E: Repeated Measures Analysis. Intervention effects estimated as mean differences (95% CI). Results shown for analysis of raw data, with multiple imputation, and adjusted for wear time (in two ways: first, by dividing daily step counts and sedentary time by total daily wear time, expressed as number of steps per hour, or as % time spent sedentary; second, by using the raw data as the model outcome, and adjusting for the average daily wear time as a covariate).

		Intervention Effect		
		Estimate	(95% CI)	p
Raw data				
Number of steps	Post-Program	1163	(774, 1551)	p <0·001
(count per day)	12 Months	633	(284, 981)	p <0·001
Sedentary time	Post-Program	-9·8	(-21·8, 2·2)	p = 0·108
(minutes per day)	12 Months	1·7	(-10·4, 13·8)	p = 0·783
With multiple imputation				
Number of steps	Post-Program	1155	(767, 1543)	p <0·001
(count per day)	12 Months	620	(271, 970)	p <0·001
Sedentary time	Post-Program	-9·4	(21·4, 2·6)	p = 0·126
(minutes per day)	12 Months	2·1	(-9·9, 14·2)	p = 0·731
Adjusted for wear time (outcomes expressed relative to total daily wear time)				
Number of steps	Post-Program	71·5	(46·7, 94·2)	p <0·001
(average per hour)	12 Months	37·5	(16·0, 59·0)	p <0·001
Sedentary time	Post-Program	-1·29	(-2·40, -0·18)	p = 0·023
(% of time spent sedentary)	12 Months	-0·04	(-1·12, 1·04)	p = 0·940

Table F: Repeated Measures Analysis. Intervention effects estimated as mean differences (95% CI). Results shown for analysis of raw data, with multiple imputation, and adjusted for wear time (in two ways: first, by dividing daily step counts and sedentary time by total daily wear time, expressed as number of steps per hour, or as % time spent sedentary; second, by using the raw data as the model outcome, and adjusting for the average daily wear time as a covariate).

Adjusted for wear time (models adjusted for average daily wear time as covariate)				
Number of steps	Post-Program	1136	(754, 1519)	p <0·001
(count per day)	12 Months	614	(271, 958)	p <0·001
Sedentary time	Post-Program	-11·74	(-22·43, -1·06)	p = 0·031
(minutes per day)	12 Months	0·28	(-10·23, 10·78)	p = 0·959

Table G: Objectively assessed physical activity and sedentary time outcome measures for participants allocated to the EuroFIT program immediately (Intervention) or after 12 months (Comparison). Data are mean (SD), at Baseline and Post-Program, for those participants with data at both time points. Intervention effects estimated are mean differences (95% CI), derived from mixed effects regression models.

		Intervention		Comparison		Intervention Effect		
		N	Mean (SD)	N	Mean (SD)	Estimate	(95% CI)	p
Primary Outcomes (activPAL activity monitor)								
Number of steps (steps per day)	Baseline<br>Post-Program	464	8543 (3201)<br>9801 (3730)	468	8471 (3193)<br>8511 (3264)	1208	(869, 1546)	p <0·001
Sedentary time (minutes per day)	Baseline<br>Post-Program	464	621 (109)<br>597 (109)	468	625 (109)<br>614 (105)	-14·4	(-25·1, -3·8)	p=0·008
Secondary Outcomes (activPAL activity monitor)								
Number of valid days (days)	Baseline<br>Post-Program	478	6.8 (0.6)<br>6.3 (1.2)	476	6.8 (0.6)<br>6.4 (1.0)	-0·11	(-0·25, 0·02)	p=0·101
Waking wear time (minutes per day)	Baseline<br>Post-Program	464	976 (68)<br>968 (70)	468	978 (67)<br>967 (73)	2·24	(-5·36, 9·84)	p=0·563
Standing time (minutes per day)	Baseline<br>Post-Program	464	247 (87)<br>252 (85)	468	246 (81)<br>246 (82)	5·3	(-2·6, 13·1)	p=0·187
Stepping time (minutes per day)	Baseline<br>Post-Program	464	108 (37)<br>120 (40)	468	107 (39)<br>108 (39)	11·2	(7·6, 14·8)	p <0·001
Upright time (minutes per day)	Baseline<br>Post-Program	464	355 (107)<br>371 (106)	468	353 (104)<br>353 (103)	16·5	(6·8, 26·2)	p <0·001

Table H: Objectively assessed physical activity and sedentary time outcome measures for participants allocated to the EuroFIT program immediately (Intervention) or after 12 months (Comparison). Data are mean (SD), at Baseline and 12 months, for those participants with data at both time points. Intervention effects estimated are mean differences (95% CI), derived from mixed effects regression models.

		Intervention		Comparison		Intervention Effect		
		N	Mean (SD)	N	Mean (SD)	Estimate	(95% CI)	p
Primary Outcomes (activPAL activity monitor)								
Number of steps<br>(steps per day)	Baseline<br>12 Months	449	8585 (3226)<br>9261 (3513)	466	8486 (3197)<br>8502 (3155)	678	(309, 1048)	p <0·001
Sedentary time<br>(minutes per day)	Baseline<br>12 Months	449	617 (110)<br>612 (109)	466	625 (109)<br>619 (109)	-1·6	(-14·3, 11·0)	p=0·772
Secondary Outcomes (activPAL activity monitor)								
Number of valid days<br>(days)	Baseline<br>12 Months	462	6.8 (0.6)<br>6.2 (1.1)	475	6.8 (0.6)<br>6.3 (1.0)	-0·08	(-0·21, 0·05)	p=0·224
Waking wear time<br>(minutes per day)	Baseline<br>12 Months	449	975 (70)<br>970 (77)	466	977 (67)<br>970 (67)	0·42	(-7·63, 8·48)	p=0·918
Standing time<br>(minutes per day)	Baseline<br>12 Months	449	251 (87)<br>244 (83)	466	245 (81)<br>244 (83)	-3·7	(-11·9, 4·5)	p=0·376
Stepping time<br>(minutes per day)	Baseline<br>12 Months	449	108 (37)<br>114 (39)	466	107 (38)<br>107 (38)	6·0	(2·4, 9·6)	p=0·001
Upright time<br>(minutes per day)	Baseline<br>12 Months	449	358 (108)<br>358 (103)	466	352 (103)<br>351 (106)	2·2	(-7·9· 12·2)	p = 0·669
