# Supplementary material for: The effect of a programme to improve men’s sedentary time and physical activity: The European Fans in Training (EuroFIT) randomised controlled trial
Source: PLoS Med. 2019 Feb 5;16(2):e1002736. doi: 10.1371/journal.pmed.1002736 (PMC6363143; doi:10.1371/journal.pmed.1002736)
Supplement: S1 Fig — (PDF) [file pmed.1002736.s009.pdf]

S1 Fig. Intervention effect heterogeneity: group by moderator interaction on physical activity, sedentary time and weight

a)

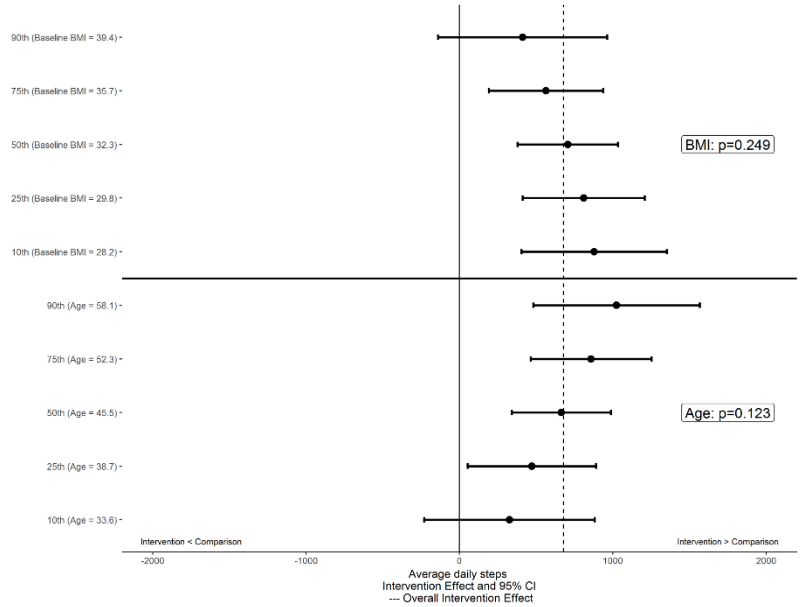

b)

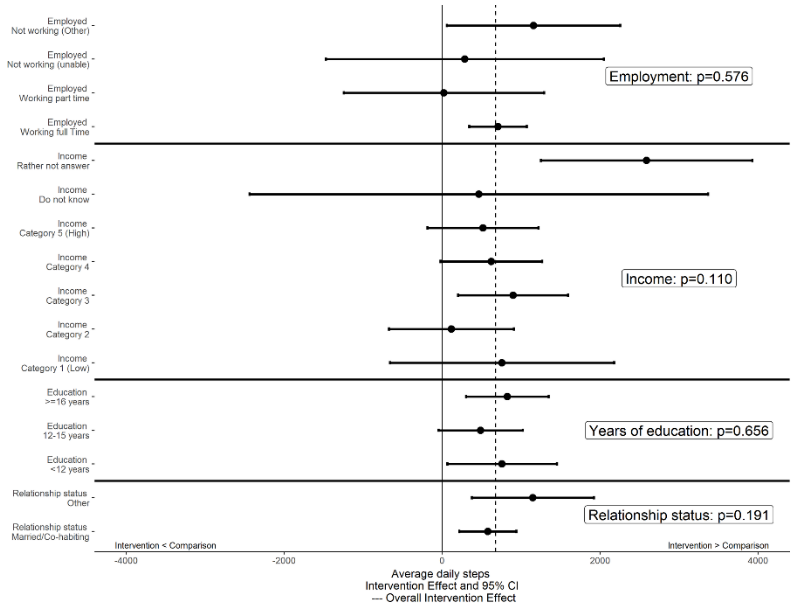

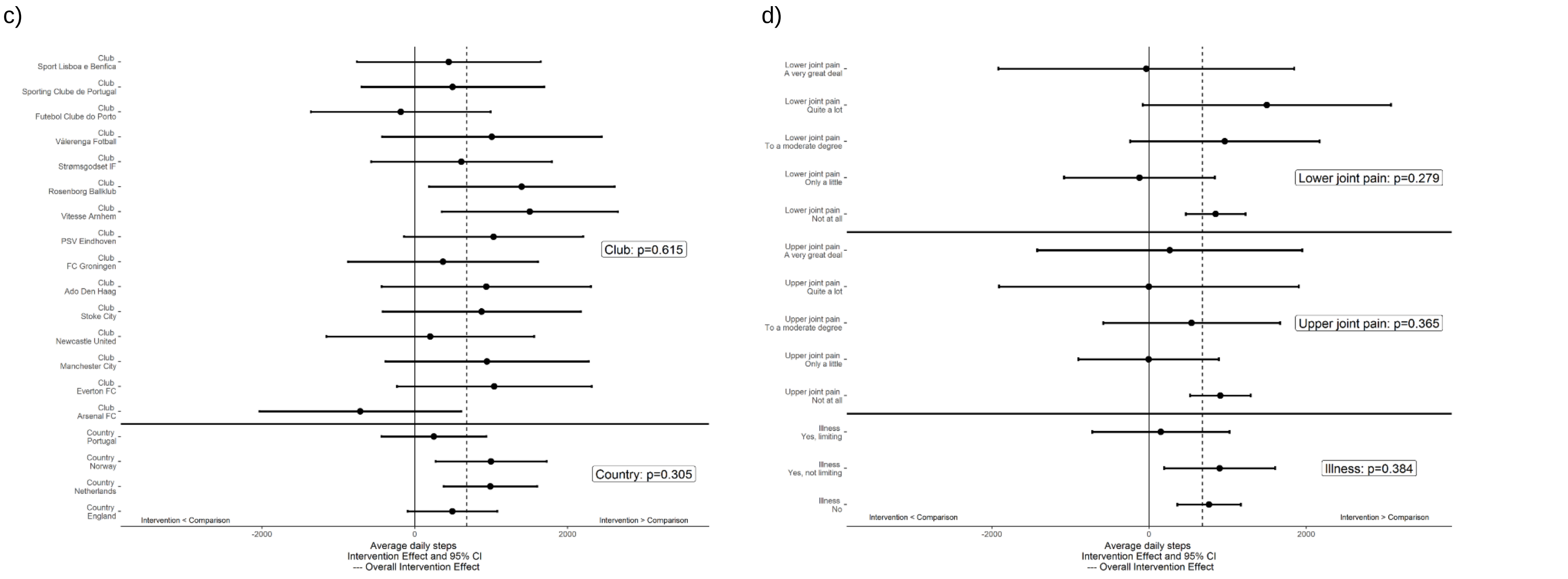

Figure A – subgroup effects, activPAL steps per day, a = age and BMI, b = Relationship status, years of education, income and employment, c = Country and club, d = Long standing illness, upper joint pain and lower joint pain score

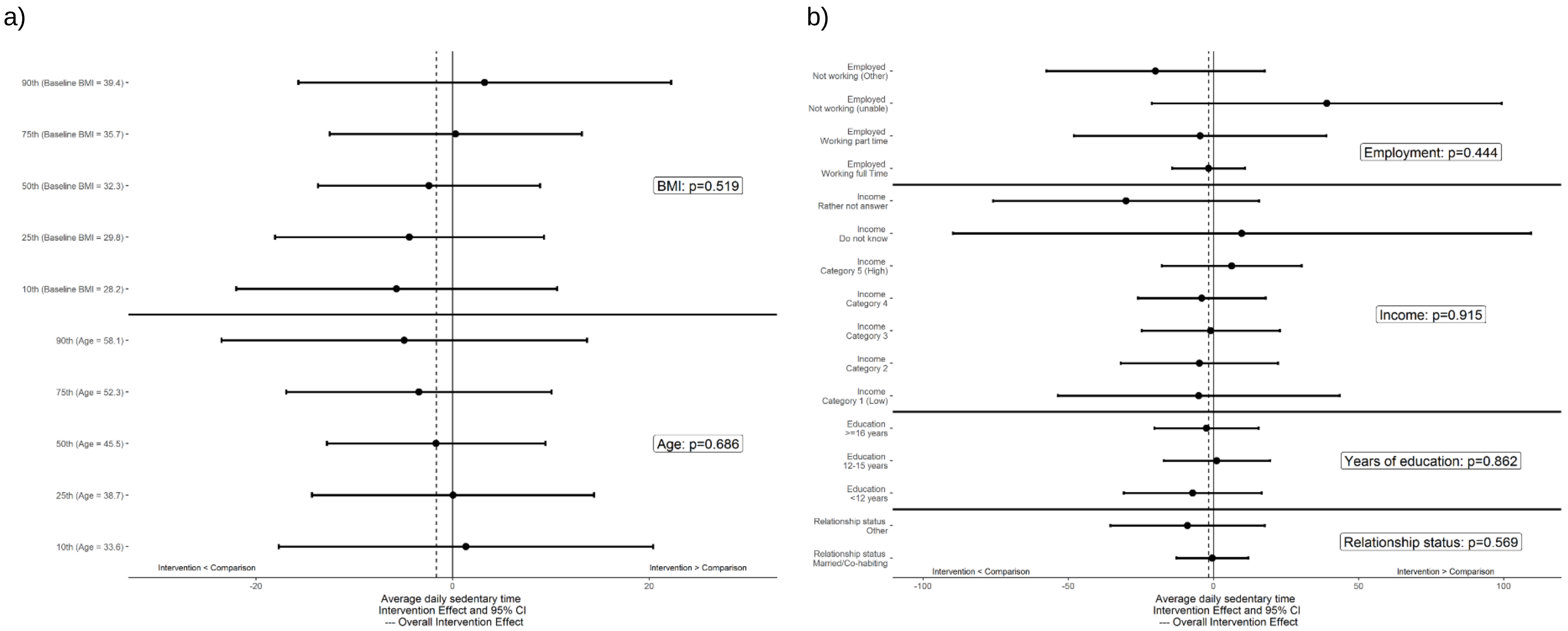
a)
90th (Baseline BMI = 39.4)
75th (Baseline BMI = 35.7)
50th (Baseline BMI = 32.3)
25th (Baseline BMI = 29.8)
10th (Baseline BMI = 28.2)
90th (Age = 58.1)
75th (Age = 52.3)
50th (Age = 45.5)
25th (Age = 38.7)
10th (Age = 33.6)
BMI: p=0.519
Age: p=0.686
Intervention < Comparison
Intervention > Comparison
-20
0
20
Average daily sedentary time
Intervention Effect and 95% CI
--- Overall Intervention Effect
b)
Employed Not working (Other)
Employed Not working (unable)
Employed Working part time
Employed Working full Time
Income Rather not answer
Income Do not know
Income Category 5 (High)
Income Category 4
Income Category 3
Income Category 2
Income Category 1 (Low)
Education >=16 years
Education 12-15 years
Education <12 years
Relationship status Other
Relationship status Married/Co-habiting
Employment: p=0.444
Income: p=0.915
Years of education: p=0.862
Relationship status: p=0.569
Intervention < Comparison
Intervention > Comparison
-100
-50
0
50
100
Average daily sedentary time
Intervention Effect and 95% CI
--- Overall Intervention Effect

c)

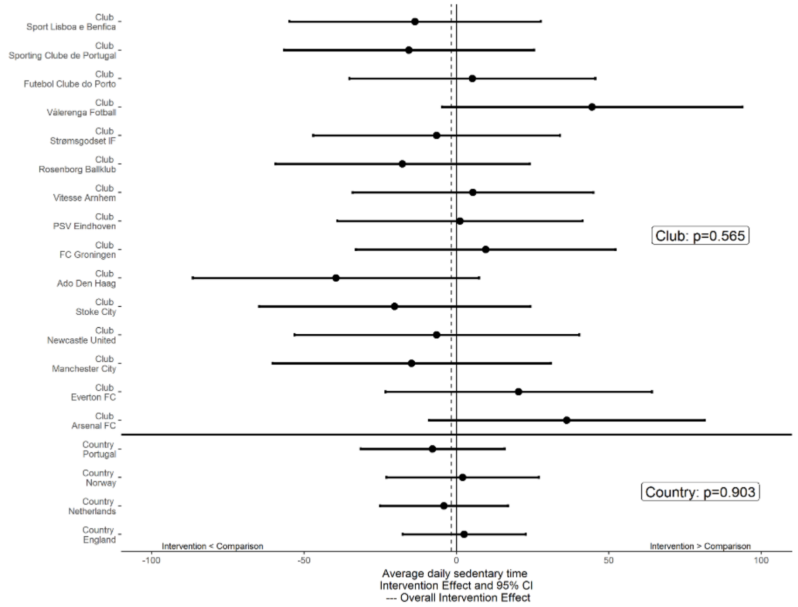

d)

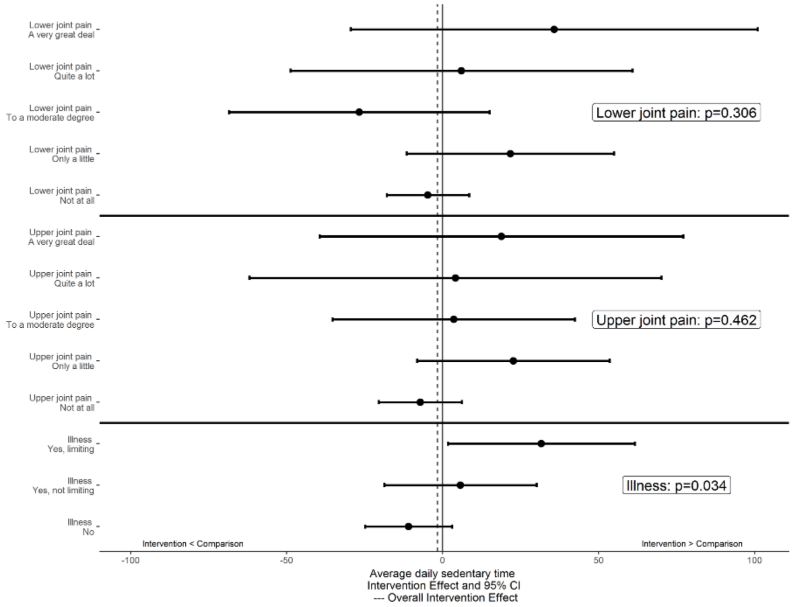

Figure B – subgroup effects, activPAL sedentary time, a = age and BMI, b = Relationship status, years of education, income and employment, c = Country and club, d = Long standing illness, upper joint pain and lower joint pain score·

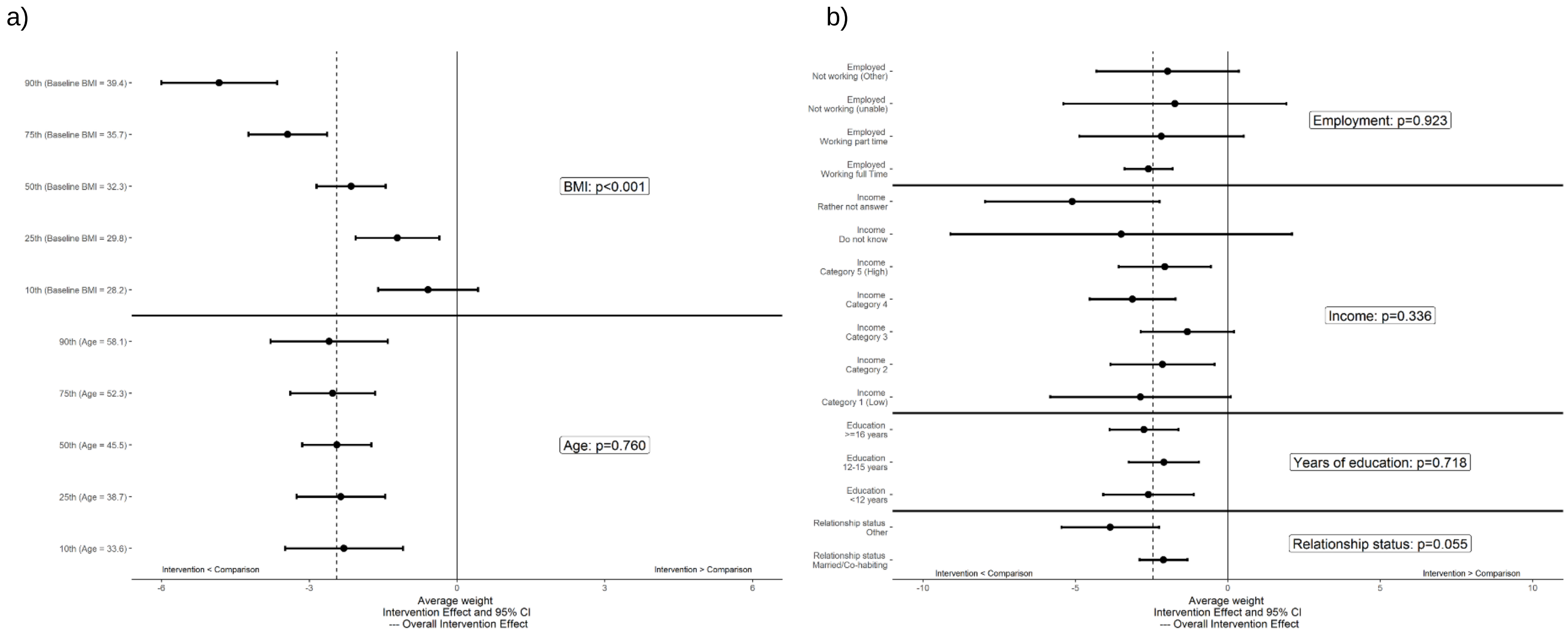
a)
90th (Baseline BMI = 39.4)
75th (Baseline BMI = 35.7)
50th (Baseline BMI = 32.3)
25th (Baseline BMI = 29.8)
10th (Baseline BMI = 28.2)
BMI: p<0.001
90th (Age = 58.1)
75th (Age = 52.3)
50th (Age = 45.5)
25th (Age = 38.7)
10th (Age = 33.6)
Age: p=0.760
Intervention < Comparison
Intervention > Comparison
-6
-3
0
3
6
Average weight
Intervention Effect and 95% CI
--- Overall Intervention Effect
b)
Employed Not working (Other)
Employed Not working (unable)
Employed Working part time
Employed Working full Time
Employment: p=0.923
Income Rather not answer
Income Do not know
Income Category 5 (High)
Income Category 4
Income Category 3
Income Category 2
Income Category 1 (Low)
Income: p=0.336
Education >=16 years
Education 12-15 years
Education <12 years
Years of education: p=0.718
Relationship status Other
Relationship status Married/Co-habiting
Relationship status: p=0.055
Intervention < Comparison
Intervention > Comparison
-10
-5
0
5
10
Average weight
Intervention Effect and 95% CI
--- Overall Intervention Effect

c)

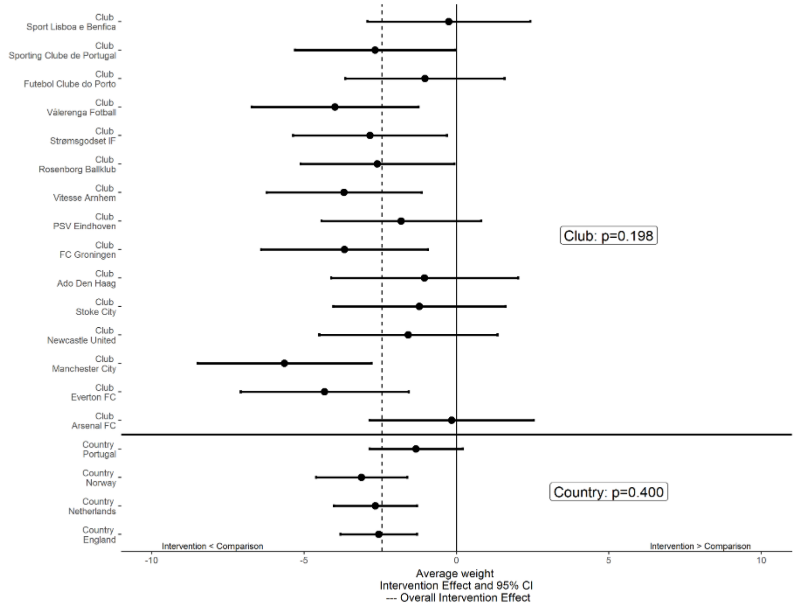

d)

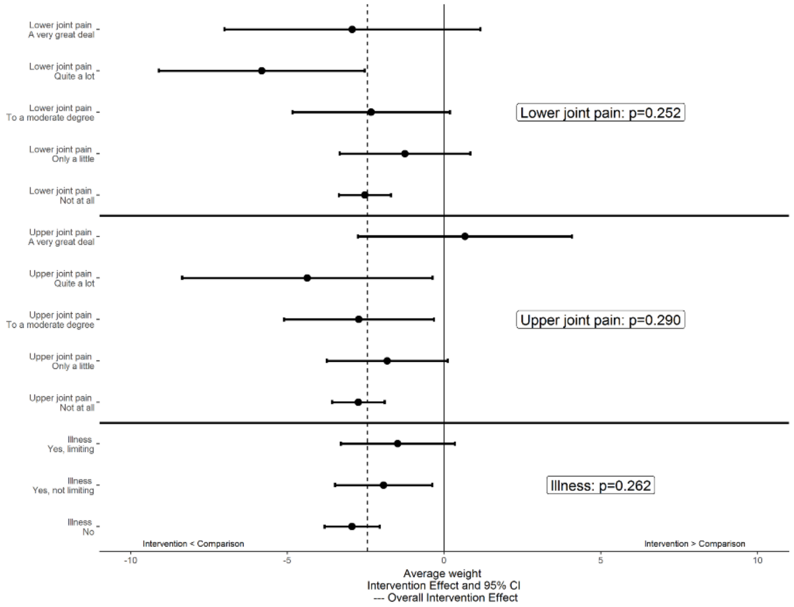

Figure C – subgroup effects, weight, a = age and BMI, b = Relationship status, years of education, income and employment, c = Country and club, d = Long standing illness, upper joint pain and lower joint pain score·
