# Supplementary material for: The effect of a programme to improve men’s sedentary time and physical activity: The European Fans in Training (EuroFIT) randomised controlled trial
Source: PLoS Med. 2019 Feb 5;16(2):e1002736. doi: 10.1371/journal.pmed.1002736 (PMC6363143; doi:10.1371/journal.pmed.1002736)
Supplement: S2 Fig — EuroFIT, European Fans in Training. (PDF) [file pmed.1002736.s010.pdf]

## S2 Fig. Probability of EuroFIT being cost-effective compared to the comparison group

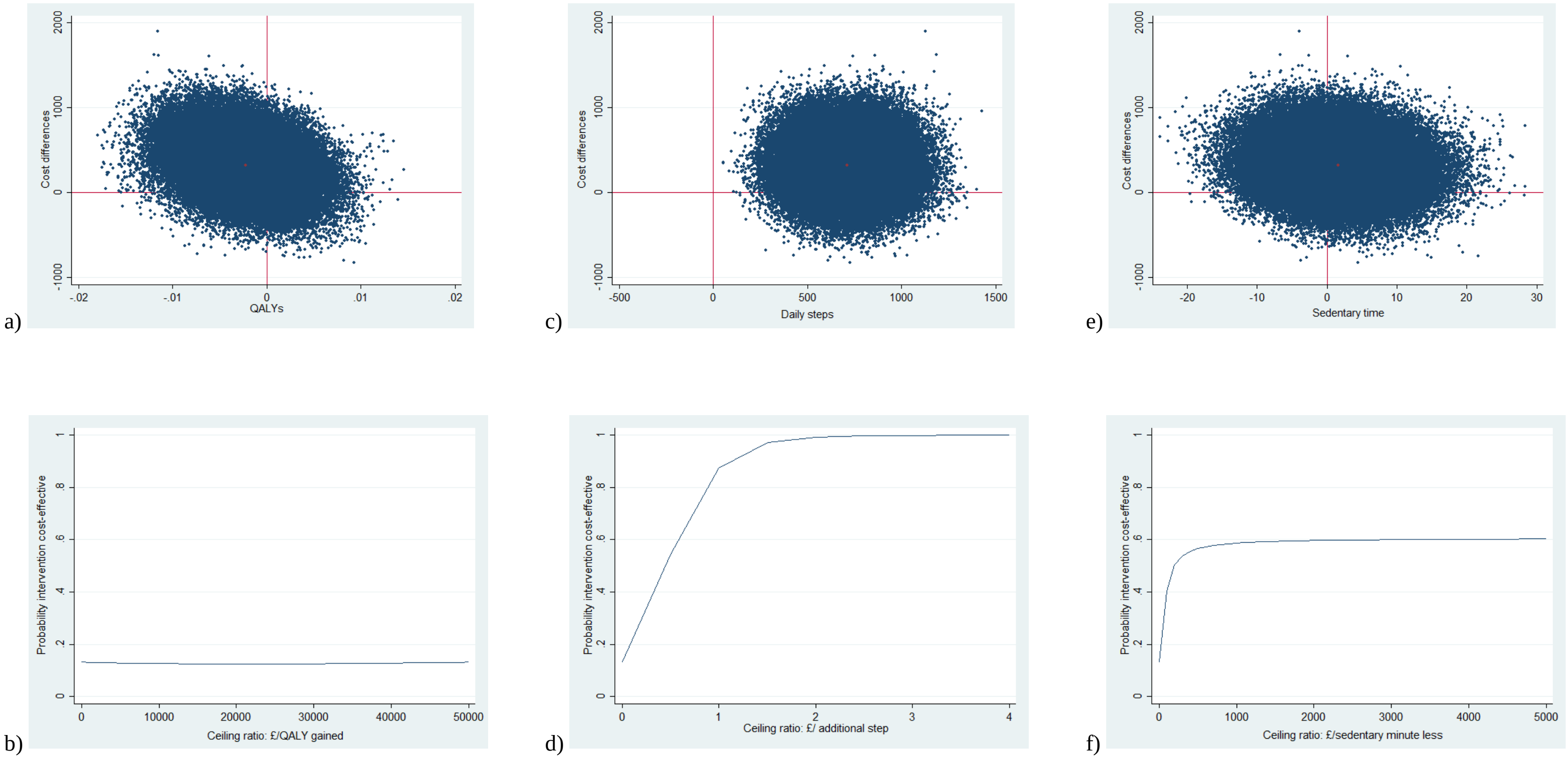


**Figure: Cost-effectiveness planes and cost effectiveness acceptability curves for each outcome: QALYs =a, b; Daily steps (activPAL)= c, d; Sedentary time (activPAL)= e, f; Meet physical activity guidelines (IPAQ)= g, h; Total physical activity (IPAQ)= I, j; ≥5% decrease in weight= k, l·**
